# The Dutch public are positive about the colorectal cancer-screening programme, but is this a well-informed opinion?

**DOI:** 10.1186/s12889-016-3870-7

**Published:** 2016-11-29

**Authors:** Linda N. Douma, Ellen Uiters, Danielle R. M. Timmermans

**Affiliations:** 1 Department of Public and Occupational Health, EMGO+ Institute for Health and Care research, VU University Medical Center, Van der Boechorststraat 7, 1081 BT Amsterdam, The Netherlands; 2 National Institute for Public Health and the Environment (RIVM), Postbus 1, 3720 BA Bilthoven, The Netherlands

**Keywords:** Colorectal cancer, Screening, Public, Informed opinion, Support, Knowledge, The Netherlands

## Abstract

**Background:**

Population-based colorectal cancer (CRC) screening is widely recommended, and members of the eligible screening population seem to be positive about it. However, it is not well known how people outside the eligible screening population view CRC screening, and whether they are supportive of the government providing this. Public opinion may affect people’s personal views and their screening decision.

The aim of our study was to examine the opinion of the Dutch general public regarding the national CRC screening programme.

**Method:**

An online survey was carried out in a Dutch population sample of adults aged 18 and older, assessing level of support, personal attitude, collective attitude, perceived social norm, awareness, and knowledge regarding the CRC screening programme.

**Results:**

The response rate was 56% (*n* = 1679/3000). Generally, the Dutch public are positive about and supportive of the CRC screening programme. We found the biggest proportion of support (86%) when people were asked directly. A smaller proportion (48%) was supportive when people had to choose between other options concerning how the government could possibly deal with CRC. People report knowing more about the benefits of CRC screening than about its possible harms and risks. Many people found it difficult to answer the knowledge questions that asked about numerical information concerning CRC screening correctly.

**Conclusion:**

People were less supportive of the CRC screening programme when having to choose between other options concerning dealing with CRC, and their support may not be based on a full comprehension of what CRC screening entails. Further research is needed to establish what knowledge people need in order to form a well-founded opinion.

**Electronic supplementary material:**

The online version of this article (doi:10.1186/s12889-016-3870-7) contains supplementary material, which is available to authorized users.

## Background

Colorectal cancer (CRC) is one of the most common causes of cancer death in developed countries [1, 2]. Population-based CRC screening can reduce the incidence and mortality of CRC [3–6], which is why it is widely recommended [7–10]. CRC screening involves potential benefits, but it also involves potential harms and risks (such as overdiagnosis, overtreatment, false negatives, false positives and risks associated with colonoscopy) [9–14].Whether for an individual the potential benefits weigh up against the potential harms and risks is a complex issue, involving information as well as personal values [15–17], which has been the topic of an ongoing debate among experts [13, 14, 17–19]. Therefore, it is seen as important that people are enabled to make a well-informed and personal decision concerning participation based on a good understanding of the potential benefits and harms of CRC screening as well as their personal preferences [15–17, 19, 20].

In general, people seem to be quite positive about CRC screening. Since the introduction of the CRC screening programme in the Netherlands in January 2014, which is provided by the Dutch government, relatively many people from the eligible screening population (adults aged 55–75) have participated (71%) [21]. In addition, the participation rates for the other cancer-screening programmes in the Netherlands (i.e. breast cancer screening and cervical cancer screening) have also been relatively high over the years (79% [22] and 64% [23], respectively), suggesting a positive view towards all forms of cancer screening. Several international studies into CRC screening suggest a generally positive view towards CRC screening as well [24–30]. It seems that most people believe that preventive screening for colorectal cancer is a good idea [24, 25, 28–30], important to do [27, 28] and saves lives [29, 30]. The majority of these studies, though, only studied the eligible screening population within the direct context of individual participation. They did not examine how the general public (which includes people inside as well as outside the eligible screening population) viewed CRC screening, with the exception of McCaffery et al. [26] and Bruel et al. [31]. Bruel et al. [31], however, did not examine how people view CRC screening in its entirety; they only assessed what percentage of overdetection (i.e. overdiagnosis and overtreatment) people found acceptable. Thus, although there seems to be a generally positive view towards CRC screening, it is not well known how people outside the eligible screening population, and irrespective of their own participation, view CRC screening.

There are two main reasons why the opinion of the general public towards CRC screening is of relevance. Firstly, public opinion (in addition to other factors) may – consciously or unconsciously – affect people’s personal views and attitude towards CRC screening and, consequently, their personal decision concerning participation [32–35]. Secondly, a considerable amount of Dutch government money is spent on the CRC screening programme. Thus, it seems important to gain more insight into whether the public are supportive of how this money is spent.

Public opinion is generally defined as the opinion of the majority or the dominant opinion within the public on a publicly relevant topic [29, 30, 33, 36–38]. Previous research into public opinion (mostly in the field of sociology or political science) generally assessed public opinion by determining the level of support [37, 39–41] and or attitude towards a certain issue or action [29, 30, 36, 37, 40, 41] among a large group of individuals representative for the public. Studies that assessed both level of support and attitude did this mostly based on the belief that people’s attitude towards a certain issue affects their level of support. When assessing people’s attitude, some studies made a distinction between people’s personal attitude and their collective attitude [33, 36]. Someone’s personal attitude reflects how they view and evaluate a certain issue or action while considering its implications for themselves (e.g. if they think that participating in CRC screening is a good idea for themselves or not). Someone’s collective attitude, however, reflects how they view and evaluate a certain issue or action while considering its implications for the population or society as a whole (e.g. if they think that CRC screening is a good idea for the Dutch population as a whole or not).

Previous research into cancer screening and public opinion showed that people’s attitude and level of support are often associated with several factors. Examining these factors and associations will provide a broader context for interpreting people’s opinion about CRC screening. The most common key factors seem to be people’s awareness, knowledge and perceived social norm regarding a certain issue. People’s views towards a certain issue are likely to be affected by how aware and knowledgeable they are of the issue [26, 40–43], and by how they perceive others like them to think and act regarding that issue (i.e. perceived social norm) [32, 39, 42, 44–46]. Furthermore, the gender, age and education of the eligible screening population are often found to be associated with differences in people’s individual attitudes towards, and knowledge of, CRC screening [26, 28, 42, 47, 48]. It seems likely then that these characteristics might also be associated with differences in public opinion and knowledge concerning the CRC screening programme.

Our study aims to examine the opinion of the Dutch general public (adults 18 and older) regarding the CRC screening programme. Specifically, we aim to answer the following research questions:

I. To what extent are the general public supportive of the CRC screening programme, from both a personal and a collective perspective? Are there differences associated with sociodemographic characteristics?
II. To what extent is the public opinion a well-informed opinion? Are there differences associated with sociodemographic characteristics?

## Methods

### Questionnaire and participants

We recruited participants via a national online research panel (Flycatcher Internet Research, www.flycatcher.eu; ISO 26362). Members of this panel sign up voluntarily to participate in online research. They can earn points, which they can eventually exchange for a gift card. The questionnaire was pre-tested among 36 members of the online panel; they were asked to comment on comprehensibility, difficulty, length and intrusiveness of the survey. After the pre-test some adjustments in wording and format were made. For our survey, 3000 panel members aged 18 and above, varying in education and geographic location, were invited via e-mail in December 2014 to complete our online questionnaire. The response rate was 56% (1679 participants).

### Measures

#### Level of support

We assessed support using three different question formats to provide a more comprehensive portrayal of support and to minimize socially desirable answers [49, 50]. We first asked participants if they thought it was good that the CRC screening programme existed in the Netherlands [37, 40] (5-point scale: 1 = *totally not good,* 5 = *totally good*). In addition, we asked participants in a more indirect way about their support for the CRC screening programme using a ranking question and a forced-choice question. In the ranking question, we presented participants with five possible ways the Dutch government could possibly deal with CRC (improving the treatment of CRC; offering preventive screening a.k.a. the CRC screening programme; conducting more research on the causes of CRC; providing public education about the symptoms and risk factors of CRC, and about what people can do themselves to decrease their risk of CRC; improving tools/methods to diagnose CRC) and asked them to rank these in order of importance (from 1 = *most important,* to 5 = *least important*). In the forced-choice question, we asked participants to choose whether they agreed or disagreed with four evaluative statements about the governmental costs of the CRC screening programme (I believe this is a good investment; this money would be better spent by the government on other issues in health care; this money would be better spent by the government on other ways to deal with CRC; the benefits of the CRC screening programme weigh up against the costs).

#### Personal and collective attitude

We assessed participants’ personal and collective attitude by asking them to evaluate the CRC screening programme on six dimensions using 5-point semantic differential scales (bad-good; disturbing-reassuring; not meaningful-meaningful; not self-evident-self-evident; not frightening-frightening; unimportant-important). These dimensions were derived from the 10-item attitude scale used by Van Dam [51]. We first assessed participants’ collective attitude by asking them what they thought of the CRC screening programme for the Dutch population (‘I believe the CRC screening programme to be … for the Dutch population’). Subsequently, we assessed participants’ personal attitude by asking them what they thought of the CRC screening programme for themselves (‘I believe participating in the CRC screening programme to be … for myself’).

#### Perceived social norm

Perceived social norm was assessed by presenting participants with four statements about their perception of how others are viewing the CRC screening programme [32] (I think that most people in my environment believe that the CRC screening programme is good/useful; I think that most people in the Netherlands believe that the CRC screening programme is good/useful) and asking them to what extent they agreed with each statement (5-point scales: 1 = *totally disagree,* 5 = *totally agree)*.

#### Awareness and sources of information

To assess awareness we asked participants if they had heard about the CRC screening programme. If they answered yes to this question, participants were asked if they had heard of the following topics related to the CRC screening programme: general information about CRC and the CRC screening programme; information about the stool-test procedure; participation in CRC screening being your own choice; the benefits of CRC screening; the CRC screening programme being offered by the government; information about the follow-up test (colonoscopy); the harms and risks of CRC screening; numerical information about CRC and the CRC screening programme; information about the stool-test quality; information about the potential costs for CRC screening participants; information about colonoscopy waiting lists. We then asked participants to indicate if they had heard of the CRC screening programme through any of the following information sources: television/radio; newspaper; people in their environment; online (news) websites; social media/online discussion forum; an invitation to participate in the programme; government website; their general practitioner; other.

#### Knowledge

Following the format of Siegrist and Cvetokovich [52], we asked people to report how much they thought they knew about: 1) the benefits of the CRC screening programme; 2) the risks and harms of the CRC screening programme; 3) the stool test and follow-up test as part of the CRC screening programme (three separate questions; 5-point scales: 1 = *(almost) nothing*, 5 = *very much*). Additionally, we asked people six more specific multiple-choice knowledge questions about general aspects of CRC screening (derived from several sources [10, 48, 53]). These questions consisted of two conceptual questions (about how much certainty the stool test provides) and four numerical questions (about the incidence of CRC, how many people die of CRC, how many deaths can be prevented by CRC screening, and the risk of getting CRC). See Additional file 1: Appendix A for a detailed description.

#### Sociodemographic characteristics

Data on gender, age and education (low, intermediate, high; according to the International Standard Classification of Education (ISCED), 2011) were gathered in order to examine whether there were any differences on the main variables associated with these characteristics.

### Statistical analysis

We assessed the Cronbach’s alpha for the forced-choice support question (.77), personal attitude (.85), collective attitude (.76), perceived social norm (.91) and self-rated knowledge (.86). The calculated total score for each of these variables (except the forced-choice support question) were the sum scores divided by the number of items, resulting in scores between one and five. The score for the forced-choice support question was calculated by adding the answers indicating support for the CRC screening programme, resulting in a total score ranging from one to five. With regard to the ranking support question, a mean ranking score was calculated for the CRC screening programme (and the other options part of the ranking) based on ranking placement and corresponding weight (with rank 1 having a weight of 5 and rank 5 having a weight of 1) and the number of people ranking the option on the same place. With regard to the specific multiple-choice knowledge questions, we calculated a total score based on how many questions were answered correctly.

For descriptive purposes, we assigned categories to the scores for support, attitude, perceived social norm and self-rated knowledge. Scores of four and five were classified as having: high support, a positive attitude, a positive perceived social norm, much knowledge. Scores of three were classified as having: moderate support, a neutral attitude, a neutral perceived social norm, not a little knowledge/not a lot of knowledge. Scores of one and two were classified as having: low support, a negative attitude, a negative perceived social norm, no or little knowledge. All analyses are based on the original range of scores (1–5) and not on the categories we assigned.

Descriptive statistics were calculated for all variables, and correlational analysis was used to examine possible associations between the variables. To examine differences related to gender, age or education we used multiple linear regression analysis (for the direct support question, forced-choice support question, personal attitude, collective attitude, perceived social norm, self-rated knowledge, and the specific multiple-choice knowledge questions), multiple logistic regression analysis (for the questions regarding awareness and information sources), and multiple multinomial logistic regression analysis (for the ranking support question). Age was entered as a continuous variable. All analyses were carried out using SPSS 22.0.

## Results

### Sample characteristics

A total of 1679 participants responded to our survey. Table 1 presents the sociodemographic characteristics of the study sample. The median age was 51 years old. People aged 65 years and older were overrepresented (25%). The distribution of the study sample with regard to gender, age, education and geographic location was representative of the Dutch adult population (i.e. the general public) based on data from Statistics Netherlands [54]. Non-response analysis showed that people were more likely to have participated in our survey when older, higher educated or male.

**Table 1:** Demographic characteristics of the sample population

	N (%)
Total	1679 (100)
*Gender*	
Men	903 (54)
Women	776 (46)
*Age category*	
18–24	123 (7)
25–34	235 (14)
35–44	258 (15)
45–54	362 (22)
55–64	285 (17)
65+	416 (25) ^a^
*Education*	
Low	544 (32)
Intermediate	681 (41)
High	454 (27)

^a^Total percentage is more than 100% due to rounding up

### Level of support

People were supportive of the CRC screening programme when asked directly (*M* = 4.12, *SD* = .69) as well as when using the forced-choice question (*M* = 4.18, *SD* = 1.23) or ranking question (*mean rank* = 3.22; see Additional file 1: Appendix B for an overview of all mean ranking scores). Table 2 shows the relevant frequencies and percentages. With regard to the direct support question, there was no difference related to gender, but people were less supportive when higher educated and more supportive when older (Table 3). With regard to the forced-choice question, people were more supportive when male (Table 3). There was no difference related to age or education. When looking at the calculated mean scores for the ranking question, we see that people are more supportive when female or older and less supportive when higher educated. Multinomial logistic regression analysis was carried out to further examine these differences. We found that women more often than men place the CRC screening programme at number 2 in the ranking and less often at number 4. When older, people place the CRC screening programme more often at number 1 in the ranking and less often at number 5. When higher educated, people place the CRC screening programme less often at number 1 in the ranking and more often at number 4 (data not shown, see Additional file 2).

**Table 2:** Level of support, attitude and perceived social norm regarding CRC screening programme

Variables^a,b^	N (%)
Support CRC screening programme	
*Direct question*	
Low support (score <3)	28 (2)
Moderate support (score = 3)	211 (12)
High support (score >3)	1440 (86)
*Forced-choice question*	
Low support (score <3)	192 (11)
Moderate support (score = 3)	193 (12)
High support (score >3)	1294 (77)
*Ranking question*	
Low support (ranked at No. 4 or 5)	592 (35)
Moderate support (ranked at No. 3)	283 (17)
High support (ranked at No. 1 or 2)	804 (48)
Attitude CRC screening programme	
*Personal attitude*	
Negative attitude (score <3)	120 (7)
Neutral attitude (score = 3)	131 (8)
Positive attitude (score >3)	1428 (85)
*Collective attitude*	
Negative attitude (score <3)	54 (3)
Neutral attitude (score = 3)	65 (4)
Positive attitude (score >3)	1560 (93)
Perceived social norm CRC screening programme	
Negative perceived social norm (score <3)	38 (2)
Neutral perceived social norm (score = 3)	161 (10)
Positive perceived social norm (score >3)	1480 (88)

^a^Total *n* = 1679

^b^Minimum score: 1, maximum score: 5

**Table 3:** Associations between sociodemographic characteristics and support, attitude, perceived social norm and knowledge (multiple linear regression analysis)

Variables	B	95% CI
Support – Direct question		
Gender ^a^	.055	-.012–.123
Age ^b^	.002	.000–.005
Education^d^		
Intermediate	-.030	-.110–.050
High	-.177*	-.266–-.088
Support – Forced-choice question		
Gender ^a^	-.141*	-.262–-.020
Age ^b^	.003	-.001–.007
Education ^d^		
Intermediate	-.042	-.185–.102
High	-.111	-.271–.049
Personal attitude		
Gender ^a^	.124*	.051–.196
Age ^b^	.011**	.009–.013
Education ^d^		
Intermediate	.008	-.078–.093
High	-.120*	-.215–-.024
Collective attitude		
Gender ^a^	.059	.000–.118
Age ^b^	.004**	.002–.005
Education ^d^		
Intermediate	-.044	-.114–.025
High	-.162**	-.240–-.084
Perceived social norm		
Gender ^a^	.010	-.046–.066
Age ^b^	.000	-.002–.002
Education ^d^		
Intermediate	-.053	-.120–.014
High	-.065	-.139–.010
Self-rated knowledge		
Gender ^a^	.167**	.085–.248
Age ^b^	.019**	.016–.022
Education ^d^		
Intermediate	.075	-.121–.171
High	.117*	.010–.225
Specific multiple-choice knowledge questions		
Gender ^a^	-.058	-.168–.051
Age ^b^	-.001	-.004–.003
Education ^d^		
Intermediate	.193*	.064–.323
High	.280**	.136–.425

^a^Reference group is men

^b^Age was entered as a continuous variable

^d^Reference group is low education

*Significant at level *p* < .05

**Significant at level *p* < .001

### Personal and collective attitude

People hold a positive personal (*M* = 4.06, *SD* = .76) and collective attitude (*M* = 4.07, *SD* = .60) towards the CRC screening programme (see Table 2 for the relevant frequencies and percentages). People hold a more positive personal attitude when female, older or lower educated (Table 3). With regard to collective attitude, we also found people to be more positive when older or lower educated, but there was no difference related to gender.

### Perceived social norm

People perceived others like them to be positive towards the CRC screening programme (*M* = 3.95, *SD* = .57; see Table 2 for the relevant frequencies and percentages). We found no significant difference associated with gender, age or education (Table 3).

### Awareness and sources of information

Eighty percent of respondents (*N* = 1348) had heard of the CRC screening programme, and 61% (*N* = 1021) also knew what the CRC screening programme entailed. Table 4 shows which specific topics people had heard about and which information sources were used. Those that had heard of the CRC screening programme mostly reported hearing about general information on CRC and CRC screening (74%). People reported hearing more about the benefits of CRC screening (55%) than about the possible harms and risks of CRC screening (22%). People had heard about the CRC screening programme mostly through traditional media (television/radio 55%, and newspaper 36%). People had heard more often of the CRC screening programme when female (*OR* = 1.361, *95% CI:* 1.048 ─ 1.768), older (*OR* = 1.061, *95% CI:* 1.051 ─ 1.071) or higher educated (intermediate vs. low education *OR* = 1.377, *95% CI*: .994–1.909, *p* = .054; high vs. low education *OR* = 1.410, *95% CI*: .992–2.004, *p* = .055). Table 5 shows any differences related to gender, age or education in awareness and information sources used. People had heard more often of many of the specific topics when female, older or higher educated. When older, people used the newspaper and television/radio more often as an information source, and websites and social media less often.

**Table 4:** Awareness, information sources and knowledge regarding CRC screening programme

Variables	N (%)
Awareness^b^	
*[Q: What have you heard about the CRC screening programme?]*	
General information about CRC and the CRC screening programme	993 (74)
Information about the stool-test procedure	783 (58)
Participating in CRC screening is your own choice	752 (56)
What the benefits of CRC screening are	744 (55)
That the CRC screening programme is offered by the government	703 (52)
Information about the follow-up test (colonoscopy)	530 (39)
What the harms and risks of CRC screening are	293 (22)
Numerical information about CRC and the CRC screening programme	250 (18)
Information about the stool-test quality	184 (14)
Information about the potential costs for CRC screening participants	123 (9)
Information about colonoscopy waiting lists	61 (4)
None of the above	76 (6) ^a^
Information sources^b^	
*[Q: Where did you hear about the CRC screening programme?]*	
Television/radio	743 (55)
Newspaper	492 (36)
People in my environment	416 (31)
Online (news) websites	257 (19)
Invitation to participate	166 (12)
Government website	48 (4)
Social media/online (discussion) forum	40 (3)
General practitioner	39 (3)
Other	65 (5) ^a^
Self-rated knowledge about the CRC screening programme^a,d^	
No or little knowledge (score <3)	594 (35)
Not little/not much knowledge (score = 3)	435 (26)
Much knowledge (score >3)	650 (39)
Looking at the three items of self-rated knowledge separately:	
*Knowledge on benefits of CRC screening programme*	
No or little knowledge (score <3)	342 (20)
Not little/not much knowledge (score = 3)	662 (40)
Much knowledge (score >3)	675 (40)
*Knowledge on harms and risks of CRC screening programme*	
No or little knowledge (score <3)	565 (34)
Not little/not much knowledge (score = 3)	759 (45)
Much knowledge (score >3)	355 (21)
*Knowledge on procedure of CRC screening programme*	
No or little knowledge (score <3)	499 (30)
Not little/not much knowledge (score = 3)	632 (37)
Much knowledge (score >3)	548 (33)
Specific multiple-choice knowledge questions about CRC screening programme^a^ *(answered correctly)*	
Approximately how many people a year in the Netherlands get colon cancer?	834 (50)
Approximately how many people a year in the Netherlands die of colon cancer?	613 (36)
Approximately how many colon cancer deaths a year in the Netherlands can be prevented with the CRC screening programme?	429 (26)
What do you think is the risk of an average Dutch person getting colon cancer during their lifetime?	807 (48)
If the stool test shows positive for blood, is it 100% certain that someone has colon cancer?	1642 (98)
If the stool test shows negative for blood, is it 100% certain that someone does not have colon cancer?	1632 (97)

^a^Total *n* = 1679

^b^Total *n* = 1348 (those who had heard of the CRC screening programme)

^c^Total percentage is more than 100% because multiple answers were possible

^d^Minimum score: 1, maximum score: 5

**Table 5:** Associations between sociodemographic characteristics and awareness and information sources (multiple logistic regression analysis)

Variables	OR	95% CI
Information heard: General information about CRC and the CRC screening programme		
Gender ^a^	1.068	.830–1.375
Age ^b^	1.018**	1.009–1.027
Education ^d^		
Intermediate	1.808**	1.352–2.416
High	2.573**	1.818–3.640
Information heard: Information about the stool-test procedure		
Gender ^a^	1.894**	1.492–2.405
Age ^b^	1.042**	1.034–1.051
Education ^d^		
Intermediate	1.135	.864–1.492
High	1.453*	1.065–1.981
Information heard: Participating in CRC screening is your own choice		
Gender ^a^	1.867**	1.480–2.355
Age ^b^	1.031**	1.023–1.039
Education ^d^		
Intermediate	1.313*	1.005–1.716
High	1.371*	1.015–1.854
Information heard: What the benefits of CRC screening are		
Gender ^a^	1.509**	1.198–1.901
Age ^b^	1.035**	1.027–1.043
Education ^d^		
Intermediate	1.275	.976–1.667
High	1.467*	1.084–1.987
Information heard: That the CRC screening programme is offered by the government		
Gender ^a^	1.550**	1.235–1.945
Age ^b^	1.026**	1.018–1.034
Education ^d^		
Intermediate	1.426*	1.095–1.856
High	1.760**	1.304–2.374
Information heard: Information about the follow-up test (colonoscopy)		
Gender ^a^	1.493*	1.181–1.886
Age ^b^	1.031**	1.023–1.040
Education ^d^		
Intermediate	1.348*	1.029–1.767
High	1.577*	1.161–2.143
Information heard: What the harms and risks of CRC screening are		
Gender ^a^	1.084	.823–1.429
Age ^b^	1.024**	1.014–1.034
Education ^d^		
Intermediate	1.434*	1.033–1.990
High	2.309**	1.620–3.292
Information heard: Numerical information about CRC and the CRC screening programme		
Gender ^a^	1.043	.780–1.396
Age ^b^	1.021**	1.011–1.032
Education ^d^		
Intermediate	1.011	.719–1.422
High	1.645*	1.143–2.370
Information heard: Information about the stool-test quality		
Gender ^a^	1.019	.729–1.423
Age ^b^	1.035**	1.022–1.047
Education ^d^		
Intermediate	1.198	.820–1.750
High	1.482	.972–2.260
Information heard: Information about the potential costs for CRC screening participants		
Gender ^a^	.985	.666–1.458
Age ^b^	1.022*	1.008–1.036
Education ^d^		
Intermediate	1.240	.799–1.926
High	1.095	.654–1.835
Information heard: Information about colonoscopy waiting lists		
Gender ^a^	1.266	.735–2.181
Age ^b^	1.033*	1.013–1.054
Education ^d^		
Intermediate	1.214	.663–2.224
High	1.139	.557–2.332
Source of information: Newspaper		
Gender ^a^	.909	.716–1.154
Age ^b^	1.036**	1.028–1.045
Education ^d^		
Intermediate	1.419*	1.074–1.874
High	2.148**	1.569–2.942
Source of information: Television/radio		
Gender ^a^	1.123	.897–1.407
Age ^b^	1.017**	1.010–1.025
Education ^d^		
Intermediate	1.210	.930–1.574
High	.821	.612–1.101
Source of information: People in my environment		
Gender ^a^	1.314*	1.033–1.672
Age ^b^	.977**	.969–.985
Education ^d^		
Intermediate	.818	.614–1.091
High	.920	.668–1.266
Source of information: Online (news) websites		
Gender ^a^	.667*	.501–.888
Age ^b^	.979**	.970–.988
Education ^d^		
Intermediate	.893	.638–1.249
High	.929	.642–1.344
Source of information: Invitation to participate		
Gender ^a^	.868	.591–1.274
Age ^b^	1.096**	1.077–1.116
Education ^d^		
Intermediate	1.051	.707–1.562
High	.879	.538–1.437
Source of information: Government website		
Gender ^a^	.863	.476–1.565
Age ^b^	.997	.977–1.017
Education ^d^		
Intermediate	.943	.499–1.782
High	.300*	.109–.827
Source of information: Social media/online (discussion) forum		
Gender^a^	.960	.506–1.824
Age^b^	.968*	.948–.988
Education^d^		
Intermediate	.855	.401–1.822
High	.603	.246–1.482
Source of information: General practitioner		
Gender^a^	.608	.299–1.234
Age^b^	1.025*	1.000–1.051
Education^d^		
Intermediate	.548	.256–1.174
High	.617	.262–1.456

^a^Reference group is men

^b^Age was entered as a continuous variable

^d^Reference group is low education

*Significant at level *p* < .05

** Significant at level *p* < .001

### Knowledge

The mean score for self-rated knowledge was 2.98 (*SD* = .87), indicating that people did not think they had little knowledge about the CRC screening programme, but also not a lot (see Table 4 for the relevant frequencies and percentages). When looking at the three questions separately, we see that people reported knowing most about the benefits of the CRC screening programme (*M* = 3.21, *SD* = .94) and least about the harms and risks (*M* = 2.75, *SD* = .97). On average, people answered three or four of the specific multiple-choice knowledge questions correctly (*M* = 3.55, *SD* = 1.11). Almost everyone correctly answered the two conceptual questions on how much certainty the stool test gives about having colon cancer (98%) or not having colon cancer (97%). People’s self-rated knowledge was higher when female, older or higher educated (Table 3). With regard to the specific knowledge questions, we found no difference related to gender or age, but more questions were answered correctly when higher educated.

### Associations between key components

We assessed support using different question formats. The direct support question was positively correlated with personal attitude (*r* = .57, *p* < .001), collective attitude (*r* = .59, *p* < .001) and perceived social norm (*r* = .51, *p* < .001). The forced-choice support question was also positively correlated with personal attitude (*r* = .44, *p* < .001), collective attitude (*r* = .44, *p* < .001) and perceived social norm (*r* = .33, *p* < .001). Personal and collective attitude were highly correlated (*r* = .78, *p* < .001). Perceived social norm was positively correlated with personal attitude (*r* = .48, *p* < .001) and collective attitude (*r* = .52, *p* < .001). Awareness, self-rated knowledge and correctly answering the specific multiple-choice knowledge questions were weakly or non-significantly correlated to each other as well as with support, attitude and perceived social norm (also when looking at the awareness topics and self-rated knowledge questions separately; data not shown, see Additional file 3).

## Discussion

In our study, we found that the Dutch public are in general positive about and supportive of the CRC screening programme. People do not seem to differ in their evaluation of the personal and societal implications of the CRC screening programme, and perceive others to be positive about the programme as well. The majority of the Dutch public (80%) was aware of the CRC screening programme, but most reported knowing more about the benefits of CRC screening than about its possible harms and risks. Level of support, attitude and perceived social norm were positively associated with each other.

Our findings seem to be in line with previous research into CRC screening, which also found people to be generally positive about CRC screening. However, previous studies mainly examined individual attitudes of the eligible screening population [24, 29, 30]. Our results indicate that the positive view towards CRC screening is not limited to those eligible for screening.

Although the majority of the Dutch public is supportive of the CRC screening programme, we did find substantial variations between the three formats used to assess support. We found the biggest proportion of support (86%) when asked directly and the smallest proportion when using the ranking question (48%). Apparently, people value the CRC screening programme less when explicitly being asked to consider that there are limited possibilities and resources and to compare the importance of the CRC screening programme to other options concerning dealing with CRC. Thus, people might believe the CRC screening programme to be a good idea in itself, but when having to choose between other options to deal with CRC, the CRC screening programme is not necessarily the option everyone would choose [55, 56].

The overall positive view of the Dutch public towards the CRC screening programme might in part be explained by the finding that people were more aware of and knowledgeable about the benefits of CRC screening than about its possible harms and risks. Previous research into CRC screening also shows that people are generally more aware of the benefits of CRC screening than of the harms and risks [28–31, 42]. This is not surprising because, until recently, the communication about cancer screening focused on screening being beneficial and a good thing to do [11–13, 57, 58]. Nowadays, fostering making informed decisions [19], the potential benefits as well as the harms and risks of CRC screening are mentioned in the public communication by the Dutch government [59]. However, we found that for the general public the main source of information is not the communication by the government, but traditional media. They may filter or frame their message in a certain way [60, 61], affecting what information people receive and remember best [34, 60, 61].

That most people are less aware of the harms and risks of CRC screening raises the question of whether they would still be as supportive if they knew more about the potential negative sides of CRC screening. Correlations based on our complete sample do not conclusively indicate that people are less supportive of CRC screening when more aware of its negative sides. However, among higher educated people, we found more awareness of the harms and risks and more knowledge about CRC screening in general, and they were also less supportive and positive about the CRC screening programme. Earlier research into the relationship between knowledge (on both benefits and harms) and attitude regarding CRC screening shows mixed results [26, 28, 42]. Drawing definitive conclusions concerning the relationship between knowledge and attitude is complicated by the fact that there does not seem to be an agreement on what people ought to know about CRC screening and when people have good or sufficient knowledge. Studies use different knowledge questions [26, 28, 42, 48, 62], different outcome measures [26, 48, 62] and different cut-off points [48, 62]. People in our study answered about half to two-thirds of our specific multiple-choice knowledge questions correctly. Additionally, they rated themselves as knowing not little, but also not much about CRC screening. Without a clear definition on what is seen as sufficient knowledge, it is difficult to say whether participants in our study had sufficient knowledge about CRC screening to base their views and opinion on.

Besides having knowledge about all aspects of CRC screening, it is also important that people fully understand this information and are able to use it in forming an opinion or making a decision. These ‘health literacy’ skills are often found to be associated with cancer screening participation [63, 64]. Higher educated people might be better able to handle the complex information associated with CRC cancer screening [12, 19, 65, 66]. Previous research into risk information and risk communication shows that most people find it difficult to interpret and understand information about risks, probabilities and weighing up pros and cons, especially when it is presented numerically [28, 67, 68]. In our study, we also found that people had the most difficulty when answering the knowledge questions that asked about numerical information concerning CRC screening and not with the more conceptual questions. Thus, although people are supportive of the CRC screening programme, this may not be based on a full comprehension of what CRC screening entails.

A limitation of our study is that our specific multiple-choice knowledge questions asked about the main general aspects of CRC screening. We did not include a broad range of questions about the specific benefits and the specific harms and risks. Therefore, we could not examine whether having more ‘objective’ knowledge (compared to the more ‘subjective’ self-rated knowledge we did assess) on the benefits or on the harms and risks might have been related to having a more or less positive view towards CRC screening. Another limitation might be that we used a random sample of members of a national internet panel as participants. People who participate in online research may differ in significant ways from people who do not participate in online research. They might be, for example, more interested in or positive towards CRC screening to begin with, possibly resulting in an overestimation of public support for the CRC screening programme. Thirdly, we examined whether there were any differences in public opinion related to gender, age and education. However, other sociodemographic characteristics may also be associated with differences in public opinion. For example, among the eligible CRC screening population, people with a lower income, lower social-economic status or belonging to a minority group are regularly found to think less positive about CRC screening [42, 47]. Future research could focus on examining how sociodemographic characteristics other than gender, age and education might be associated with differences in public opinion. A strength of our study is that we used three different question formats to assess level of support, providing a more comprehensive portrayal of support.

## Conclusion

The Dutch public are supportive of the CRC screening programme, although not everyone might be as supportive when having to choose between other options concerning dealing with CRC. People’s support for the CRC screening programme may not be based on sufficient knowledge or a full comprehension of what screening entails. Future research could focus on examining what kind and amount of knowledge people need to have in order to form a well-founded opinion, and how to ensure that people are adequately informed.

## Additional files

Additional file 1: Appendix A. Specific multiple-choice knowledge questions. **Appendix B.**Overview of mean ranking scores regarding the ranking support question. (DOCX 16 kb)
Additional file 2: Multinomial regression analyses ranking support question_spss output. (DOCX 326 kb)
Additional file 3: Correlations key components_spss output. (DOCX 58 kb)

## References

[1] World Health Organization. Globocan 2012: Estimated cancer incidence, mortality and prevalence worldwide in 2012. [http://globocan.iarc.fr/Pages/fact_sheets_population.aspx]. Accessed 2 Nov 2015.

[2] Integraal Kankercentrum Nederland. Nederlandse Kankerregistratie [Dutch Cancer Registration]. [http://www.cijfersoverkanker.nl]. Accessed 16 July 2015.

[3] Hardcastle JD, Chamberlain JO, Robinson MH (1996). Randomised controlled trial of faecal-occult-blood screening for colorectal cancer. Lancet.

[4] Kronborg O, Fenger C, Olsen J, Jorgensen OD, Sondergaard O (1996). Randomised study of screening for colorectal cancer with faecaloccult-blood test. Lancet.

[5] Mandel JS, Bond JH, Church TR (1993). Reducing mortality from colorectal cancer by screening for fecal occult blood. Minnesota Colon Cancer Control Study. N Engl J Med.

[6] Winawer SJ, Zauber AG, Ho MN (1993). Prevention of colorectal cancer by colonoscopic polypectomy. The National Polyp Study Workgroup. N Engl J Med.

[7] Commission of the European Communities (2003). Council Recommendation on Cancer Screening.

[8] Sung JJ, Lau JY, Young GP (2008). Asia Pacific consensus recommendations for colorectal cancer screening. Gut.

[9] Bibbins-Domingo K, Grossman DC, Curry SJ, Davidson KW, Epling JW, Garcia FA, Gillman MW, Harper DM, Kemper AR, Krist AH (2016). Screening for Colorectal Cancer: US Preventive Services Task Force Recommendation Statement. JAMA.

[10] Gezondheidsraad (Health Council of the Netherlands) (2009). Bevolkingsonderzoek naar darmkanker.

[11] Gray JA, Patnick J, Blanks RG (2008). Maximising benefit and minimising harm of screening. BMJ.

[12] Whitlock EP, Lin J, Liles E, Beil T, Fu R, O’Connor E, Thompson RN, Cardenas T (2008). U.S. Preventive Services Task Force Evidence Syntheses, formerly Systematic Evidence Reviews. Screening for Colorectal Cancer: An Updated Systematic Review.

[13] Saquib N, Saquib J, Ioannidis JP (2015). Does screening for disease save lives in asymptomatic adults? Systematic review of meta-analyses and randomized trials. Int J Epidemiol.

[14] Fitzpatrick-Lewis D, Ali MU, Warren R, Kenny M, Sherifali D, Raina P: Screening for Colorectal Cancer: A Systematic Review and Meta-Analysis. Clin Colorectal Cancer 2016. doi: 10.1016/j.clcc.2016.03.003 10.1016/j.clcc.2016.03.003 27133893

[15] Irwig L, McCaffery K, Salkeld G, Bossuyt P (2006). Informed choice for screening: implications for evaluation. BMJ.

[16] Jepson RG, Hewison J, Thompson A, Weller D (2007). Patient perspectives on information and choice in cancer screening: a qualitative study in the UK. Soc Sci Med.

[17] Johansson M, Brodersen J (2015). Informed choice in screening needs more than information. Lancet.

[18] Gotzsche P (2015). Commentary: Screening: a seductive paradigm that has generally failed us. Int J Epidemiol.

[19] Rimer BK, Briss PA, Zeller PK, Chan EC, Woolf SH (2004). Informed decision making: what is its role in cancer screening?. Cancer.

[20] Marteau TM, Dormandy E, Michie S (2001). A mesaure of informed choice. Health Expect.

[21] van Leerdam ME, Toes E, Spaander VMCM, van Vuuren AJ, Dekker E, Kuipers EJ (2014). Eerste resultaten bevolkingsonderzoek darmkanker.

[22] LETB (2014). Landelijke evaluatie van bevolkingsonderzoek naar borstkanker in Nederland 1990-2011/2012. Het dertiende evaluatierapport. Maatschappelijke Gezondheidszorg.

[23] LEBA (2012). Landelijke Evaluatie Bevolkingsonderzoek naar Baarmoederhalskanker 2011.

[24] Cullati S, Charvet-Berard AI, Perneger TV (2009). Cancer screening in a middle-aged general population: factors associated with practices and attitudes. BMC Public Health.

[25] Hall NJ, Rubin GP, Dobson C, Weller D, Wardle J, Ritchie M, Rees CJ: Attitudes and beliefs of non-participants in a population-based screening programme for colorectal cancer. Health Expect. 2013;18:1645–1657. 10.1111/hex.12157 PMC5060871 24268129

[26] McCaffery K, Wardle J, Waller J (2003). Knowledge, attitudes, and behavioral intentions in relation to the early detection of colorectal cancer in the United Kingdom. Prev Med.

[27] Murphy CC, Vernon SW, Haddock NM, Anderson ML, Chubak J, Green BB (2014). Longitudinal predictors of colorectal cancer screening among participants in a randomized controlled trial. Prev Med.

[28] Smith SK, Simpson JM, Trevena LJ, McCaffery KJ (2014). Factors Associated with Informed Decisions and Participation in Bowel Cancer Screening among Adults with Lower Education and Literacy. Med Decis Mak.

[29] Schwartz LM, Woloshin S, Fowler FJ, Welch HG (2004). Enthusiasm for cancer screening in the United States. J Am Med Assoc.

[30] Waller J, Osborne K, Wardle J (2015). Enthusiasm for cancer screening in Great Britain; a general population study. Br J Cancer.

[31] van den Bruel A, Jones C, Yang Y, Oke J, Hewitson P (2015). People’s willingness to accept overdetection in cancer screening: population survey. BMJ.

[32] Park HS, Smith SW (2007). Distinctiveness and Influence of Subjective Norms, Personal Descriptive and Injunctive Norms, and Societal Descriptive and Injunctive Norms on Behavioral Intent: A Case of Two Behaviors Critical to Organ Donation. Hum Commun Res.

[33] Mutz DC (1998). Impersonal Influence: How Perceptions of Mass Collectives Affect Political Attitudes.

[34] Fiske ST, Taylor SE (1991). Social cognition.

[35] Noelle-Neumann E (1974). The spiral of silence: a theory of public opinion. J Commun.

[36] Soroka S, Maioni A, Martin P (2013). What moves public opinion on health care? Individual experiences, system performance, and media framing. J Health Polit Policy Law.

[37] Bleackley A, Hennessy M, Fishbein M (2006). Public Opinion on Sex Education in US Schools. Arch Pediatr Adolesc Med.

[38] Corbett M (1991). American public opinion: Trends, processes, and patterns.

[39] Hoffman LH, Glynn CJ, Huge ME, Sietman RB, Thomson T (2007). The Role of Communication in Public Opinion Processes: Understanding the Impacts of Intrapersonal, Media, and Social Filters. Int J Public Opin Res.

[40] Nisbet M, Markowitz EM (2014). Understanding Public Opinion in Debates over Biomedical Research: Looking beyond Political Partisanship to Focus on Beliefs about Science and Society. PLoS One.

[41] Ho SS, Brossard D, Scheufele DA (2008). Effects of Value Predispositions, Mass Media Use, and Knowledge on Public Attitudes Toward Embryonic Stem Cell Research. Int J Public Opin Res.

[42] Beydoun HA, Beydoun MA (2008). Predictors of colorectal cancer screening behaviors among average-risk older adults in the United States. Cancer Causes Control.

[43] Power E, Miles A, Von Wagner C, Robb K, Wardle J (2009). Uptake of colorectal cancer screening: system, provider and individual factors and strategies to improve participation. Future Oncol.

[44] Wardle J, McKaffery K, Nadel M, Atkin W (2004). Socioeconomic differences in cancer screening participation: comparing cognitive and psychosocial explanations. Soc Sci Med.

[45] Smith-McLallen A, Fishbein M (2008). Predictors of intentions to perform six cancer-related behaviours: Roles for injunctive and descriptive norms. Psychol Health Med.

[46] Ajzen I (1991). The theory of planned behavior. Organ Behav Hum Decis Process.

[47] von Wagner C, Baio G, Raine R, Snowball J, Morris S, Atkin W, Obichere A, Handley G, Logan RF, Rainbow S (2011). Inequalities in participation in an organized national colorectal cancer screening programme: results from the first 2.6 million invitations in England. Int J Epidemiol.

[48] Denters MJ, Deutekom M, Essink-Bot ML, Bossuyt PM, Fockens P, Dekker E (2013). Assessing knowledge and attitudes towards screening among users of faecal immunochemical test. Health Expect.

[49] Fisher RJ (1993). Social desirability bias and the validity of indirect questioning. J Consum Res.

[50] Nederhof AJ (1985). Methods of coping with social desirability bias: a review. Eur J Soc Psychol.

[51] van Dam L, Korfage IJ, Kuipers EJ, Hol L, van Roon AH, Reijerink JC, van Ballegooijen M, van Leerdam ME (2013). What influences the decision to participate in colorectal cancer screening with faecal occult blood testing and sigmoidoscopy?. Eur J Cancer.

[52] Siegrist M, Cvetokovich G (2000). Perception of hazards: the role of social trust and knowledge. Risk Anal.

[53] Brewer NT, Chapman GB, Gibbons FX, Gerrard M, McCaul KD, Weinstein ND (2007). Meta-analysis of the relationship between risk perception and health behavior: the example of vaccination. Health Psychol.

[54] CBS [Dutch Statistics]. MOA Golden Standard. [www.moaweb.nl/services/services/gouden-standaard.html]. Accessed 9 Apr 2015.

[55] Oliver A (2006). Further evidence of preference reversals: choice, valuation and ranking over distributions of life expectancy. J Health Econ.

[56] Slothuus U, Larsen ML, Junker P (2002). The contingent ranking method--a feasible and valid method when eliciting preferences for health care?. Soc Sci Med.

[57] Gotzsche PC, Nielsen M (2011). Screening for breast cancer with mammography. Cochrane Database Syst Rev.

[58] Gigerenzer G (2014). Breast cancer screening pamphlets mislead women. BMJ.

[59] RIVM. Bevolkingsonderzoek darmkanker [CRC screening programme]. [http://www.rivm.nl/Onderwerpen/B/Bevolkingsonderzoek_darmkanker]. Accessed 16 Jan 2016.

[60] Goffman E (1974). Frame Analysis: An Essay on the Organization of Experience.

[61] Chong D, Druckman JN (2007). Framing Theory. Annu Rev Polit Sci.

[62] de Haan MC, de Wijkerslooth TR, Stoop E, Bossuyt P, Fockens P, Thomeer M, Kuipers EJ, Essink-Bot ML, van Leerdam ME, Dekker E (2013). Informed decision-making in colorectal cancer screening using colonoscopy or CT-colonography. Patient Educ Couns.

[63] Oldach BR, Katz ML (2014). Health literacy and cancer screening: a systematic review. Patient Educ Couns.

[64] van der Heide I, Uiters E, Jantine Schuit A, Rademakers J, Fransen M (2015). Health literacy and informed decision making regarding colorectal cancer screening: a systematic review. Eur J Pub Health.

[65] Crowson CS, Therneau TM, Matteson EL, Gabriel SE (2007). Primer: Demystifying risk--understanding and communicating medical risks. Nat Clin Pract Rheumatol.

[66] Dillard AJ, Couper MP, Zikmund-Fisher BJ (2010). Perceived risk of cancer and patient reports of participation in decisions about screening: the DECISIONS study. Med Decis Mak.

[67] Gigerenzer G, Hertwig R, van den Broek E, Fasolo B, Katsikopoulos KV (2005). “A 30% chance of rain tomorrow”: how does the public understand probabilistic weather forecasts?. Risk Anal.

[68] Zipkin DA, Umscheid CA, Keating NL, Allen E, Aung K, Beyth R, Kaatz S, Mann DM, Sussman JB, Korenstein D (2014). Evidence-based risk communication: a systematic review. Ann Intern Med.

