# Supplementary material for: The Dutch public are positive about the colorectal cancer-screening programme, but is this a well-informed opinion?
Source: BMC Public Health. 2016 Nov 29;16:1208. doi: 10.1186/s12889-016-3870-7 (PMC5129673; doi:10.1186/s12889-016-3870-7)
Supplement: Additional file 1: Appendix A. — Specific multiple-choice knowledge questions. Appendix B.Overview of mean ranking scores regarding the ranking support question. (DOCX 16 kb) [file 12889_2016_3870_MOESM1_ESM.docx]

**Manuscript Public opinion CRC screening_LDouma**

**Additional material**

**Additional file 1**

**Appendix A: Specific multiple-choice knowledge questions**

| 1. Approximately how many people a year in the Netherlands get colon cancer? 2. 2,000 3. 5,000 4. 13,000 5. 21,000 6. Approximately how many people a year in the Netherlands die of colon cancer? 7. 1,000 8. 5,000 9. 7,000 10. 10,000 11. Approximately how many colon cancer deaths a year in the Netherlands can be prevented with the CRC screening programme? 12. 500 13. 1,200 14. 1,750 15. 2,400 16. What do you think is the risk of an average Dutch person to get colon cancer during their lifetime?   *Of every 100 persons in the Netherlands on average … get colon cancer during their lifetime.*   1. 1 to 2 persons 2. 4 to 5 persons 3. 9 to 10 persons 4. 14 to 15 persons 5. If the stool test shows positive for blood, does this 100% certain mean that someone has colon cancer? 6. Yes 7. No 8. If the stool test shows negative for blood, does this 100% certain mean that someone does not have colon cancer? 9. Yes 10. No |
| --- |

**Appendix B: Overview of mean ranking scores regarding the ranking support question**

| **Rank the following options in order of importance for the government to spent money on** | **Mean ranking score**  *[higher score means ranked as more important]* |
| --- | --- |
| - CRC screening programme | 3.22 |
| - Improving treatment of CRC | 3.16 |
| - Increasing research into causes of CRC | 3.06 |
| - Educating the public on symptoms and risk factors of CRC and what preventive measures they can take themselves | 2.91 |
| - Improving CRC diagnostic tests | 2.65 |
