# Supplementary material for: The Dutch public are positive about the colorectal cancer-screening programme, but is this a well-informed opinion?
Source: BMC Public Health. 2016 Nov 29;16:1208. doi: 10.1186/s12889-016-3870-7 (PMC5129673; doi:10.1186/s12889-016-3870-7)
Supplement: Additional file 2: — Multinomial regression analyses ranking support question_spss output. (DOCX 326 kb) [file 12889_2016_3870_MOESM2_ESM.docx]

**Nominal Regression**

| **Notes** | | |
| --- | --- | --- |
| Output Created | | 22-JAN-2016 12:38:53 |
| Comments | |  |
| Input | Data | \\fs01-home.rivm.nl\home\doumal\Documents\RIVM_VUmc\Publieke opinie - deel A\Analyse vragenlijst 1 deel A\SPSS analyse publieke opinie vragenlijst 1\Resultaten publieke opinie vragenlijst 1_werkbestand 1.sav |
|  | Active Dataset | DataSet1 |
|  | Filter | <none> |
|  | Weight | <none> |
|  | Split File | <none> |
|  | N of Rows in Working Data File | 1679 |
| Missing Value Handling | Definition of Missing | User-defined missing values are treated as missing. |
|  | Cases Used | Statistics are based on all cases with valid data for all variables in the model. |
| Syntax | | NOMREG ANSWER.A019.01.01 (BASE=FIRST ORDER=ASCENDING) BY geslacht cbs_opleidingsniveau WITH leeftijd  /CRITERIA CIN(95) DELTA(0) MXITER(100) MXSTEP(5) CHKSEP(20) LCONVERGE(0) PCONVERGE(0.000001)  SINGULAR(0.00000001)  /MODEL  /STEPWISE=PIN(.05) POUT(0.1) MINEFFECT(0) RULE(SINGLE) ENTRYMETHOD(LR) REMOVALMETHOD(LR)  /INTERCEPT=INCLUDE  /PRINT=PARAMETER SUMMARY LRT CPS STEP MFI. |
| Resources | Processor Time | 00:00:00,11 |
|  | Elapsed Time | 00:00:00,17 |

| **Case Processing Summary** | | | |
| --- | --- | --- | --- |
|  | | N | Marginal Percentage |
| 23. Als u zou mogen kiezen, waar zou u dan (als eerst) het geld aan besteden? Zet de volgende onderwerpen op volgorde van belangrijkheid. Nr. 1 | Het verbeteren van de behandeling van darmkanker | 390 | 23,2% |
|  | Mensen aanbieden om zich preventief te laten onderzoeken op darmkanker (dit is het bevolkingsonderzoek darmkanker) | 480 | 28,6% |
|  | Meer onderzoek doen naar de oorzaken van darmkanker | 315 | 18,8% |
|  | Het geven van voorlichting over de klachten en risicofactoren en over wat mensen zelf kunnen doen | 358 | 21,3% |
|  | Het verbeteren van testen/onderzoeken om vast te stellen of iemand darmkanker heeft | 136 | 8,1% |
| geslacht | Man | 903 | 53,8% |
|  | Vrouw | 776 | 46,2% |
| cbs_opleidingsniveau | laag | 544 | 32,4% |
|  | mid | 681 | 40,6% |
|  | hoog | 454 | 27,0% |
| Valid | | 1679 | 100,0% |
| Missing | | 0 |  |
| Total | | 1679 |  |
| Subpopulation | | 344^a^ |  |
| a. The dependent variable has only one value observed in 71 (20,6%) subpopulations. | | | |

| **Model Fitting Information** | | | | |
| --- | --- | --- | --- | --- |
| Model | Model Fitting Criteria | Likelihood Ratio Tests | | |
|  | -2 Log Likelihood | Chi-Square | df | Sig. |
| Intercept Only | 2638,500 |  |  |  |
| Final | 2605,289 | 33,211 | 16 | ,007 |

| **Pseudo R-Square** | |
| --- | --- |
| Cox and Snell | ,020 |
| Nagelkerke | ,021 |
| McFadden | ,006 |

| **Likelihood Ratio Tests** | | | | |
| --- | --- | --- | --- | --- |
| Effect | Model Fitting Criteria | Likelihood Ratio Tests | | |
|  | -2 Log Likelihood of Reduced Model | Chi-Square | df | Sig. |
| Intercept | 2605,289^a^ | ,000 | 0 | . |
| leeftijd | 2617,271 | 11,982 | 4 | ,017 |
| geslacht | 2608,212 | 2,924 | 4 | ,571 |
| cbs_opleidingsniveau | 2625,058 | 19,770 | 8 | ,011 |
| The chi-square statistic is the difference in -2 log-likelihoods between the final model and a reduced model. The reduced model is formed by omitting an effect from the final model. The null hypothesis is that all parameters of that effect are 0. | | | | |
| a. This reduced model is equivalent to the final model because omitting the effect does not increase the degrees of freedom. | | | | |

| **Parameter Estimates** | | | | | | | | | |
| --- | --- | --- | --- | --- | --- | --- | --- | --- | --- |
| 23. Als u zou mogen kiezen, waar zou u dan (als eerst) het geld aan besteden? Zet de volgende onderwerpen op volgorde van belangrijkheid. Nr. 1^a^ | | B | Std. Error | Wald | df | Sig. | Exp(B) | 95% Confidence Interval for Exp(B) | |
|  |  |  |  |  |  |  |  | Lower Bound | Upper Bound |
| Mensen aanbieden om zich preventief te laten onderzoeken op darmkanker (dit is het bevolkingsonderzoek darmkanker) | Intercept | -,577 | ,243 | 5,626 | 1 | ,018 |  |  |  |
|  | leeftijd | ,013 | ,005 | 8,543 | 1 | ,003 | 1,013 | 1,004 | 1,023 |
|  | [geslacht=1] | -,033 | ,141 | ,055 | 1 | ,815 | ,968 | ,734 | 1,275 |
|  | [geslacht=2] | 0^b^ | . | . | 0 | . | . | . | . |
|  | [cbs_opleidingsniveau=1,00] | ,128 | ,187 | ,469 | 1 | ,493 | 1,137 | ,787 | 1,642 |
|  | [cbs_opleidingsniveau=2,00] | ,238 | ,173 | 1,902 | 1 | ,168 | 1,269 | ,904 | 1,781 |
|  | [cbs_opleidingsniveau=3,00] | 0^b^ | . | . | 0 | . | . | . | . |
| Meer onderzoek doen naar de oorzaken van darmkanker | Intercept | -,785 | ,266 | 8,724 | 1 | ,003 |  |  |  |
|  | leeftijd | ,013 | ,005 | 6,578 | 1 | ,010 | 1,013 | 1,003 | 1,023 |
|  | [geslacht=1] | ,013 | ,157 | ,007 | 1 | ,934 | 1,013 | ,745 | 1,378 |
|  | [geslacht=2] | 0^b^ | . | . | 0 | . | . | . | . |
|  | [cbs_opleidingsniveau=1,00] | -,344 | ,209 | 2,712 | 1 | ,100 | ,709 | ,471 | 1,068 |
|  | [cbs_opleidingsniveau=2,00] | ,068 | ,185 | ,135 | 1 | ,713 | 1,070 | ,745 | 1,538 |
|  | [cbs_opleidingsniveau=3,00] | 0^b^ | . | . | 0 | . | . | . | . |
| Het geven van voorlichting over de klachten en risicofactoren en over wat mensen zelf kunnen doen | Intercept | -,528 | ,255 | 4,271 | 1 | ,039 |  |  |  |
|  | leeftijd | ,014 | ,005 | 7,557 | 1 | ,006 | 1,014 | 1,004 | 1,023 |
|  | [geslacht=1] | -,166 | ,151 | 1,206 | 1 | ,272 | ,847 | ,630 | 1,139 |
|  | [geslacht=2] | 0^b^ | . | . | 0 | . | . | . | . |
|  | [cbs_opleidingsniveau=1,00] | -,291 | ,198 | 2,159 | 1 | ,142 | ,748 | ,507 | 1,102 |
|  | [cbs_opleidingsniveau=2,00] | -,114 | ,180 | ,406 | 1 | ,524 | ,892 | ,627 | 1,268 |
|  | [cbs_opleidingsniveau=3,00] | 0^b^ | . | . | 0 | . | . | . | . |
| Het verbeteren van testen/onderzoeken om vast te stellen of iemand darmkanker heeft | Intercept | -1,372 | ,354 | 14,989 | 1 | ,000 |  |  |  |
|  | leeftijd | ,006 | ,007 | ,803 | 1 | ,370 | 1,006 | ,993 | 1,019 |
|  | [geslacht=1] | -,252 | ,204 | 1,514 | 1 | ,219 | ,778 | ,521 | 1,161 |
|  | [geslacht=2] | 0^b^ | . | . | 0 | . | . | . | . |
|  | [cbs_opleidingsniveau=1,00] | ,424 | ,269 | 2,490 | 1 | ,115 | 1,528 | ,902 | 2,588 |
|  | [cbs_opleidingsniveau=2,00] | -,019 | ,264 | ,005 | 1 | ,944 | ,981 | ,585 | 1,646 |
|  | [cbs_opleidingsniveau=3,00] | 0^b^ | . | . | 0 | . | . | . | . |
| a. The reference category is: Het verbeteren van de behandeling van darmkanker. | | | | | | | | | |
| b. This parameter is set to zero because it is redundant. | | | | | | | | | |

NOMREG ANSWER.A019.01.02 (BASE=FIRST ORDER=ASCENDING) BY geslacht cbs_opleidingsniveau WITH leeftijd

/CRITERIA CIN(95) DELTA(0) MXITER(100) MXSTEP(5) CHKSEP(20) LCONVERGE(0) PCONVERGE(0.000001)

SINGULAR(0.00000001)

/MODEL

/STEPWISE=PIN(.05) POUT(0.1) MINEFFECT(0) RULE(SINGLE) ENTRYMETHOD(LR) REMOVALMETHOD(LR)

/INTERCEPT=INCLUDE

/PRINT=PARAMETER SUMMARY LRT CPS STEP MFI.

**Nominal Regression**

| **Notes** | | |
| --- | --- | --- |
| Output Created | | 22-JAN-2016 12:43:36 |
| Comments | |  |
| Input | Data | \\fs01-home.rivm.nl\home\doumal\Documents\RIVM_VUmc\Publieke opinie - deel A\Analyse vragenlijst 1 deel A\SPSS analyse publieke opinie vragenlijst 1\Resultaten publieke opinie vragenlijst 1_werkbestand 1.sav |
|  | Active Dataset | DataSet1 |
|  | Filter | <none> |
|  | Weight | <none> |
|  | Split File | <none> |
|  | N of Rows in Working Data File | 1679 |
| Missing Value Handling | Definition of Missing | User-defined missing values are treated as missing. |
|  | Cases Used | Statistics are based on all cases with valid data for all variables in the model. |
| Syntax | | NOMREG ANSWER.A019.01.02 (BASE=FIRST ORDER=ASCENDING) BY geslacht cbs_opleidingsniveau WITH leeftijd  /CRITERIA CIN(95) DELTA(0) MXITER(100) MXSTEP(5) CHKSEP(20) LCONVERGE(0) PCONVERGE(0.000001)  SINGULAR(0.00000001)  /MODEL  /STEPWISE=PIN(.05) POUT(0.1) MINEFFECT(0) RULE(SINGLE) ENTRYMETHOD(LR) REMOVALMETHOD(LR)  /INTERCEPT=INCLUDE  /PRINT=PARAMETER SUMMARY LRT CPS STEP MFI. |
| Resources | Processor Time | 00:00:00,09 |
|  | Elapsed Time | 00:00:00,13 |

| **Case Processing Summary** | | | |
| --- | --- | --- | --- |
|  | | N | Marginal Percentage |
| 23. Als u zou mogen kiezen, waar zou u dan (als eerst) het geld aan besteden? Zet de volgende onderwerpen op volgorde van belangrijkheid. Nr. 2 | Het verbeteren van de behandeling van darmkanker | 349 | 20,8% |
|  | Mensen aanbieden om zich preventief te laten onderzoeken op darmkanker (dit is het bevolkingsonderzoek darmkanker) | 324 | 19,3% |
|  | Meer onderzoek doen naar de oorzaken van darmkanker | 364 | 21,7% |
|  | Het geven van voorlichting over de klachten en risicofactoren en over wat mensen zelf kunnen doen | 318 | 18,9% |
|  | Het verbeteren van testen/onderzoeken om vast te stellen of iemand darmkanker heeft | 324 | 19,3% |
| geslacht | Man | 903 | 53,8% |
|  | Vrouw | 776 | 46,2% |
| cbs_opleidingsniveau | laag | 544 | 32,4% |
|  | mid | 681 | 40,6% |
|  | hoog | 454 | 27,0% |
| Valid | | 1679 | 100,0% |
| Missing | | 0 |  |
| Total | | 1679 |  |
| Subpopulation | | 344^a^ |  |
| a. The dependent variable has only one value observed in 66 (19,2%) subpopulations. | | | |

| **Model Fitting Information** | | | | |
| --- | --- | --- | --- | --- |
| Model | Model Fitting Criteria | Likelihood Ratio Tests | | |
|  | -2 Log Likelihood | Chi-Square | df | Sig. |
| Intercept Only | 2771,756 |  |  |  |
| Final | 2755,567 | 16,189 | 16 | ,440 |

| **Pseudo R-Square** | |
| --- | --- |
| Cox and Snell | ,010 |
| Nagelkerke | ,010 |
| McFadden | ,003 |

| **Likelihood Ratio Tests** | | | | |
| --- | --- | --- | --- | --- |
| Effect | Model Fitting Criteria | Likelihood Ratio Tests | | |
|  | -2 Log Likelihood of Reduced Model | Chi-Square | df | Sig. |
| Intercept | 2755,567^a^ | ,000 | 0 | . |
| leeftijd | 2756,722 | 1,155 | 4 | ,885 |
| geslacht | 2763,790 | 8,222 | 4 | ,084 |
| cbs_opleidingsniveau | 2762,018 | 6,451 | 8 | ,597 |
| The chi-square statistic is the difference in -2 log-likelihoods between the final model and a reduced model. The reduced model is formed by omitting an effect from the final model. The null hypothesis is that all parameters of that effect are 0. | | | | |
| a. This reduced model is equivalent to the final model because omitting the effect does not increase the degrees of freedom. | | | | |

| **Parameter Estimates** | | | | | | | | | |
| --- | --- | --- | --- | --- | --- | --- | --- | --- | --- |
| 23. Als u zou mogen kiezen, waar zou u dan (als eerst) het geld aan besteden? Zet de volgende onderwerpen op volgorde van belangrijkheid. Nr. 2^a^ | | B | Std. Error | Wald | df | Sig. | Exp(B) | 95% Confidence Interval for Exp(B) | |
|  |  |  |  |  |  |  |  | Lower Bound | Upper Bound |
| Mensen aanbieden om zich preventief te laten onderzoeken op darmkanker (dit is het bevolkingsonderzoek darmkanker) | Intercept | -,061 | ,271 | ,050 | 1 | ,823 |  |  |  |
|  | leeftijd | ,005 | ,005 | ,947 | 1 | ,330 | 1,005 | ,995 | 1,015 |
|  | [geslacht=1] | -,359 | ,160 | 5,068 | 1 | ,024 | ,698 | ,511 | ,955 |
|  | [geslacht=2] | 0^b^ | . | . | 0 | . | . | . | . |
|  | [cbs_opleidingsniveau=1,00] | -,026 | ,213 | ,015 | 1 | ,901 | ,974 | ,641 | 1,479 |
|  | [cbs_opleidingsniveau=2,00] | -,163 | ,190 | ,736 | 1 | ,391 | ,850 | ,586 | 1,232 |
|  | [cbs_opleidingsniveau=3,00] | 0^b^ | . | . | 0 | . | . | . | . |
| Meer onderzoek doen naar de oorzaken van darmkanker | Intercept | -,199 | ,268 | ,555 | 1 | ,456 |  |  |  |
|  | leeftijd | ,003 | ,005 | ,401 | 1 | ,526 | 1,003 | ,993 | 1,013 |
|  | [geslacht=1] | ,024 | ,155 | ,023 | 1 | ,879 | 1,024 | ,755 | 1,388 |
|  | [geslacht=2] | 0^b^ | . | . | 0 | . | . | . | . |
|  | [cbs_opleidingsniveau=1,00] | ,276 | ,206 | 1,783 | 1 | ,182 | 1,317 | ,879 | 1,974 |
|  | [cbs_opleidingsniveau=2,00] | -,044 | ,188 | ,054 | 1 | ,817 | ,957 | ,662 | 1,384 |
|  | [cbs_opleidingsniveau=3,00] | 0^b^ | . | . | 0 | . | . | . | . |
| Het geven van voorlichting over de klachten en risicofactoren en over wat mensen zelf kunnen doen | Intercept | -,121 | ,273 | ,198 | 1 | ,657 |  |  |  |
|  | leeftijd | ,004 | ,005 | ,732 | 1 | ,392 | 1,004 | ,994 | 1,015 |
|  | [geslacht=1] | -,194 | ,160 | 1,458 | 1 | ,227 | ,824 | ,602 | 1,128 |
|  | [geslacht=2] | 0^b^ | . | . | 0 | . | . | . | . |
|  | [cbs_opleidingsniveau=1,00] | -,040 | ,213 | ,035 | 1 | ,853 | ,961 | ,633 | 1,460 |
|  | [cbs_opleidingsniveau=2,00] | -,179 | ,190 | ,883 | 1 | ,347 | ,836 | ,576 | 1,214 |
|  | [cbs_opleidingsniveau=3,00] | 0^b^ | . | . | 0 | . | . | . | . |
| Het verbeteren van testen/onderzoeken om vast te stellen of iemand darmkanker heeft | Intercept | -,254 | ,275 | ,856 | 1 | ,355 |  |  |  |
|  | leeftijd | ,003 | ,005 | ,417 | 1 | ,519 | 1,003 | ,993 | 1,013 |
|  | [geslacht=1] | -,031 | ,160 | ,038 | 1 | ,846 | ,969 | ,709 | 1,326 |
|  | [geslacht=2] | 0^b^ | . | . | 0 | . | . | . | . |
|  | [cbs_opleidingsniveau=1,00] | ,224 | ,212 | 1,118 | 1 | ,290 | 1,251 | ,826 | 1,894 |
|  | [cbs_opleidingsniveau=2,00] | -,096 | ,193 | ,246 | 1 | ,620 | ,909 | ,622 | 1,327 |
|  | [cbs_opleidingsniveau=3,00] | 0^b^ | . | . | 0 | . | . | . | . |
| a. The reference category is: Het verbeteren van de behandeling van darmkanker. | | | | | | | | | |
| b. This parameter is set to zero because it is redundant. | | | | | | | | | |

NOMREG ANSWER.A019.01.03 (BASE=FIRST ORDER=ASCENDING) BY geslacht cbs_opleidingsniveau WITH leeftijd

/CRITERIA CIN(95) DELTA(0) MXITER(100) MXSTEP(5) CHKSEP(20) LCONVERGE(0) PCONVERGE(0.000001)

SINGULAR(0.00000001)

/MODEL

/STEPWISE=PIN(.05) POUT(0.1) MINEFFECT(0) RULE(SINGLE) ENTRYMETHOD(LR) REMOVALMETHOD(LR)

/INTERCEPT=INCLUDE

/PRINT=PARAMETER SUMMARY LRT CPS STEP MFI.

**Nominal Regression**

| **Notes** | | |
| --- | --- | --- |
| Output Created | | 22-JAN-2016 12:45:26 |
| Comments | |  |
| Input | Data | \\fs01-home.rivm.nl\home\doumal\Documents\RIVM_VUmc\Publieke opinie - deel A\Analyse vragenlijst 1 deel A\SPSS analyse publieke opinie vragenlijst 1\Resultaten publieke opinie vragenlijst 1_werkbestand 1.sav |
|  | Active Dataset | DataSet1 |
|  | Filter | <none> |
|  | Weight | <none> |
|  | Split File | <none> |
|  | N of Rows in Working Data File | 1679 |
| Missing Value Handling | Definition of Missing | User-defined missing values are treated as missing. |
|  | Cases Used | Statistics are based on all cases with valid data for all variables in the model. |
| Syntax | | NOMREG ANSWER.A019.01.03 (BASE=FIRST ORDER=ASCENDING) BY geslacht cbs_opleidingsniveau WITH leeftijd  /CRITERIA CIN(95) DELTA(0) MXITER(100) MXSTEP(5) CHKSEP(20) LCONVERGE(0) PCONVERGE(0.000001)  SINGULAR(0.00000001)  /MODEL  /STEPWISE=PIN(.05) POUT(0.1) MINEFFECT(0) RULE(SINGLE) ENTRYMETHOD(LR) REMOVALMETHOD(LR)  /INTERCEPT=INCLUDE  /PRINT=PARAMETER SUMMARY LRT CPS STEP MFI. |
| Resources | Processor Time | 00:00:00,12 |
|  | Elapsed Time | 00:00:00,13 |

| **Case Processing Summary** | | | |
| --- | --- | --- | --- |
|  | | N | Marginal Percentage |
| 23. Als u zou mogen kiezen, waar zou u dan (als eerst) het geld aan besteden? Zet de volgende onderwerpen op volgorde van belangrijkheid. Nr. 3 | Het verbeteren van de behandeling van darmkanker | 354 | 21,1% |
|  | Mensen aanbieden om zich preventief te laten onderzoeken op darmkanker (dit is het bevolkingsonderzoek darmkanker) | 283 | 16,9% |
|  | Meer onderzoek doen naar de oorzaken van darmkanker | 367 | 21,9% |
|  | Het geven van voorlichting over de klachten en risicofactoren en over wat mensen zelf kunnen doen | 255 | 15,2% |
|  | Het verbeteren van testen/onderzoeken om vast te stellen of iemand darmkanker heeft | 420 | 25,0% |
| geslacht | Man | 903 | 53,8% |
|  | Vrouw | 776 | 46,2% |
| cbs_opleidingsniveau | laag | 544 | 32,4% |
|  | mid | 681 | 40,6% |
|  | hoog | 454 | 27,0% |
| Valid | | 1679 | 100,0% |
| Missing | | 0 |  |
| Total | | 1679 |  |
| Subpopulation | | 344^a^ |  |
| a. The dependent variable has only one value observed in 73 (21,2%) subpopulations. | | | |

| **Model Fitting Information** | | | | |
| --- | --- | --- | --- | --- |
| Model | Model Fitting Criteria | Likelihood Ratio Tests | | |
|  | -2 Log Likelihood | Chi-Square | df | Sig. |
| Intercept Only | 2754,662 |  |  |  |
| Final | 2745,101 | 9,561 | 16 | ,889 |

| **Pseudo R-Square** | |
| --- | --- |
| Cox and Snell | ,006 |
| Nagelkerke | ,006 |
| McFadden | ,002 |

| **Likelihood Ratio Tests** | | | | |
| --- | --- | --- | --- | --- |
| Effect | Model Fitting Criteria | Likelihood Ratio Tests | | |
|  | -2 Log Likelihood of Reduced Model | Chi-Square | df | Sig. |
| Intercept | 2745,101^a^ | ,000 | 0 | . |
| leeftijd | 2746,223 | 1,122 | 4 | ,891 |
| geslacht | 2749,301 | 4,200 | 4 | ,380 |
| cbs_opleidingsniveau | 2748,839 | 3,738 | 8 | ,880 |
| The chi-square statistic is the difference in -2 log-likelihoods between the final model and a reduced model. The reduced model is formed by omitting an effect from the final model. The null hypothesis is that all parameters of that effect are 0. | | | | |
| a. This reduced model is equivalent to the final model because omitting the effect does not increase the degrees of freedom. | | | | |

| **Parameter Estimates** | | | | | | | | | |
| --- | --- | --- | --- | --- | --- | --- | --- | --- | --- |
| 23. Als u zou mogen kiezen, waar zou u dan (als eerst) het geld aan besteden? Zet de volgende onderwerpen op volgorde van belangrijkheid. Nr. 3^a^ | | B | Std. Error | Wald | df | Sig. | Exp(B) | 95% Confidence Interval for Exp(B) | |
|  |  |  |  |  |  |  |  | Lower Bound | Upper Bound |
| Mensen aanbieden om zich preventief te laten onderzoeken op darmkanker (dit is het bevolkingsonderzoek darmkanker) | Intercept | ,038 | ,281 | ,018 | 1 | ,893 |  |  |  |
|  | leeftijd | -,005 | ,005 | ,937 | 1 | ,333 | ,995 | ,984 | 1,005 |
|  | [geslacht=1] | ,143 | ,164 | ,759 | 1 | ,384 | 1,154 | ,836 | 1,593 |
|  | [geslacht=2] | 0^b^ | . | . | 0 | . | . | . | . |
|  | [cbs_opleidingsniveau=1,00] | -,189 | ,218 | ,751 | 1 | ,386 | ,828 | ,540 | 1,269 |
|  | [cbs_opleidingsniveau=2,00] | -,048 | ,198 | ,058 | 1 | ,810 | ,953 | ,647 | 1,405 |
|  | [cbs_opleidingsniveau=3,00] | 0^b^ | . | . | 0 | . | . | . | . |
| Meer onderzoek doen naar de oorzaken van darmkanker | Intercept | -,004 | ,268 | ,000 | 1 | ,988 |  |  |  |
|  | leeftijd | -,001 | ,005 | ,057 | 1 | ,812 | ,999 | ,989 | 1,009 |
|  | [geslacht=1] | ,125 | ,153 | ,661 | 1 | ,416 | 1,133 | ,839 | 1,530 |
|  | [geslacht=2] | 0^b^ | . | . | 0 | . | . | . | . |
|  | [cbs_opleidingsniveau=1,00] | ,035 | ,204 | ,030 | 1 | ,863 | 1,036 | ,695 | 1,545 |
|  | [cbs_opleidingsniveau=2,00] | ,057 | ,189 | ,092 | 1 | ,762 | 1,059 | ,731 | 1,534 |
|  | [cbs_opleidingsniveau=3,00] | 0^b^ | . | . | 0 | . | . | . | . |
| Het geven van voorlichting over de klachten en risicofactoren en over wat mensen zelf kunnen doen | Intercept | -,231 | ,292 | ,627 | 1 | ,428 |  |  |  |
|  | leeftijd | ,000 | ,006 | ,007 | 1 | ,933 | 1,000 | ,989 | 1,010 |
|  | [geslacht=1] | ,064 | ,169 | ,141 | 1 | ,707 | 1,066 | ,765 | 1,485 |
|  | [geslacht=2] | 0^b^ | . | . | 0 | . | . | . | . |
|  | [cbs_opleidingsniveau=1,00] | -,118 | ,221 | ,288 | 1 | ,592 | ,888 | ,576 | 1,369 |
|  | [cbs_opleidingsniveau=2,00] | -,171 | ,206 | ,688 | 1 | ,407 | ,843 | ,563 | 1,262 |
|  | [cbs_opleidingsniveau=3,00] | 0^b^ | . | . | 0 | . | . | . | . |
| Het verbeteren van testen/onderzoeken om vast te stellen of iemand darmkanker heeft | Intercept | ,140 | ,258 | ,295 | 1 | ,587 |  |  |  |
|  | leeftijd | -,002 | ,005 | ,211 | 1 | ,646 | ,998 | ,988 | 1,007 |
|  | [geslacht=1] | ,291 | ,149 | 3,790 | 1 | ,052 | 1,337 | ,998 | 1,792 |
|  | [geslacht=2] | 0^b^ | . | . | 0 | . | . | . | . |
|  | [cbs_opleidingsniveau=1,00] | -,125 | ,198 | ,401 | 1 | ,526 | ,882 | ,598 | 1,300 |
|  | [cbs_opleidingsniveau=2,00] | ,061 | ,181 | ,114 | 1 | ,735 | 1,063 | ,745 | 1,516 |
|  | [cbs_opleidingsniveau=3,00] | 0^b^ | . | . | 0 | . | . | . | . |
| a. The reference category is: Het verbeteren van de behandeling van darmkanker. | | | | | | | | | |
| b. This parameter is set to zero because it is redundant. | | | | | | | | | |

NOMREG ANSWER.A019.01.04 (BASE=FIRST ORDER=ASCENDING) BY geslacht cbs_opleidingsniveau WITH leeftijd

/CRITERIA CIN(95) DELTA(0) MXITER(100) MXSTEP(5) CHKSEP(20) LCONVERGE(0) PCONVERGE(0.000001)

SINGULAR(0.00000001)

/MODEL

/STEPWISE=PIN(.05) POUT(0.1) MINEFFECT(0) RULE(SINGLE) ENTRYMETHOD(LR) REMOVALMETHOD(LR)

/INTERCEPT=INCLUDE

/PRINT=PARAMETER SUMMARY LRT CPS STEP MFI.

**Nominal Regression**

| **Notes** | | |
| --- | --- | --- |
| Output Created | | 22-JAN-2016 12:47:07 |
| Comments | |  |
| Input | Data | \\fs01-home.rivm.nl\home\doumal\Documents\RIVM_VUmc\Publieke opinie - deel A\Analyse vragenlijst 1 deel A\SPSS analyse publieke opinie vragenlijst 1\Resultaten publieke opinie vragenlijst 1_werkbestand 1.sav |
|  | Active Dataset | DataSet1 |
|  | Filter | <none> |
|  | Weight | <none> |
|  | Split File | <none> |
|  | N of Rows in Working Data File | 1679 |
| Missing Value Handling | Definition of Missing | User-defined missing values are treated as missing. |
|  | Cases Used | Statistics are based on all cases with valid data for all variables in the model. |
| Syntax | | NOMREG ANSWER.A019.01.04 (BASE=FIRST ORDER=ASCENDING) BY geslacht cbs_opleidingsniveau WITH leeftijd  /CRITERIA CIN(95) DELTA(0) MXITER(100) MXSTEP(5) CHKSEP(20) LCONVERGE(0) PCONVERGE(0.000001)  SINGULAR(0.00000001)  /MODEL  /STEPWISE=PIN(.05) POUT(0.1) MINEFFECT(0) RULE(SINGLE) ENTRYMETHOD(LR) REMOVALMETHOD(LR)  /INTERCEPT=INCLUDE  /PRINT=PARAMETER SUMMARY LRT CPS STEP MFI. |
| Resources | Processor Time | 00:00:00,12 |
|  | Elapsed Time | 00:00:00,14 |

| **Case Processing Summary** | | | |
| --- | --- | --- | --- |
|  | | N | Marginal Percentage |
| 23. Als u zou mogen kiezen, waar zou u dan (als eerst) het geld aan besteden? Zet de volgende onderwerpen op volgorde van belangrijkheid. Nr. 4 | Het verbeteren van de behandeling van darmkanker | 313 | 18,6% |
|  | Mensen aanbieden om zich preventief te laten onderzoeken op darmkanker (dit is het bevolkingsonderzoek darmkanker) | 277 | 16,5% |
|  | Meer onderzoek doen naar de oorzaken van darmkanker | 374 | 22,3% |
|  | Het geven van voorlichting over de klachten en risicofactoren en over wat mensen zelf kunnen doen | 309 | 18,4% |
|  | Het verbeteren van testen/onderzoeken om vast te stellen of iemand darmkanker heeft | 406 | 24,2% |
| geslacht | Man | 903 | 53,8% |
|  | Vrouw | 776 | 46,2% |
| cbs_opleidingsniveau | laag | 544 | 32,4% |
|  | mid | 681 | 40,6% |
|  | hoog | 454 | 27,0% |
| Valid | | 1679 | 100,0% |
| Missing | | 0 |  |
| Total | | 1679 |  |
| Subpopulation | | 344^a^ |  |
| a. The dependent variable has only one value observed in 69 (20,1%) subpopulations. | | | |

| **Model Fitting Information** | | | | |
| --- | --- | --- | --- | --- |
| Model | Model Fitting Criteria | Likelihood Ratio Tests | | |
|  | -2 Log Likelihood | Chi-Square | df | Sig. |
| Intercept Only | 2760,801 |  |  |  |
| Final | 2734,282 | 26,520 | 16 | ,047 |

| **Pseudo R-Square** | |
| --- | --- |
| Cox and Snell | ,016 |
| Nagelkerke | ,016 |
| McFadden | ,005 |

| **Likelihood Ratio Tests** | | | | |
| --- | --- | --- | --- | --- |
| Effect | Model Fitting Criteria | Likelihood Ratio Tests | | |
|  | -2 Log Likelihood of Reduced Model | Chi-Square | df | Sig. |
| Intercept | 2734,282^a^ | ,000 | 0 | . |
| leeftijd | 2737,022 | 2,741 | 4 | ,602 |
| geslacht | 2744,588 | 10,306 | 4 | ,036 |
| cbs_opleidingsniveau | 2745,193 | 10,912 | 8 | ,207 |
| The chi-square statistic is the difference in -2 log-likelihoods between the final model and a reduced model. The reduced model is formed by omitting an effect from the final model. The null hypothesis is that all parameters of that effect are 0. | | | | |
| a. This reduced model is equivalent to the final model because omitting the effect does not increase the degrees of freedom. | | | | |

| **Parameter Estimates** | | | | | | | | | |
| --- | --- | --- | --- | --- | --- | --- | --- | --- | --- |
| 23. Als u zou mogen kiezen, waar zou u dan (als eerst) het geld aan besteden? Zet de volgende onderwerpen op volgorde van belangrijkheid. Nr. 4^a^ | | B | Std. Error | Wald | df | Sig. | Exp(B) | 95% Confidence Interval for Exp(B) | |
|  |  |  |  |  |  |  |  | Lower Bound | Upper Bound |
| Mensen aanbieden om zich preventief te laten onderzoeken op darmkanker (dit is het bevolkingsonderzoek darmkanker) | Intercept | ,153 | ,301 | ,258 | 1 | ,611 |  |  |  |
|  | leeftijd | -,005 | ,006 | ,919 | 1 | ,338 | ,995 | ,984 | 1,006 |
|  | [geslacht=1] | ,380 | ,172 | 4,897 | 1 | ,027 | 1,463 | 1,044 | 2,049 |
|  | [geslacht=2] | 0^b^ | . | . | 0 | . | . | . | . |
|  | [cbs_opleidingsniveau=1,00] | -,094 | ,226 | ,173 | 1 | ,677 | ,910 | ,584 | 1,418 |
|  | [cbs_opleidingsniveau=2,00] | -,464 | ,212 | 4,804 | 1 | ,028 | ,628 | ,415 | ,952 |
|  | [cbs_opleidingsniveau=3,00] | 0^b^ | . | . | 0 | . | . | . | . |
| Meer onderzoek doen naar de oorzaken van darmkanker | Intercept | ,780 | ,276 | 7,970 | 1 | ,005 |  |  |  |
|  | leeftijd | -,005 | ,005 | 1,033 | 1 | ,310 | ,995 | ,985 | 1,005 |
|  | [geslacht=1] | -,093 | ,158 | ,346 | 1 | ,556 | ,911 | ,668 | 1,242 |
|  | [geslacht=2] | 0^b^ | . | . | 0 | . | . | . | . |
|  | [cbs_opleidingsniveau=1,00] | -,335 | ,215 | 2,440 | 1 | ,118 | ,715 | ,470 | 1,089 |
|  | [cbs_opleidingsniveau=2,00] | -,439 | ,194 | 5,146 | 1 | ,023 | ,644 | ,441 | ,942 |
|  | [cbs_opleidingsniveau=3,00] | 0^b^ | . | . | 0 | . | . | . | . |
| Het geven van voorlichting over de klachten en risicofactoren en over wat mensen zelf kunnen doen | Intercept | -,026 | ,297 | ,008 | 1 | ,929 |  |  |  |
|  | leeftijd | -,001 | ,005 | ,025 | 1 | ,875 | ,999 | ,989 | 1,010 |
|  | [geslacht=1] | ,233 | ,166 | 1,979 | 1 | ,159 | 1,263 | ,912 | 1,748 |
|  | [geslacht=2] | 0^b^ | . | . | 0 | . | . | . | . |
|  | [cbs_opleidingsniveau=1,00] | -,040 | ,227 | ,031 | 1 | ,860 | ,961 | ,616 | 1,499 |
|  | [cbs_opleidingsniveau=2,00] | -,130 | ,208 | ,390 | 1 | ,532 | ,878 | ,585 | 1,320 |
|  | [cbs_opleidingsniveau=3,00] | 0^b^ | . | . | 0 | . | . | . | . |
| Het verbeteren van testen/onderzoeken om vast te stellen of iemand darmkanker heeft | Intercept | ,803 | ,272 | 8,722 | 1 | ,003 |  |  |  |
|  | leeftijd | -,007 | ,005 | 1,823 | 1 | ,177 | ,993 | ,983 | 1,003 |
|  | [geslacht=1] | ,090 | ,155 | ,333 | 1 | ,564 | 1,094 | ,807 | 1,483 |
|  | [geslacht=2] | 0^b^ | . | . | 0 | . | . | . | . |
|  | [cbs_opleidingsniveau=1,00] | -,252 | ,211 | 1,431 | 1 | ,232 | ,777 | ,514 | 1,175 |
|  | [cbs_opleidingsniveau=2,00] | -,396 | ,191 | 4,294 | 1 | ,038 | ,673 | ,463 | ,979 |
|  | [cbs_opleidingsniveau=3,00] | 0^b^ | . | . | 0 | . | . | . | . |
| a. The reference category is: Het verbeteren van de behandeling van darmkanker. | | | | | | | | | |
| b. This parameter is set to zero because it is redundant. | | | | | | | | | |

NOMREG ANSWER.A019.01.05 (BASE=FIRST ORDER=ASCENDING) BY geslacht cbs_opleidingsniveau WITH leeftijd

/CRITERIA CIN(95) DELTA(0) MXITER(100) MXSTEP(5) CHKSEP(20) LCONVERGE(0) PCONVERGE(0.000001)

SINGULAR(0.00000001)

/MODEL

/STEPWISE=PIN(.05) POUT(0.1) MINEFFECT(0) RULE(SINGLE) ENTRYMETHOD(LR) REMOVALMETHOD(LR)

/INTERCEPT=INCLUDE

/PRINT=PARAMETER SUMMARY LRT CPS STEP MFI.

**Nominal Regression**

| **Notes** | | |
| --- | --- | --- |
| Output Created | | 22-JAN-2016 12:51:46 |
| Comments | |  |
| Input | Data | \\fs01-home.rivm.nl\home\doumal\Documents\RIVM_VUmc\Publieke opinie - deel A\Analyse vragenlijst 1 deel A\SPSS analyse publieke opinie vragenlijst 1\Resultaten publieke opinie vragenlijst 1_werkbestand 1.sav |
|  | Active Dataset | DataSet1 |
|  | Filter | <none> |
|  | Weight | <none> |
|  | Split File | <none> |
|  | N of Rows in Working Data File | 1679 |
| Missing Value Handling | Definition of Missing | User-defined missing values are treated as missing. |
|  | Cases Used | Statistics are based on all cases with valid data for all variables in the model. |
| Syntax | | NOMREG ANSWER.A019.01.05 (BASE=FIRST ORDER=ASCENDING) BY geslacht cbs_opleidingsniveau WITH leeftijd  /CRITERIA CIN(95) DELTA(0) MXITER(100) MXSTEP(5) CHKSEP(20) LCONVERGE(0) PCONVERGE(0.000001)  SINGULAR(0.00000001)  /MODEL  /STEPWISE=PIN(.05) POUT(0.1) MINEFFECT(0) RULE(SINGLE) ENTRYMETHOD(LR) REMOVALMETHOD(LR)  /INTERCEPT=INCLUDE  /PRINT=PARAMETER SUMMARY LRT CPS STEP MFI. |
| Resources | Processor Time | 00:00:00,11 |
|  | Elapsed Time | 00:00:00,12 |

| **Case Processing Summary** | | | |
| --- | --- | --- | --- |
|  | | N | Marginal Percentage |
| 23. Als u zou mogen kiezen, waar zou u dan (als eerst) het geld aan besteden? Zet de volgende onderwerpen op volgorde van belangrijkheid. Nr. 5 | Het verbeteren van de behandeling van darmkanker | 273 | 16,3% |
|  | Mensen aanbieden om zich preventief te laten onderzoeken op darmkanker (dit is het bevolkingsonderzoek darmkanker) | 315 | 18,8% |
|  | Meer onderzoek doen naar de oorzaken van darmkanker | 259 | 15,4% |
|  | Het geven van voorlichting over de klachten en risicofactoren en over wat mensen zelf kunnen doen | 439 | 26,1% |
|  | Het verbeteren van testen/onderzoeken om vast te stellen of iemand darmkanker heeft | 393 | 23,4% |
| geslacht | Man | 903 | 53,8% |
|  | Vrouw | 776 | 46,2% |
| cbs_opleidingsniveau | laag | 544 | 32,4% |
|  | mid | 681 | 40,6% |
|  | hoog | 454 | 27,0% |
| Valid | | 1679 | 100,0% |
| Missing | | 0 |  |
| Total | | 1679 |  |
| Subpopulation | | 344^a^ |  |
| a. The dependent variable has only one value observed in 72 (20,9%) subpopulations. | | | |

| **Model Fitting Information** | | | | |
| --- | --- | --- | --- | --- |
| Model | Model Fitting Criteria | Likelihood Ratio Tests | | |
|  | -2 Log Likelihood | Chi-Square | df | Sig. |
| Intercept Only | 2739,562 |  |  |  |
| Final | 2707,744 | 31,818 | 16 | ,011 |

| **Pseudo R-Square** | |
| --- | --- |
| Cox and Snell | ,019 |
| Nagelkerke | ,020 |
| McFadden | ,006 |

| **Likelihood Ratio Tests** | | | | |
| --- | --- | --- | --- | --- |
| Effect | Model Fitting Criteria | Likelihood Ratio Tests | | |
|  | -2 Log Likelihood of Reduced Model | Chi-Square | df | Sig. |
| Intercept | 2707,744^a^ | ,000 | 0 | . |
| leeftijd | 2723,157 | 15,413 | 4 | ,004 |
| geslacht | 2711,038 | 3,293 | 4 | ,510 |
| cbs_opleidingsniveau | 2722,733 | 14,988 | 8 | ,059 |
| The chi-square statistic is the difference in -2 log-likelihoods between the final model and a reduced model. The reduced model is formed by omitting an effect from the final model. The null hypothesis is that all parameters of that effect are 0. | | | | |
| a. This reduced model is equivalent to the final model because omitting the effect does not increase the degrees of freedom. | | | | |

| **Parameter Estimates** | | | | | | | | | |
| --- | --- | --- | --- | --- | --- | --- | --- | --- | --- |
| 23. Als u zou mogen kiezen, waar zou u dan (als eerst) het geld aan besteden? Zet de volgende onderwerpen op volgorde van belangrijkheid. Nr. 5^a^ | | B | Std. Error | Wald | df | Sig. | Exp(B) | 95% Confidence Interval for Exp(B) | |
|  |  |  |  |  |  |  |  | Lower Bound | Upper Bound |
| Mensen aanbieden om zich preventief te laten onderzoeken op darmkanker (dit is het bevolkingsonderzoek darmkanker) | Intercept | ,761 | ,297 | 6,565 | 1 | ,010 |  |  |  |
|  | leeftijd | -,015 | ,006 | 6,776 | 1 | ,009 | ,985 | ,975 | ,996 |
|  | [geslacht=1] | -,091 | ,172 | ,276 | 1 | ,599 | ,913 | ,652 | 1,280 |
|  | [geslacht=2] | 0^b^ | . | . | 0 | . | . | . | . |
|  | [cbs_opleidingsniveau=1,00] | ,109 | ,220 | ,244 | 1 | ,621 | 1,115 | ,724 | 1,716 |
|  | [cbs_opleidingsniveau=2,00] | ,382 | ,205 | 3,468 | 1 | ,063 | 1,465 | ,980 | 2,190 |
|  | [cbs_opleidingsniveau=3,00] | 0^b^ | . | . | 0 | . | . | . | . |
| Meer onderzoek doen naar de oorzaken van darmkanker | Intercept | ,413 | ,314 | 1,724 | 1 | ,189 |  |  |  |
|  | leeftijd | -,014 | ,006 | 6,028 | 1 | ,014 | ,986 | ,974 | ,997 |
|  | [geslacht=1] | -,035 | ,180 | ,038 | 1 | ,845 | ,965 | ,678 | 1,375 |
|  | [geslacht=2] | 0^b^ | . | . | 0 | . | . | . | . |
|  | [cbs_opleidingsniveau=1,00] | ,394 | ,231 | 2,921 | 1 | ,087 | 1,483 | ,944 | 2,331 |
|  | [cbs_opleidingsniveau=2,00] | ,438 | ,220 | 3,965 | 1 | ,046 | 1,549 | 1,007 | 2,384 |
|  | [cbs_opleidingsniveau=3,00] | 0^b^ | . | . | 0 | . | . | . | . |
| Het geven van voorlichting over de klachten en risicofactoren en over wat mensen zelf kunnen doen | Intercept | 1,010 | ,281 | 12,931 | 1 | ,000 |  |  |  |
|  | leeftijd | -,018 | ,005 | 12,207 | 1 | ,000 | ,982 | ,972 | ,992 |
|  | [geslacht=1] | ,033 | ,161 | ,042 | 1 | ,838 | 1,034 | ,754 | 1,418 |
|  | [geslacht=2] | 0^b^ | . | . | 0 | . | . | . | . |
|  | [cbs_opleidingsniveau=1,00] | ,463 | ,206 | 5,051 | 1 | ,025 | 1,589 | 1,061 | 2,378 |
|  | [cbs_opleidingsniveau=2,00] | ,589 | ,196 | 9,059 | 1 | ,003 | 1,802 | 1,228 | 2,645 |
|  | [cbs_opleidingsniveau=3,00] | 0^b^ | . | . | 0 | . | . | . | . |
| Het verbeteren van testen/onderzoeken om vast te stellen of iemand darmkanker heeft | Intercept | ,643 | ,288 | 4,997 | 1 | ,025 |  |  |  |
|  | leeftijd | -,007 | ,005 | 1,649 | 1 | ,199 | ,993 | ,983 | 1,004 |
|  | [geslacht=1] | -,210 | ,164 | 1,633 | 1 | ,201 | ,811 | ,587 | 1,119 |
|  | [geslacht=2] | 0^b^ | . | . | 0 | . | . | . | . |
|  | [cbs_opleidingsniveau=1,00] | ,071 | ,210 | ,116 | 1 | ,734 | 1,074 | ,712 | 1,621 |
|  | [cbs_opleidingsniveau=2,00] | ,447 | ,196 | 5,180 | 1 | ,023 | 1,563 | 1,064 | 2,296 |
|  | [cbs_opleidingsniveau=3,00] | 0^b^ | . | . | 0 | . | . | . | . |
| a. The reference category is: Het verbeteren van de behandeling van darmkanker. | | | | | | | | | |
| b. This parameter is set to zero because it is redundant. | | | | | | | | | |

NOMREG ANSWER.A019.01.01 (BASE=LAST ORDER=ASCENDING) BY geslacht cbs_opleidingsniveau WITH leeftijd

/CRITERIA CIN(95) DELTA(0) MXITER(100) MXSTEP(5) CHKSEP(20) LCONVERGE(0) PCONVERGE(0.000001)

SINGULAR(0.00000001)

/MODEL

/STEPWISE=PIN(.05) POUT(0.1) MINEFFECT(0) RULE(SINGLE) ENTRYMETHOD(LR) REMOVALMETHOD(LR)

/INTERCEPT=INCLUDE

/PRINT=PARAMETER SUMMARY LRT CPS STEP MFI.

**Nominal Regression**

| **Notes** | | |
| --- | --- | --- |
| Output Created | | 22-JAN-2016 13:07:34 |
| Comments | |  |
| Input | Data | \\fs01-home.rivm.nl\home\doumal\Documents\RIVM_VUmc\Publieke opinie - deel A\Analyse vragenlijst 1 deel A\SPSS analyse publieke opinie vragenlijst 1\Resultaten publieke opinie vragenlijst 1_werkbestand 1.sav |
|  | Active Dataset | DataSet1 |
|  | Filter | <none> |
|  | Weight | <none> |
|  | Split File | <none> |
|  | N of Rows in Working Data File | 1679 |
| Missing Value Handling | Definition of Missing | User-defined missing values are treated as missing. |
|  | Cases Used | Statistics are based on all cases with valid data for all variables in the model. |
| Syntax | | NOMREG ANSWER.A019.01.01 (BASE=LAST ORDER=ASCENDING) BY geslacht cbs_opleidingsniveau WITH leeftijd  /CRITERIA CIN(95) DELTA(0) MXITER(100) MXSTEP(5) CHKSEP(20) LCONVERGE(0) PCONVERGE(0.000001)  SINGULAR(0.00000001)  /MODEL  /STEPWISE=PIN(.05) POUT(0.1) MINEFFECT(0) RULE(SINGLE) ENTRYMETHOD(LR) REMOVALMETHOD(LR)  /INTERCEPT=INCLUDE  /PRINT=PARAMETER SUMMARY LRT CPS STEP MFI. |
| Resources | Processor Time | 00:00:00,13 |
|  | Elapsed Time | 00:00:00,12 |

| **Case Processing Summary** | | | |
| --- | --- | --- | --- |
|  | | N | Marginal Percentage |
| 23. Als u zou mogen kiezen, waar zou u dan (als eerst) het geld aan besteden? Zet de volgende onderwerpen op volgorde van belangrijkheid. Nr. 1 | Het verbeteren van de behandeling van darmkanker | 390 | 23,2% |
|  | Mensen aanbieden om zich preventief te laten onderzoeken op darmkanker (dit is het bevolkingsonderzoek darmkanker) | 480 | 28,6% |
|  | Meer onderzoek doen naar de oorzaken van darmkanker | 315 | 18,8% |
|  | Het geven van voorlichting over de klachten en risicofactoren en over wat mensen zelf kunnen doen | 358 | 21,3% |
|  | Het verbeteren van testen/onderzoeken om vast te stellen of iemand darmkanker heeft | 136 | 8,1% |
| geslacht | Man | 903 | 53,8% |
|  | Vrouw | 776 | 46,2% |
| cbs_opleidingsniveau | laag | 544 | 32,4% |
|  | mid | 681 | 40,6% |
|  | hoog | 454 | 27,0% |
| Valid | | 1679 | 100,0% |
| Missing | | 0 |  |
| Total | | 1679 |  |
| Subpopulation | | 344^a^ |  |
| a. The dependent variable has only one value observed in 71 (20,6%) subpopulations. | | | |

| **Model Fitting Information** | | | | |
| --- | --- | --- | --- | --- |
| Model | Model Fitting Criteria | Likelihood Ratio Tests | | |
|  | -2 Log Likelihood | Chi-Square | df | Sig. |
| Intercept Only | 2638,500 |  |  |  |
| Final | 2605,289 | 33,211 | 16 | ,007 |

| **Pseudo R-Square** | |
| --- | --- |
| Cox and Snell | ,020 |
| Nagelkerke | ,021 |
| McFadden | ,006 |

| **Likelihood Ratio Tests** | | | | |
| --- | --- | --- | --- | --- |
| Effect | Model Fitting Criteria | Likelihood Ratio Tests | | |
|  | -2 Log Likelihood of Reduced Model | Chi-Square | df | Sig. |
| Intercept | 2605,289^a^ | ,000 | 0 | . |
| leeftijd | 2617,271 | 11,982 | 4 | ,017 |
| geslacht | 2608,212 | 2,924 | 4 | ,571 |
| cbs_opleidingsniveau | 2625,058 | 19,770 | 8 | ,011 |
| The chi-square statistic is the difference in -2 log-likelihoods between the final model and a reduced model. The reduced model is formed by omitting an effect from the final model. The null hypothesis is that all parameters of that effect are 0. | | | | |
| a. This reduced model is equivalent to the final model because omitting the effect does not increase the degrees of freedom. | | | | |

| **Parameter Estimates** | | | | | | | | | |
| --- | --- | --- | --- | --- | --- | --- | --- | --- | --- |
| 23. Als u zou mogen kiezen, waar zou u dan (als eerst) het geld aan besteden? Zet de volgende onderwerpen op volgorde van belangrijkheid. Nr. 1^a^ | | B | Std. Error | Wald | df | Sig. | Exp(B) | 95% Confidence Interval for Exp(B) | |
|  |  |  |  |  |  |  |  | Lower Bound | Upper Bound |
| Het verbeteren van de behandeling van darmkanker | Intercept | 1,372 | ,354 | 14,989 | 1 | ,000 |  |  |  |
|  | leeftijd | -,006 | ,007 | ,803 | 1 | ,370 | ,994 | ,981 | 1,007 |
|  | [geslacht=1] | ,252 | ,204 | 1,514 | 1 | ,219 | 1,286 | ,861 | 1,920 |
|  | [geslacht=2] | 0^b^ | . | . | 0 | . | . | . | . |
|  | [cbs_opleidingsniveau=1,00] | -,424 | ,269 | 2,490 | 1 | ,115 | ,654 | ,386 | 1,108 |
|  | [cbs_opleidingsniveau=2,00] | ,019 | ,264 | ,005 | 1 | ,944 | 1,019 | ,607 | 1,709 |
|  | [cbs_opleidingsniveau=3,00] | 0^b^ | . | . | 0 | . | . | . | . |
| Mensen aanbieden om zich preventief te laten onderzoeken op darmkanker (dit is het bevolkingsonderzoek darmkanker) | Intercept | ,795 | ,353 | 5,072 | 1 | ,024 |  |  |  |
|  | leeftijd | ,007 | ,007 | 1,240 | 1 | ,265 | 1,007 | ,994 | 1,020 |
|  | [geslacht=1] | ,219 | ,200 | 1,200 | 1 | ,273 | 1,244 | ,842 | 1,840 |
|  | [geslacht=2] | 0^b^ | . | . | 0 | . | . | . | . |
|  | [cbs_opleidingsniveau=1,00] | -,296 | ,265 | 1,249 | 1 | ,264 | ,744 | ,443 | 1,250 |
|  | [cbs_opleidingsniveau=2,00] | ,257 | ,261 | ,970 | 1 | ,325 | 1,293 | ,775 | 2,157 |
|  | [cbs_opleidingsniveau=3,00] | 0^b^ | . | . | 0 | . | . | . | . |
| Meer onderzoek doen naar de oorzaken van darmkanker | Intercept | ,586 | ,369 | 2,519 | 1 | ,113 |  |  |  |
|  | leeftijd | ,007 | ,007 | 1,002 | 1 | ,317 | 1,007 | ,993 | 1,021 |
|  | [geslacht=1] | ,264 | ,212 | 1,559 | 1 | ,212 | 1,303 | ,860 | 1,973 |
|  | [geslacht=2] | 0^b^ | . | . | 0 | . | . | . | . |
|  | [cbs_opleidingsniveau=1,00] | -,768 | ,280 | 7,515 | 1 | ,006 | ,464 | ,268 | ,803 |
|  | [cbs_opleidingsniveau=2,00] | ,087 | ,269 | ,103 | 1 | ,748 | 1,090 | ,643 | 1,848 |
|  | [cbs_opleidingsniveau=3,00] | 0^b^ | . | . | 0 | . | . | . | . |
| Het geven van voorlichting over de klachten en risicofactoren en over wat mensen zelf kunnen doen | Intercept | ,844 | ,361 | 5,455 | 1 | ,020 |  |  |  |
|  | leeftijd | ,008 | ,007 | 1,205 | 1 | ,272 | 1,008 | ,994 | 1,021 |
|  | [geslacht=1] | ,085 | ,207 | ,170 | 1 | ,680 | 1,089 | ,726 | 1,635 |
|  | [geslacht=2] | 0^b^ | . | . | 0 | . | . | . | . |
|  | [cbs_opleidingsniveau=1,00] | -,715 | ,272 | 6,909 | 1 | ,009 | ,489 | ,287 | ,834 |
|  | [cbs_opleidingsniveau=2,00] | -,096 | ,265 | ,130 | 1 | ,719 | ,909 | ,540 | 1,529 |
|  | [cbs_opleidingsniveau=3,00] | 0^b^ | . | . | 0 | . | . | . | . |
| a. The reference category is: Het verbeteren van testen/onderzoeken om vast te stellen of iemand darmkanker heeft. | | | | | | | | | |
| b. This parameter is set to zero because it is redundant. | | | | | | | | | |

NOMREG ANSWER.A019.01.01 (BASE=3 ORDER=ASCENDING) BY geslacht cbs_opleidingsniveau WITH leeftijd

/CRITERIA CIN(95) DELTA(0) MXITER(100) MXSTEP(5) CHKSEP(20) LCONVERGE(0) PCONVERGE(0.000001)

SINGULAR(0.00000001)

/MODEL

/STEPWISE=PIN(.05) POUT(0.1) MINEFFECT(0) RULE(SINGLE) ENTRYMETHOD(LR) REMOVALMETHOD(LR)

/INTERCEPT=INCLUDE

/PRINT=PARAMETER SUMMARY LRT CPS STEP MFI.

**Nominal Regression**

| **Notes** | | |
| --- | --- | --- |
| Output Created | | 22-JAN-2016 13:12:20 |
| Comments | |  |
| Input | Data | \\fs01-home.rivm.nl\home\doumal\Documents\RIVM_VUmc\Publieke opinie - deel A\Analyse vragenlijst 1 deel A\SPSS analyse publieke opinie vragenlijst 1\Resultaten publieke opinie vragenlijst 1_werkbestand 1.sav |
|  | Active Dataset | DataSet1 |
|  | Filter | <none> |
|  | Weight | <none> |
|  | Split File | <none> |
|  | N of Rows in Working Data File | 1679 |
| Missing Value Handling | Definition of Missing | User-defined missing values are treated as missing. |
|  | Cases Used | Statistics are based on all cases with valid data for all variables in the model. |
| Syntax | | NOMREG ANSWER.A019.01.01 (BASE=3 ORDER=ASCENDING) BY geslacht cbs_opleidingsniveau WITH leeftijd  /CRITERIA CIN(95) DELTA(0) MXITER(100) MXSTEP(5) CHKSEP(20) LCONVERGE(0) PCONVERGE(0.000001)  SINGULAR(0.00000001)  /MODEL  /STEPWISE=PIN(.05) POUT(0.1) MINEFFECT(0) RULE(SINGLE) ENTRYMETHOD(LR) REMOVALMETHOD(LR)  /INTERCEPT=INCLUDE  /PRINT=PARAMETER SUMMARY LRT CPS STEP MFI. |
| Resources | Processor Time | 00:00:00,09 |
|  | Elapsed Time | 00:00:00,13 |

| **Case Processing Summary** | | | |
| --- | --- | --- | --- |
|  | | N | Marginal Percentage |
| 23. Als u zou mogen kiezen, waar zou u dan (als eerst) het geld aan besteden? Zet de volgende onderwerpen op volgorde van belangrijkheid. Nr. 1 | Het verbeteren van de behandeling van darmkanker | 390 | 23,2% |
|  | Mensen aanbieden om zich preventief te laten onderzoeken op darmkanker (dit is het bevolkingsonderzoek darmkanker) | 480 | 28,6% |
|  | Meer onderzoek doen naar de oorzaken van darmkanker | 315 | 18,8% |
|  | Het geven van voorlichting over de klachten en risicofactoren en over wat mensen zelf kunnen doen | 358 | 21,3% |
|  | Het verbeteren van testen/onderzoeken om vast te stellen of iemand darmkanker heeft | 136 | 8,1% |
| geslacht | Man | 903 | 53,8% |
|  | Vrouw | 776 | 46,2% |
| cbs_opleidingsniveau | laag | 544 | 32,4% |
|  | mid | 681 | 40,6% |
|  | hoog | 454 | 27,0% |
| Valid | | 1679 | 100,0% |
| Missing | | 0 |  |
| Total | | 1679 |  |
| Subpopulation | | 344^a^ |  |
| a. The dependent variable has only one value observed in 71 (20,6%) subpopulations. | | | |

| **Model Fitting Information** | | | | |
| --- | --- | --- | --- | --- |
| Model | Model Fitting Criteria | Likelihood Ratio Tests | | |
|  | -2 Log Likelihood | Chi-Square | df | Sig. |
| Intercept Only | 2638,500 |  |  |  |
| Final | 2605,289 | 33,211 | 16 | ,007 |

| **Pseudo R-Square** | |
| --- | --- |
| Cox and Snell | ,020 |
| Nagelkerke | ,021 |
| McFadden | ,006 |

| **Likelihood Ratio Tests** | | | | |
| --- | --- | --- | --- | --- |
| Effect | Model Fitting Criteria | Likelihood Ratio Tests | | |
|  | -2 Log Likelihood of Reduced Model | Chi-Square | df | Sig. |
| Intercept | 2605,289^a^ | ,000 | 0 | . |
| leeftijd | 2617,271 | 11,982 | 4 | ,017 |
| geslacht | 2608,212 | 2,924 | 4 | ,571 |
| cbs_opleidingsniveau | 2625,058 | 19,770 | 8 | ,011 |
| The chi-square statistic is the difference in -2 log-likelihoods between the final model and a reduced model. The reduced model is formed by omitting an effect from the final model. The null hypothesis is that all parameters of that effect are 0. | | | | |
| a. This reduced model is equivalent to the final model because omitting the effect does not increase the degrees of freedom. | | | | |

| **Parameter Estimates** | | | | | | | | | |
| --- | --- | --- | --- | --- | --- | --- | --- | --- | --- |
| 23. Als u zou mogen kiezen, waar zou u dan (als eerst) het geld aan besteden? Zet de volgende onderwerpen op volgorde van belangrijkheid. Nr. 1^a^ | | B | Std. Error | Wald | df | Sig. | Exp(B) | 95% Confidence Interval for Exp(B) | |
|  |  |  |  |  |  |  |  | Lower Bound | Upper Bound |
| Het verbeteren van de behandeling van darmkanker | Intercept | ,785 | ,266 | 8,724 | 1 | ,003 |  |  |  |
|  | leeftijd | -,013 | ,005 | 6,578 | 1 | ,010 | ,987 | ,977 | ,997 |
|  | [geslacht=1] | -,013 | ,157 | ,007 | 1 | ,934 | ,987 | ,726 | 1,343 |
|  | [geslacht=2] | 0^b^ | . | . | 0 | . | . | . | . |
|  | [cbs_opleidingsniveau=1,00] | ,344 | ,209 | 2,712 | 1 | ,100 | 1,410 | ,937 | 2,124 |
|  | [cbs_opleidingsniveau=2,00] | -,068 | ,185 | ,135 | 1 | ,713 | ,934 | ,650 | 1,342 |
|  | [cbs_opleidingsniveau=3,00] | 0^b^ | . | . | 0 | . | . | . | . |
| Mensen aanbieden om zich preventief te laten onderzoeken op darmkanker (dit is het bevolkingsonderzoek darmkanker) | Intercept | ,208 | ,263 | ,628 | 1 | ,428 |  |  |  |
|  | leeftijd | ,000 | ,005 | ,006 | 1 | ,939 | 1,000 | ,991 | 1,010 |
|  | [geslacht=1] | -,046 | ,151 | ,093 | 1 | ,761 | ,955 | ,711 | 1,283 |
|  | [geslacht=2] | 0^b^ | . | . | 0 | . | . | . | . |
|  | [cbs_opleidingsniveau=1,00] | ,472 | ,203 | 5,442 | 1 | ,020 | 1,604 | 1,078 | 2,385 |
|  | [cbs_opleidingsniveau=2,00] | ,170 | ,180 | ,894 | 1 | ,344 | 1,186 | ,833 | 1,688 |
|  | [cbs_opleidingsniveau=3,00] | 0^b^ | . | . | 0 | . | . | . | . |
| Het geven van voorlichting over de klachten en risicofactoren en over wat mensen zelf kunnen doen | Intercept | ,258 | ,274 | ,883 | 1 | ,347 |  |  |  |
|  | leeftijd | ,001 | ,005 | ,011 | 1 | ,916 | 1,001 | ,990 | 1,011 |
|  | [geslacht=1] | -,179 | ,160 | 1,246 | 1 | ,264 | ,836 | ,611 | 1,145 |
|  | [geslacht=2] | 0^b^ | . | . | 0 | . | . | . | . |
|  | [cbs_opleidingsniveau=1,00] | ,053 | ,212 | ,062 | 1 | ,803 | 1,054 | ,696 | 1,598 |
|  | [cbs_opleidingsniveau=2,00] | -,182 | ,187 | ,952 | 1 | ,329 | ,833 | ,578 | 1,202 |
|  | [cbs_opleidingsniveau=3,00] | 0^b^ | . | . | 0 | . | . | . | . |
| Het verbeteren van testen/onderzoeken om vast te stellen of iemand darmkanker heeft | Intercept | -,586 | ,369 | 2,519 | 1 | ,113 |  |  |  |
|  | leeftijd | -,007 | ,007 | 1,002 | 1 | ,317 | ,993 | ,980 | 1,007 |
|  | [geslacht=1] | -,264 | ,212 | 1,559 | 1 | ,212 | ,768 | ,507 | 1,163 |
|  | [geslacht=2] | 0^b^ | . | . | 0 | . | . | . | . |
|  | [cbs_opleidingsniveau=1,00] | ,768 | ,280 | 7,515 | 1 | ,006 | 2,156 | 1,245 | 3,733 |
|  | [cbs_opleidingsniveau=2,00] | -,087 | ,269 | ,103 | 1 | ,748 | ,917 | ,541 | 1,554 |
|  | [cbs_opleidingsniveau=3,00] | 0^b^ | . | . | 0 | . | . | . | . |
| a. The reference category is: Meer onderzoek doen naar de oorzaken van darmkanker. | | | | | | | | | |
| b. This parameter is set to zero because it is redundant. | | | | | | | | | |

NOMREG ANSWER.A019.01.01 (BASE=4 ORDER=ASCENDING) BY geslacht cbs_opleidingsniveau WITH leeftijd

/CRITERIA CIN(95) DELTA(0) MXITER(100) MXSTEP(5) CHKSEP(20) LCONVERGE(0) PCONVERGE(0.000001)

SINGULAR(0.00000001)

/MODEL

/STEPWISE=PIN(.05) POUT(0.1) MINEFFECT(0) RULE(SINGLE) ENTRYMETHOD(LR) REMOVALMETHOD(LR)

/INTERCEPT=INCLUDE

/PRINT=PARAMETER SUMMARY LRT CPS STEP MFI.

**Nominal Regression**

| **Notes** | | |
| --- | --- | --- |
| Output Created | | 22-JAN-2016 13:15:10 |
| Comments | |  |
| Input | Data | \\fs01-home.rivm.nl\home\doumal\Documents\RIVM_VUmc\Publieke opinie - deel A\Analyse vragenlijst 1 deel A\SPSS analyse publieke opinie vragenlijst 1\Resultaten publieke opinie vragenlijst 1_werkbestand 1.sav |
|  | Active Dataset | DataSet1 |
|  | Filter | <none> |
|  | Weight | <none> |
|  | Split File | <none> |
|  | N of Rows in Working Data File | 1679 |
| Missing Value Handling | Definition of Missing | User-defined missing values are treated as missing. |
|  | Cases Used | Statistics are based on all cases with valid data for all variables in the model. |
| Syntax | | NOMREG ANSWER.A019.01.01 (BASE=4 ORDER=ASCENDING) BY geslacht cbs_opleidingsniveau WITH leeftijd  /CRITERIA CIN(95) DELTA(0) MXITER(100) MXSTEP(5) CHKSEP(20) LCONVERGE(0) PCONVERGE(0.000001)  SINGULAR(0.00000001)  /MODEL  /STEPWISE=PIN(.05) POUT(0.1) MINEFFECT(0) RULE(SINGLE) ENTRYMETHOD(LR) REMOVALMETHOD(LR)  /INTERCEPT=INCLUDE  /PRINT=PARAMETER SUMMARY LRT CPS STEP MFI. |
| Resources | Processor Time | 00:00:00,11 |
|  | Elapsed Time | 00:00:00,15 |

| **Case Processing Summary** | | | |
| --- | --- | --- | --- |
|  | | N | Marginal Percentage |
| 23. Als u zou mogen kiezen, waar zou u dan (als eerst) het geld aan besteden? Zet de volgende onderwerpen op volgorde van belangrijkheid. Nr. 1 | Het verbeteren van de behandeling van darmkanker | 390 | 23,2% |
|  | Mensen aanbieden om zich preventief te laten onderzoeken op darmkanker (dit is het bevolkingsonderzoek darmkanker) | 480 | 28,6% |
|  | Meer onderzoek doen naar de oorzaken van darmkanker | 315 | 18,8% |
|  | Het geven van voorlichting over de klachten en risicofactoren en over wat mensen zelf kunnen doen | 358 | 21,3% |
|  | Het verbeteren van testen/onderzoeken om vast te stellen of iemand darmkanker heeft | 136 | 8,1% |
| geslacht | Man | 903 | 53,8% |
|  | Vrouw | 776 | 46,2% |
| cbs_opleidingsniveau | laag | 544 | 32,4% |
|  | mid | 681 | 40,6% |
|  | hoog | 454 | 27,0% |
| Valid | | 1679 | 100,0% |
| Missing | | 0 |  |
| Total | | 1679 |  |
| Subpopulation | | 344^a^ |  |
| a. The dependent variable has only one value observed in 71 (20,6%) subpopulations. | | | |

| **Model Fitting Information** | | | | |
| --- | --- | --- | --- | --- |
| Model | Model Fitting Criteria | Likelihood Ratio Tests | | |
|  | -2 Log Likelihood | Chi-Square | df | Sig. |
| Intercept Only | 2638,500 |  |  |  |
| Final | 2605,289 | 33,211 | 16 | ,007 |

| **Pseudo R-Square** | |
| --- | --- |
| Cox and Snell | ,020 |
| Nagelkerke | ,021 |
| McFadden | ,006 |

| **Likelihood Ratio Tests** | | | | |
| --- | --- | --- | --- | --- |
| Effect | Model Fitting Criteria | Likelihood Ratio Tests | | |
|  | -2 Log Likelihood of Reduced Model | Chi-Square | df | Sig. |
| Intercept | 2605,289^a^ | ,000 | 0 | . |
| leeftijd | 2617,271 | 11,982 | 4 | ,017 |
| geslacht | 2608,212 | 2,924 | 4 | ,571 |
| cbs_opleidingsniveau | 2625,058 | 19,770 | 8 | ,011 |
| The chi-square statistic is the difference in -2 log-likelihoods between the final model and a reduced model. The reduced model is formed by omitting an effect from the final model. The null hypothesis is that all parameters of that effect are 0. | | | | |
| a. This reduced model is equivalent to the final model because omitting the effect does not increase the degrees of freedom. | | | | |

| **Parameter Estimates** | | | | | | | | | |
| --- | --- | --- | --- | --- | --- | --- | --- | --- | --- |
| 23. Als u zou mogen kiezen, waar zou u dan (als eerst) het geld aan besteden? Zet de volgende onderwerpen op volgorde van belangrijkheid. Nr. 1^a^ | | B | Std. Error | Wald | df | Sig. | Exp(B) | 95% Confidence Interval for Exp(B) | |
|  |  |  |  |  |  |  |  | Lower Bound | Upper Bound |
| Het verbeteren van de behandeling van darmkanker | Intercept | ,528 | ,255 | 4,271 | 1 | ,039 |  |  |  |
|  | leeftijd | -,014 | ,005 | 7,557 | 1 | ,006 | ,987 | ,977 | ,996 |
|  | [geslacht=1] | ,166 | ,151 | 1,206 | 1 | ,272 | 1,181 | ,878 | 1,588 |
|  | [geslacht=2] | 0^b^ | . | . | 0 | . | . | . | . |
|  | [cbs_opleidingsniveau=1,00] | ,291 | ,198 | 2,159 | 1 | ,142 | 1,338 | ,907 | 1,972 |
|  | [cbs_opleidingsniveau=2,00] | ,114 | ,180 | ,406 | 1 | ,524 | 1,121 | ,788 | 1,594 |
|  | [cbs_opleidingsniveau=3,00] | 0^b^ | . | . | 0 | . | . | . | . |
| Mensen aanbieden om zich preventief te laten onderzoeken op darmkanker (dit is het bevolkingsonderzoek darmkanker) | Intercept | -,049 | ,253 | ,038 | 1 | ,845 |  |  |  |
|  | leeftijd | ,000 | ,005 | ,001 | 1 | ,971 | 1,000 | ,991 | 1,009 |
|  | [geslacht=1] | ,133 | ,145 | ,847 | 1 | ,357 | 1,142 | ,860 | 1,517 |
|  | [geslacht=2] | 0^b^ | . | . | 0 | . | . | . | . |
|  | [cbs_opleidingsniveau=1,00] | ,419 | ,191 | 4,802 | 1 | ,028 | 1,521 | 1,045 | 2,214 |
|  | [cbs_opleidingsniveau=2,00] | ,353 | ,175 | 4,068 | 1 | ,044 | 1,423 | 1,010 | 2,005 |
|  | [cbs_opleidingsniveau=3,00] | 0^b^ | . | . | 0 | . | . | . | . |
| Meer onderzoek doen naar de oorzaken van darmkanker | Intercept | -,258 | ,274 | ,883 | 1 | ,347 |  |  |  |
|  | leeftijd | -,001 | ,005 | ,011 | 1 | ,916 | ,999 | ,989 | 1,010 |
|  | [geslacht=1] | ,179 | ,160 | 1,246 | 1 | ,264 | 1,196 | ,873 | 1,638 |
|  | [geslacht=2] | 0^b^ | . | . | 0 | . | . | . | . |
|  | [cbs_opleidingsniveau=1,00] | -,053 | ,212 | ,062 | 1 | ,803 | ,948 | ,626 | 1,438 |
|  | [cbs_opleidingsniveau=2,00] | ,182 | ,187 | ,952 | 1 | ,329 | 1,200 | ,832 | 1,731 |
|  | [cbs_opleidingsniveau=3,00] | 0^b^ | . | . | 0 | . | . | . | . |
| Het verbeteren van testen/onderzoeken om vast te stellen of iemand darmkanker heeft | Intercept | -,844 | ,361 | 5,455 | 1 | ,020 |  |  |  |
|  | leeftijd | -,008 | ,007 | 1,205 | 1 | ,272 | ,993 | ,979 | 1,006 |
|  | [geslacht=1] | -,085 | ,207 | ,170 | 1 | ,680 | ,918 | ,612 | 1,378 |
|  | [geslacht=2] | 0^b^ | . | . | 0 | . | . | . | . |
|  | [cbs_opleidingsniveau=1,00] | ,715 | ,272 | 6,909 | 1 | ,009 | 2,045 | 1,200 | 3,485 |
|  | [cbs_opleidingsniveau=2,00] | ,096 | ,265 | ,130 | 1 | ,719 | 1,100 | ,654 | 1,852 |
|  | [cbs_opleidingsniveau=3,00] | 0^b^ | . | . | 0 | . | . | . | . |
| a. The reference category is: Het geven van voorlichting over de klachten en risicofactoren en over wat mensen zelf kunnen doen. | | | | | | | | | |
| b. This parameter is set to zero because it is redundant. | | | | | | | | | |

NOMREG ANSWER.A019.01.02 (BASE=FIRST ORDER=ASCENDING) BY geslacht cbs_opleidingsniveau WITH leeftijd

/CRITERIA CIN(95) DELTA(0) MXITER(100) MXSTEP(5) CHKSEP(20) LCONVERGE(0) PCONVERGE(0.000001)

SINGULAR(0.00000001)

/MODEL

/STEPWISE=PIN(.05) POUT(0.1) MINEFFECT(0) RULE(SINGLE) ENTRYMETHOD(LR) REMOVALMETHOD(LR)

/INTERCEPT=INCLUDE

/PRINT=PARAMETER SUMMARY LRT CPS STEP MFI.

NOMREG ANSWER.A019.01.02 (BASE=LAST ORDER=ASCENDING) BY geslacht cbs_opleidingsniveau WITH leeftijd

/CRITERIA CIN(95) DELTA(0) MXITER(100) MXSTEP(5) CHKSEP(20) LCONVERGE(0) PCONVERGE(0.000001)

SINGULAR(0.00000001)

/MODEL

/STEPWISE=PIN(.05) POUT(0.1) MINEFFECT(0) RULE(SINGLE) ENTRYMETHOD(LR) REMOVALMETHOD(LR)

/INTERCEPT=INCLUDE

/PRINT=PARAMETER SUMMARY LRT CPS STEP MFI.

**Nominal Regression**

| **Notes** | | |
| --- | --- | --- |
| Output Created | | 22-JAN-2016 13:20:53 |
| Comments | |  |
| Input | Data | \\fs01-home.rivm.nl\home\doumal\Documents\RIVM_VUmc\Publieke opinie - deel A\Analyse vragenlijst 1 deel A\SPSS analyse publieke opinie vragenlijst 1\Resultaten publieke opinie vragenlijst 1_werkbestand 1.sav |
|  | Active Dataset | DataSet1 |
|  | Filter | <none> |
|  | Weight | <none> |
|  | Split File | <none> |
|  | N of Rows in Working Data File | 1679 |
| Missing Value Handling | Definition of Missing | User-defined missing values are treated as missing. |
|  | Cases Used | Statistics are based on all cases with valid data for all variables in the model. |
| Syntax | | NOMREG ANSWER.A019.01.02 (BASE=LAST ORDER=ASCENDING) BY geslacht cbs_opleidingsniveau WITH leeftijd  /CRITERIA CIN(95) DELTA(0) MXITER(100) MXSTEP(5) CHKSEP(20) LCONVERGE(0) PCONVERGE(0.000001)  SINGULAR(0.00000001)  /MODEL  /STEPWISE=PIN(.05) POUT(0.1) MINEFFECT(0) RULE(SINGLE) ENTRYMETHOD(LR) REMOVALMETHOD(LR)  /INTERCEPT=INCLUDE  /PRINT=PARAMETER SUMMARY LRT CPS STEP MFI. |
| Resources | Processor Time | 00:00:00,13 |
|  | Elapsed Time | 00:00:00,14 |

| **Case Processing Summary** | | | |
| --- | --- | --- | --- |
|  | | N | Marginal Percentage |
| 23. Als u zou mogen kiezen, waar zou u dan (als eerst) het geld aan besteden? Zet de volgende onderwerpen op volgorde van belangrijkheid. Nr. 2 | Het verbeteren van de behandeling van darmkanker | 349 | 20,8% |
|  | Mensen aanbieden om zich preventief te laten onderzoeken op darmkanker (dit is het bevolkingsonderzoek darmkanker) | 324 | 19,3% |
|  | Meer onderzoek doen naar de oorzaken van darmkanker | 364 | 21,7% |
|  | Het geven van voorlichting over de klachten en risicofactoren en over wat mensen zelf kunnen doen | 318 | 18,9% |
|  | Het verbeteren van testen/onderzoeken om vast te stellen of iemand darmkanker heeft | 324 | 19,3% |
| geslacht | Man | 903 | 53,8% |
|  | Vrouw | 776 | 46,2% |
| cbs_opleidingsniveau | laag | 544 | 32,4% |
|  | mid | 681 | 40,6% |
|  | hoog | 454 | 27,0% |
| Valid | | 1679 | 100,0% |
| Missing | | 0 |  |
| Total | | 1679 |  |
| Subpopulation | | 344^a^ |  |
| a. The dependent variable has only one value observed in 66 (19,2%) subpopulations. | | | |

| **Model Fitting Information** | | | | |
| --- | --- | --- | --- | --- |
| Model | Model Fitting Criteria | Likelihood Ratio Tests | | |
|  | -2 Log Likelihood | Chi-Square | df | Sig. |
| Intercept Only | 2771,756 |  |  |  |
| Final | 2755,567 | 16,189 | 16 | ,440 |

| **Pseudo R-Square** | |
| --- | --- |
| Cox and Snell | ,010 |
| Nagelkerke | ,010 |
| McFadden | ,003 |

| **Likelihood Ratio Tests** | | | | |
| --- | --- | --- | --- | --- |
| Effect | Model Fitting Criteria | Likelihood Ratio Tests | | |
|  | -2 Log Likelihood of Reduced Model | Chi-Square | df | Sig. |
| Intercept | 2755,567^a^ | ,000 | 0 | . |
| leeftijd | 2756,722 | 1,155 | 4 | ,885 |
| geslacht | 2763,790 | 8,222 | 4 | ,084 |
| cbs_opleidingsniveau | 2762,018 | 6,451 | 8 | ,597 |
| The chi-square statistic is the difference in -2 log-likelihoods between the final model and a reduced model. The reduced model is formed by omitting an effect from the final model. The null hypothesis is that all parameters of that effect are 0. | | | | |
| a. This reduced model is equivalent to the final model because omitting the effect does not increase the degrees of freedom. | | | | |

| **Parameter Estimates** | | | | | | | | | |
| --- | --- | --- | --- | --- | --- | --- | --- | --- | --- |
| 23. Als u zou mogen kiezen, waar zou u dan (als eerst) het geld aan besteden? Zet de volgende onderwerpen op volgorde van belangrijkheid. Nr. 2^a^ | | B | Std. Error | Wald | df | Sig. | Exp(B) | 95% Confidence Interval for Exp(B) | |
|  |  |  |  |  |  |  |  | Lower Bound | Upper Bound |
| Het verbeteren van de behandeling van darmkanker | Intercept | ,254 | ,275 | ,856 | 1 | ,355 |  |  |  |
|  | leeftijd | -,003 | ,005 | ,417 | 1 | ,519 | ,997 | ,987 | 1,007 |
|  | [geslacht=1] | ,031 | ,160 | ,038 | 1 | ,846 | 1,032 | ,754 | 1,411 |
|  | [geslacht=2] | 0^b^ | . | . | 0 | . | . | . | . |
|  | [cbs_opleidingsniveau=1,00] | -,224 | ,212 | 1,118 | 1 | ,290 | ,799 | ,528 | 1,211 |
|  | [cbs_opleidingsniveau=2,00] | ,096 | ,193 | ,246 | 1 | ,620 | 1,100 | ,754 | 1,607 |
|  | [cbs_opleidingsniveau=3,00] | 0^b^ | . | . | 0 | . | . | . | . |
| Mensen aanbieden om zich preventief te laten onderzoeken op darmkanker (dit is het bevolkingsonderzoek darmkanker) | Intercept | ,193 | ,280 | ,477 | 1 | ,490 |  |  |  |
|  | leeftijd | ,002 | ,005 | ,106 | 1 | ,745 | 1,002 | ,991 | 1,012 |
|  | [geslacht=1] | -,328 | ,162 | 4,083 | 1 | ,043 | ,720 | ,524 | ,990 |
|  | [geslacht=2] | 0^b^ | . | . | 0 | . | . | . | . |
|  | [cbs_opleidingsniveau=1,00] | -,250 | ,214 | 1,370 | 1 | ,242 | ,779 | ,512 | 1,184 |
|  | [cbs_opleidingsniveau=2,00] | -,067 | ,197 | ,116 | 1 | ,734 | ,935 | ,635 | 1,376 |
|  | [cbs_opleidingsniveau=3,00] | 0^b^ | . | . | 0 | . | . | . | . |
| Meer onderzoek doen naar de oorzaken van darmkanker | Intercept | ,055 | ,276 | ,039 | 1 | ,843 |  |  |  |
|  | leeftijd | ,000 | ,005 | ,001 | 1 | ,975 | 1,000 | ,990 | 1,010 |
|  | [geslacht=1] | ,055 | ,158 | ,119 | 1 | ,730 | 1,056 | ,775 | 1,440 |
|  | [geslacht=2] | 0^b^ | . | . | 0 | . | . | . | . |
|  | [cbs_opleidingsniveau=1,00] | ,052 | ,207 | ,063 | 1 | ,803 | 1,053 | ,702 | 1,580 |
|  | [cbs_opleidingsniveau=2,00] | ,052 | ,196 | ,071 | 1 | ,790 | 1,054 | ,718 | 1,546 |
|  | [cbs_opleidingsniveau=3,00] | 0^b^ | . | . | 0 | . | . | . | . |
| Het geven van voorlichting over de klachten en risicofactoren en over wat mensen zelf kunnen doen | Intercept | ,133 | ,281 | ,224 | 1 | ,636 |  |  |  |
|  | leeftijd | ,001 | ,005 | ,044 | 1 | ,834 | 1,001 | ,991 | 1,012 |
|  | [geslacht=1] | -,162 | ,163 | ,991 | 1 | ,319 | ,850 | ,617 | 1,170 |
|  | [geslacht=2] | 0^b^ | . | . | 0 | . | . | . | . |
|  | [cbs_opleidingsniveau=1,00] | -,263 | ,214 | 1,518 | 1 | ,218 | ,768 | ,505 | 1,168 |
|  | [cbs_opleidingsniveau=2,00] | -,083 | ,198 | ,176 | 1 | ,675 | ,920 | ,625 | 1,356 |
|  | [cbs_opleidingsniveau=3,00] | 0^b^ | . | . | 0 | . | . | . | . |
| a. The reference category is: Het verbeteren van testen/onderzoeken om vast te stellen of iemand darmkanker heeft. | | | | | | | | | |
| b. This parameter is set to zero because it is redundant. | | | | | | | | | |

NOMREG ANSWER.A019.01.02 (BASE=3 ORDER=ASCENDING) BY geslacht cbs_opleidingsniveau WITH leeftijd

/CRITERIA CIN(95) DELTA(0) MXITER(100) MXSTEP(5) CHKSEP(20) LCONVERGE(0) PCONVERGE(0.000001)

SINGULAR(0.00000001)

/MODEL

/STEPWISE=PIN(.05) POUT(0.1) MINEFFECT(0) RULE(SINGLE) ENTRYMETHOD(LR) REMOVALMETHOD(LR)

/INTERCEPT=INCLUDE

/PRINT=PARAMETER SUMMARY LRT CPS STEP MFI.

**Nominal Regression**

| **Notes** | | |
| --- | --- | --- |
| Output Created | | 22-JAN-2016 13:22:45 |
| Comments | |  |
| Input | Data | \\fs01-home.rivm.nl\home\doumal\Documents\RIVM_VUmc\Publieke opinie - deel A\Analyse vragenlijst 1 deel A\SPSS analyse publieke opinie vragenlijst 1\Resultaten publieke opinie vragenlijst 1_werkbestand 1.sav |
|  | Active Dataset | DataSet1 |
|  | Filter | <none> |
|  | Weight | <none> |
|  | Split File | <none> |
|  | N of Rows in Working Data File | 1679 |
| Missing Value Handling | Definition of Missing | User-defined missing values are treated as missing. |
|  | Cases Used | Statistics are based on all cases with valid data for all variables in the model. |
| Syntax | | NOMREG ANSWER.A019.01.02 (BASE=3 ORDER=ASCENDING) BY geslacht cbs_opleidingsniveau WITH leeftijd  /CRITERIA CIN(95) DELTA(0) MXITER(100) MXSTEP(5) CHKSEP(20) LCONVERGE(0) PCONVERGE(0.000001)  SINGULAR(0.00000001)  /MODEL  /STEPWISE=PIN(.05) POUT(0.1) MINEFFECT(0) RULE(SINGLE) ENTRYMETHOD(LR) REMOVALMETHOD(LR)  /INTERCEPT=INCLUDE  /PRINT=PARAMETER SUMMARY LRT CPS STEP MFI. |
| Resources | Processor Time | 00:00:00,11 |
|  | Elapsed Time | 00:00:00,12 |

| **Case Processing Summary** | | | |
| --- | --- | --- | --- |
|  | | N | Marginal Percentage |
| 23. Als u zou mogen kiezen, waar zou u dan (als eerst) het geld aan besteden? Zet de volgende onderwerpen op volgorde van belangrijkheid. Nr. 2 | Het verbeteren van de behandeling van darmkanker | 349 | 20,8% |
|  | Mensen aanbieden om zich preventief te laten onderzoeken op darmkanker (dit is het bevolkingsonderzoek darmkanker) | 324 | 19,3% |
|  | Meer onderzoek doen naar de oorzaken van darmkanker | 364 | 21,7% |
|  | Het geven van voorlichting over de klachten en risicofactoren en over wat mensen zelf kunnen doen | 318 | 18,9% |
|  | Het verbeteren van testen/onderzoeken om vast te stellen of iemand darmkanker heeft | 324 | 19,3% |
| geslacht | Man | 903 | 53,8% |
|  | Vrouw | 776 | 46,2% |
| cbs_opleidingsniveau | laag | 544 | 32,4% |
|  | mid | 681 | 40,6% |
|  | hoog | 454 | 27,0% |
| Valid | | 1679 | 100,0% |
| Missing | | 0 |  |
| Total | | 1679 |  |
| Subpopulation | | 344^a^ |  |
| a. The dependent variable has only one value observed in 66 (19,2%) subpopulations. | | | |

| **Model Fitting Information** | | | | |
| --- | --- | --- | --- | --- |
| Model | Model Fitting Criteria | Likelihood Ratio Tests | | |
|  | -2 Log Likelihood | Chi-Square | df | Sig. |
| Intercept Only | 2771,756 |  |  |  |
| Final | 2755,567 | 16,189 | 16 | ,440 |

| **Pseudo R-Square** | |
| --- | --- |
| Cox and Snell | ,010 |
| Nagelkerke | ,010 |
| McFadden | ,003 |

| **Likelihood Ratio Tests** | | | | |
| --- | --- | --- | --- | --- |
| Effect | Model Fitting Criteria | Likelihood Ratio Tests | | |
|  | -2 Log Likelihood of Reduced Model | Chi-Square | df | Sig. |
| Intercept | 2755,567^a^ | ,000 | 0 | . |
| leeftijd | 2756,722 | 1,155 | 4 | ,885 |
| geslacht | 2763,790 | 8,222 | 4 | ,084 |
| cbs_opleidingsniveau | 2762,018 | 6,451 | 8 | ,597 |
| The chi-square statistic is the difference in -2 log-likelihoods between the final model and a reduced model. The reduced model is formed by omitting an effect from the final model. The null hypothesis is that all parameters of that effect are 0. | | | | |
| a. This reduced model is equivalent to the final model because omitting the effect does not increase the degrees of freedom. | | | | |

| **Parameter Estimates** | | | | | | | | | |
| --- | --- | --- | --- | --- | --- | --- | --- | --- | --- |
| 23. Als u zou mogen kiezen, waar zou u dan (als eerst) het geld aan besteden? Zet de volgende onderwerpen op volgorde van belangrijkheid. Nr. 2^a^ | | B | Std. Error | Wald | df | Sig. | Exp(B) | 95% Confidence Interval for Exp(B) | |
|  |  |  |  |  |  |  |  | Lower Bound | Upper Bound |
| Het verbeteren van de behandeling van darmkanker | Intercept | ,199 | ,268 | ,555 | 1 | ,456 |  |  |  |
|  | leeftijd | -,003 | ,005 | ,401 | 1 | ,526 | ,997 | ,987 | 1,007 |
|  | [geslacht=1] | -,024 | ,155 | ,023 | 1 | ,879 | ,977 | ,720 | 1,324 |
|  | [geslacht=2] | 0^b^ | . | . | 0 | . | . | . | . |
|  | [cbs_opleidingsniveau=1,00] | -,276 | ,206 | 1,783 | 1 | ,182 | ,759 | ,507 | 1,138 |
|  | [cbs_opleidingsniveau=2,00] | ,044 | ,188 | ,054 | 1 | ,817 | 1,045 | ,722 | 1,510 |
|  | [cbs_opleidingsniveau=3,00] | 0^b^ | . | . | 0 | . | . | . | . |
| Mensen aanbieden om zich preventief te laten onderzoeken op darmkanker (dit is het bevolkingsonderzoek darmkanker) | Intercept | ,139 | ,273 | ,257 | 1 | ,612 |  |  |  |
|  | leeftijd | ,002 | ,005 | ,133 | 1 | ,715 | 1,002 | ,992 | 1,012 |
|  | [geslacht=1] | -,383 | ,158 | 5,862 | 1 | ,015 | ,682 | ,500 | ,930 |
|  | [geslacht=2] | 0^b^ | . | . | 0 | . | . | . | . |
|  | [cbs_opleidingsniveau=1,00] | -,302 | ,209 | 2,096 | 1 | ,148 | ,739 | ,491 | 1,113 |
|  | [cbs_opleidingsniveau=2,00] | -,119 | ,192 | ,384 | 1 | ,536 | ,888 | ,609 | 1,294 |
|  | [cbs_opleidingsniveau=3,00] | 0^b^ | . | . | 0 | . | . | . | . |
| Het geven van voorlichting over de klachten en risicofactoren en over wat mensen zelf kunnen doen | Intercept | ,078 | ,274 | ,081 | 1 | ,776 |  |  |  |
|  | leeftijd | ,001 | ,005 | ,061 | 1 | ,805 | 1,001 | ,991 | 1,011 |
|  | [geslacht=1] | -,217 | ,159 | 1,868 | 1 | ,172 | ,805 | ,590 | 1,099 |
|  | [geslacht=2] | 0^b^ | . | . | 0 | . | . | . | . |
|  | [cbs_opleidingsniveau=1,00] | -,315 | ,209 | 2,284 | 1 | ,131 | ,730 | ,485 | 1,098 |
|  | [cbs_opleidingsniveau=2,00] | -,135 | ,193 | ,491 | 1 | ,484 | ,874 | ,599 | 1,275 |
|  | [cbs_opleidingsniveau=3,00] | 0^b^ | . | . | 0 | . | . | . | . |
| Het verbeteren van testen/onderzoeken om vast te stellen of iemand darmkanker heeft | Intercept | -,055 | ,276 | ,039 | 1 | ,843 |  |  |  |
|  | leeftijd | ,000 | ,005 | ,001 | 1 | ,975 | 1,000 | ,990 | 1,010 |
|  | [geslacht=1] | -,055 | ,158 | ,119 | 1 | ,730 | ,947 | ,694 | 1,291 |
|  | [geslacht=2] | 0^b^ | . | . | 0 | . | . | . | . |
|  | [cbs_opleidingsniveau=1,00] | -,052 | ,207 | ,063 | 1 | ,803 | ,950 | ,633 | 1,424 |
|  | [cbs_opleidingsniveau=2,00] | -,052 | ,196 | ,071 | 1 | ,790 | ,949 | ,647 | 1,393 |
|  | [cbs_opleidingsniveau=3,00] | 0^b^ | . | . | 0 | . | . | . | . |
| a. The reference category is: Meer onderzoek doen naar de oorzaken van darmkanker. | | | | | | | | | |
| b. This parameter is set to zero because it is redundant. | | | | | | | | | |

NOMREG ANSWER.A019.01.02 (BASE=4 ORDER=ASCENDING) BY geslacht cbs_opleidingsniveau WITH leeftijd

/CRITERIA CIN(95) DELTA(0) MXITER(100) MXSTEP(5) CHKSEP(20) LCONVERGE(0) PCONVERGE(0.000001)

SINGULAR(0.00000001)

/MODEL

/STEPWISE=PIN(.05) POUT(0.1) MINEFFECT(0) RULE(SINGLE) ENTRYMETHOD(LR) REMOVALMETHOD(LR)

/INTERCEPT=INCLUDE

/PRINT=PARAMETER SUMMARY LRT CPS STEP MFI.

**Nominal Regression**

| **Notes** | | |
| --- | --- | --- |
| Output Created | | 22-JAN-2016 13:24:53 |
| Comments | |  |
| Input | Data | \\fs01-home.rivm.nl\home\doumal\Documents\RIVM_VUmc\Publieke opinie - deel A\Analyse vragenlijst 1 deel A\SPSS analyse publieke opinie vragenlijst 1\Resultaten publieke opinie vragenlijst 1_werkbestand 1.sav |
|  | Active Dataset | DataSet1 |
|  | Filter | <none> |
|  | Weight | <none> |
|  | Split File | <none> |
|  | N of Rows in Working Data File | 1679 |
| Missing Value Handling | Definition of Missing | User-defined missing values are treated as missing. |
|  | Cases Used | Statistics are based on all cases with valid data for all variables in the model. |
| Syntax | | NOMREG ANSWER.A019.01.02 (BASE=4 ORDER=ASCENDING) BY geslacht cbs_opleidingsniveau WITH leeftijd  /CRITERIA CIN(95) DELTA(0) MXITER(100) MXSTEP(5) CHKSEP(20) LCONVERGE(0) PCONVERGE(0.000001)  SINGULAR(0.00000001)  /MODEL  /STEPWISE=PIN(.05) POUT(0.1) MINEFFECT(0) RULE(SINGLE) ENTRYMETHOD(LR) REMOVALMETHOD(LR)  /INTERCEPT=INCLUDE  /PRINT=PARAMETER SUMMARY LRT CPS STEP MFI. |
| Resources | Processor Time | 00:00:00,11 |
|  | Elapsed Time | 00:00:00,13 |

| **Case Processing Summary** | | | |
| --- | --- | --- | --- |
|  | | N | Marginal Percentage |
| 23. Als u zou mogen kiezen, waar zou u dan (als eerst) het geld aan besteden? Zet de volgende onderwerpen op volgorde van belangrijkheid. Nr. 2 | Het verbeteren van de behandeling van darmkanker | 349 | 20,8% |
|  | Mensen aanbieden om zich preventief te laten onderzoeken op darmkanker (dit is het bevolkingsonderzoek darmkanker) | 324 | 19,3% |
|  | Meer onderzoek doen naar de oorzaken van darmkanker | 364 | 21,7% |
|  | Het geven van voorlichting over de klachten en risicofactoren en over wat mensen zelf kunnen doen | 318 | 18,9% |
|  | Het verbeteren van testen/onderzoeken om vast te stellen of iemand darmkanker heeft | 324 | 19,3% |
| geslacht | Man | 903 | 53,8% |
|  | Vrouw | 776 | 46,2% |
| cbs_opleidingsniveau | laag | 544 | 32,4% |
|  | mid | 681 | 40,6% |
|  | hoog | 454 | 27,0% |
| Valid | | 1679 | 100,0% |
| Missing | | 0 |  |
| Total | | 1679 |  |
| Subpopulation | | 344^a^ |  |
| a. The dependent variable has only one value observed in 66 (19,2%) subpopulations. | | | |

| **Model Fitting Information** | | | | |
| --- | --- | --- | --- | --- |
| Model | Model Fitting Criteria | Likelihood Ratio Tests | | |
|  | -2 Log Likelihood | Chi-Square | df | Sig. |
| Intercept Only | 2771,756 |  |  |  |
| Final | 2755,567 | 16,189 | 16 | ,440 |

| **Pseudo R-Square** | |
| --- | --- |
| Cox and Snell | ,010 |
| Nagelkerke | ,010 |
| McFadden | ,003 |

| **Likelihood Ratio Tests** | | | | |
| --- | --- | --- | --- | --- |
| Effect | Model Fitting Criteria | Likelihood Ratio Tests | | |
|  | -2 Log Likelihood of Reduced Model | Chi-Square | df | Sig. |
| Intercept | 2755,567^a^ | ,000 | 0 | . |
| leeftijd | 2756,722 | 1,155 | 4 | ,885 |
| geslacht | 2763,790 | 8,222 | 4 | ,084 |
| cbs_opleidingsniveau | 2762,018 | 6,451 | 8 | ,597 |
| The chi-square statistic is the difference in -2 log-likelihoods between the final model and a reduced model. The reduced model is formed by omitting an effect from the final model. The null hypothesis is that all parameters of that effect are 0. | | | | |
| a. This reduced model is equivalent to the final model because omitting the effect does not increase the degrees of freedom. | | | | |

| **Parameter Estimates** | | | | | | | | | |
| --- | --- | --- | --- | --- | --- | --- | --- | --- | --- |
| 23. Als u zou mogen kiezen, waar zou u dan (als eerst) het geld aan besteden? Zet de volgende onderwerpen op volgorde van belangrijkheid. Nr. 2^a^ | | B | Std. Error | Wald | df | Sig. | Exp(B) | 95% Confidence Interval for Exp(B) | |
|  |  |  |  |  |  |  |  | Lower Bound | Upper Bound |
| Het verbeteren van de behandeling van darmkanker | Intercept | ,121 | ,273 | ,198 | 1 | ,657 |  |  |  |
|  | leeftijd | -,004 | ,005 | ,732 | 1 | ,392 | ,996 | ,986 | 1,006 |
|  | [geslacht=1] | ,194 | ,160 | 1,458 | 1 | ,227 | 1,214 | ,886 | 1,661 |
|  | [geslacht=2] | 0^b^ | . | . | 0 | . | . | . | . |
|  | [cbs_opleidingsniveau=1,00] | ,040 | ,213 | ,035 | 1 | ,853 | 1,040 | ,685 | 1,580 |
|  | [cbs_opleidingsniveau=2,00] | ,179 | ,190 | ,883 | 1 | ,347 | 1,196 | ,824 | 1,735 |
|  | [cbs_opleidingsniveau=3,00] | 0^b^ | . | . | 0 | . | . | . | . |
| Mensen aanbieden om zich preventief te laten onderzoeken op darmkanker (dit is het bevolkingsonderzoek darmkanker) | Intercept | ,060 | ,278 | ,047 | 1 | ,828 |  |  |  |
|  | leeftijd | ,001 | ,005 | ,013 | 1 | ,909 | 1,001 | ,990 | 1,011 |
|  | [geslacht=1] | -,166 | ,163 | 1,035 | 1 | ,309 | ,847 | ,616 | 1,166 |
|  | [geslacht=2] | 0^b^ | . | . | 0 | . | . | . | . |
|  | [cbs_opleidingsniveau=1,00] | ,013 | ,215 | ,004 | 1 | ,951 | 1,013 | ,665 | 1,545 |
|  | [cbs_opleidingsniveau=2,00] | ,016 | ,194 | ,007 | 1 | ,935 | 1,016 | ,695 | 1,486 |
|  | [cbs_opleidingsniveau=3,00] | 0^b^ | . | . | 0 | . | . | . | . |
| Meer onderzoek doen naar de oorzaken van darmkanker | Intercept | -,078 | ,274 | ,081 | 1 | ,776 |  |  |  |
|  | leeftijd | -,001 | ,005 | ,061 | 1 | ,805 | ,999 | ,989 | 1,009 |
|  | [geslacht=1] | ,217 | ,159 | 1,868 | 1 | ,172 | 1,242 | ,910 | 1,696 |
|  | [geslacht=2] | 0^b^ | . | . | 0 | . | . | . | . |
|  | [cbs_opleidingsniveau=1,00] | ,315 | ,209 | 2,284 | 1 | ,131 | 1,370 | ,911 | 2,062 |
|  | [cbs_opleidingsniveau=2,00] | ,135 | ,193 | ,491 | 1 | ,484 | 1,145 | ,784 | 1,670 |
|  | [cbs_opleidingsniveau=3,00] | 0^b^ | . | . | 0 | . | . | . | . |
| Het verbeteren van testen/onderzoeken om vast te stellen of iemand darmkanker heeft | Intercept | -,133 | ,281 | ,224 | 1 | ,636 |  |  |  |
|  | leeftijd | -,001 | ,005 | ,044 | 1 | ,834 | ,999 | ,989 | 1,009 |
|  | [geslacht=1] | ,162 | ,163 | ,991 | 1 | ,319 | 1,176 | ,854 | 1,620 |
|  | [geslacht=2] | 0^b^ | . | . | 0 | . | . | . | . |
|  | [cbs_opleidingsniveau=1,00] | ,263 | ,214 | 1,518 | 1 | ,218 | 1,301 | ,856 | 1,979 |
|  | [cbs_opleidingsniveau=2,00] | ,083 | ,198 | ,176 | 1 | ,675 | 1,086 | ,738 | 1,600 |
|  | [cbs_opleidingsniveau=3,00] | 0^b^ | . | . | 0 | . | . | . | . |
| a. The reference category is: Het geven van voorlichting over de klachten en risicofactoren en over wat mensen zelf kunnen doen. | | | | | | | | | |
| b. This parameter is set to zero because it is redundant. | | | | | | | | | |

NOMREG ANSWER.A019.01.03 (BASE=LAST ORDER=ASCENDING) BY geslacht cbs_opleidingsniveau WITH leeftijd

/CRITERIA CIN(95) DELTA(0) MXITER(100) MXSTEP(5) CHKSEP(20) LCONVERGE(0) PCONVERGE(0.000001)

SINGULAR(0.00000001)

/MODEL

/STEPWISE=PIN(.05) POUT(0.1) MINEFFECT(0) RULE(SINGLE) ENTRYMETHOD(LR) REMOVALMETHOD(LR)

/INTERCEPT=INCLUDE

/PRINT=PARAMETER SUMMARY LRT CPS STEP MFI.

**Nominal Regression**

| **Notes** | | |
| --- | --- | --- |
| Output Created | | 22-JAN-2016 13:26:25 |
| Comments | |  |
| Input | Data | \\fs01-home.rivm.nl\home\doumal\Documents\RIVM_VUmc\Publieke opinie - deel A\Analyse vragenlijst 1 deel A\SPSS analyse publieke opinie vragenlijst 1\Resultaten publieke opinie vragenlijst 1_werkbestand 1.sav |
|  | Active Dataset | DataSet1 |
|  | Filter | <none> |
|  | Weight | <none> |
|  | Split File | <none> |
|  | N of Rows in Working Data File | 1679 |
| Missing Value Handling | Definition of Missing | User-defined missing values are treated as missing. |
|  | Cases Used | Statistics are based on all cases with valid data for all variables in the model. |
| Syntax | | NOMREG ANSWER.A019.01.03 (BASE=LAST ORDER=ASCENDING) BY geslacht cbs_opleidingsniveau WITH leeftijd  /CRITERIA CIN(95) DELTA(0) MXITER(100) MXSTEP(5) CHKSEP(20) LCONVERGE(0) PCONVERGE(0.000001)  SINGULAR(0.00000001)  /MODEL  /STEPWISE=PIN(.05) POUT(0.1) MINEFFECT(0) RULE(SINGLE) ENTRYMETHOD(LR) REMOVALMETHOD(LR)  /INTERCEPT=INCLUDE  /PRINT=PARAMETER SUMMARY LRT CPS STEP MFI. |
| Resources | Processor Time | 00:00:00,09 |
|  | Elapsed Time | 00:00:00,12 |

| **Case Processing Summary** | | | |
| --- | --- | --- | --- |
|  | | N | Marginal Percentage |
| 23. Als u zou mogen kiezen, waar zou u dan (als eerst) het geld aan besteden? Zet de volgende onderwerpen op volgorde van belangrijkheid. Nr. 3 | Het verbeteren van de behandeling van darmkanker | 354 | 21,1% |
|  | Mensen aanbieden om zich preventief te laten onderzoeken op darmkanker (dit is het bevolkingsonderzoek darmkanker) | 283 | 16,9% |
|  | Meer onderzoek doen naar de oorzaken van darmkanker | 367 | 21,9% |
|  | Het geven van voorlichting over de klachten en risicofactoren en over wat mensen zelf kunnen doen | 255 | 15,2% |
|  | Het verbeteren van testen/onderzoeken om vast te stellen of iemand darmkanker heeft | 420 | 25,0% |
| geslacht | Man | 903 | 53,8% |
|  | Vrouw | 776 | 46,2% |
| cbs_opleidingsniveau | laag | 544 | 32,4% |
|  | mid | 681 | 40,6% |
|  | hoog | 454 | 27,0% |
| Valid | | 1679 | 100,0% |
| Missing | | 0 |  |
| Total | | 1679 |  |
| Subpopulation | | 344^a^ |  |
| a. The dependent variable has only one value observed in 73 (21,2%) subpopulations. | | | |

| **Model Fitting Information** | | | | |
| --- | --- | --- | --- | --- |
| Model | Model Fitting Criteria | Likelihood Ratio Tests | | |
|  | -2 Log Likelihood | Chi-Square | df | Sig. |
| Intercept Only | 2754,662 |  |  |  |
| Final | 2745,101 | 9,561 | 16 | ,889 |

| **Pseudo R-Square** | |
| --- | --- |
| Cox and Snell | ,006 |
| Nagelkerke | ,006 |
| McFadden | ,002 |

| **Likelihood Ratio Tests** | | | | |
| --- | --- | --- | --- | --- |
| Effect | Model Fitting Criteria | Likelihood Ratio Tests | | |
|  | -2 Log Likelihood of Reduced Model | Chi-Square | df | Sig. |
| Intercept | 2745,101^a^ | ,000 | 0 | . |
| leeftijd | 2746,223 | 1,122 | 4 | ,891 |
| geslacht | 2749,301 | 4,200 | 4 | ,380 |
| cbs_opleidingsniveau | 2748,839 | 3,738 | 8 | ,880 |
| The chi-square statistic is the difference in -2 log-likelihoods between the final model and a reduced model. The reduced model is formed by omitting an effect from the final model. The null hypothesis is that all parameters of that effect are 0. | | | | |
| a. This reduced model is equivalent to the final model because omitting the effect does not increase the degrees of freedom. | | | | |

| **Parameter Estimates** | | | | | | | | | |
| --- | --- | --- | --- | --- | --- | --- | --- | --- | --- |
| 23. Als u zou mogen kiezen, waar zou u dan (als eerst) het geld aan besteden? Zet de volgende onderwerpen op volgorde van belangrijkheid. Nr. 3^a^ | | B | Std. Error | Wald | df | Sig. | Exp(B) | 95% Confidence Interval for Exp(B) | |
|  |  |  |  |  |  |  |  | Lower Bound | Upper Bound |
| Het verbeteren van de behandeling van darmkanker | Intercept | -,140 | ,258 | ,295 | 1 | ,587 |  |  |  |
|  | leeftijd | ,002 | ,005 | ,211 | 1 | ,646 | 1,002 | ,993 | 1,012 |
|  | [geslacht=1] | -,291 | ,149 | 3,790 | 1 | ,052 | ,748 | ,558 | 1,002 |
|  | [geslacht=2] | 0^b^ | . | . | 0 | . | . | . | . |
|  | [cbs_opleidingsniveau=1,00] | ,125 | ,198 | ,401 | 1 | ,526 | 1,134 | ,769 | 1,671 |
|  | [cbs_opleidingsniveau=2,00] | -,061 | ,181 | ,114 | 1 | ,735 | ,941 | ,660 | 1,341 |
|  | [cbs_opleidingsniveau=3,00] | 0^b^ | . | . | 0 | . | . | . | . |
| Mensen aanbieden om zich preventief te laten onderzoeken op darmkanker (dit is het bevolkingsonderzoek darmkanker) | Intercept | -,103 | ,270 | ,144 | 1 | ,704 |  |  |  |
|  | leeftijd | -,003 | ,005 | ,333 | 1 | ,564 | ,997 | ,987 | 1,007 |
|  | [geslacht=1] | -,147 | ,159 | ,859 | 1 | ,354 | ,863 | ,632 | 1,179 |
|  | [geslacht=2] | 0^b^ | . | . | 0 | . | . | . | . |
|  | [cbs_opleidingsniveau=1,00] | -,063 | ,211 | ,090 | 1 | ,764 | ,939 | ,621 | 1,419 |
|  | [cbs_opleidingsniveau=2,00] | -,109 | ,189 | ,333 | 1 | ,564 | ,897 | ,620 | 1,298 |
|  | [cbs_opleidingsniveau=3,00] | 0^b^ | . | . | 0 | . | . | . | . |
| Meer onderzoek doen naar de oorzaken van darmkanker | Intercept | -,145 | ,256 | ,318 | 1 | ,573 |  |  |  |
|  | leeftijd | ,001 | ,005 | ,047 | 1 | ,829 | 1,001 | ,992 | 1,010 |
|  | [geslacht=1] | -,166 | ,148 | 1,257 | 1 | ,262 | ,847 | ,634 | 1,132 |
|  | [geslacht=2] | 0^b^ | . | . | 0 | . | . | . | . |
|  | [cbs_opleidingsniveau=1,00] | ,161 | ,196 | ,668 | 1 | ,414 | 1,174 | ,799 | 1,726 |
|  | [cbs_opleidingsniveau=2,00] | -,004 | ,180 | ,000 | 1 | ,982 | ,996 | ,701 | 1,416 |
|  | [cbs_opleidingsniveau=3,00] | 0^b^ | . | . | 0 | . | . | . | . |
| Het geven van voorlichting over de klachten en risicofactoren en over wat mensen zelf kunnen doen | Intercept | -,372 | ,282 | 1,742 | 1 | ,187 |  |  |  |
|  | leeftijd | ,002 | ,005 | ,109 | 1 | ,741 | 1,002 | ,991 | 1,012 |
|  | [geslacht=1] | -,227 | ,164 | 1,909 | 1 | ,167 | ,797 | ,577 | 1,100 |
|  | [geslacht=2] | 0^b^ | . | . | 0 | . | . | . | . |
|  | [cbs_opleidingsniveau=1,00] | ,007 | ,214 | ,001 | 1 | ,974 | 1,007 | ,662 | 1,532 |
|  | [cbs_opleidingsniveau=2,00] | -,232 | ,197 | 1,384 | 1 | ,239 | ,793 | ,539 | 1,167 |
|  | [cbs_opleidingsniveau=3,00] | 0^b^ | . | . | 0 | . | . | . | . |
| a. The reference category is: Het verbeteren van testen/onderzoeken om vast te stellen of iemand darmkanker heeft. | | | | | | | | | |
| b. This parameter is set to zero because it is redundant. | | | | | | | | | |

NOMREG ANSWER.A019.01.03 (BASE=3 ORDER=ASCENDING) BY geslacht cbs_opleidingsniveau WITH leeftijd

/CRITERIA CIN(95) DELTA(0) MXITER(100) MXSTEP(5) CHKSEP(20) LCONVERGE(0) PCONVERGE(0.000001)

SINGULAR(0.00000001)

/MODEL

/STEPWISE=PIN(.05) POUT(0.1) MINEFFECT(0) RULE(SINGLE) ENTRYMETHOD(LR) REMOVALMETHOD(LR)

/INTERCEPT=INCLUDE

/PRINT=PARAMETER SUMMARY LRT CPS STEP MFI.

**Nominal Regression**

| **Notes** | | |
| --- | --- | --- |
| Output Created | | 22-JAN-2016 13:27:04 |
| Comments | |  |
| Input | Data | \\fs01-home.rivm.nl\home\doumal\Documents\RIVM_VUmc\Publieke opinie - deel A\Analyse vragenlijst 1 deel A\SPSS analyse publieke opinie vragenlijst 1\Resultaten publieke opinie vragenlijst 1_werkbestand 1.sav |
|  | Active Dataset | DataSet1 |
|  | Filter | <none> |
|  | Weight | <none> |
|  | Split File | <none> |
|  | N of Rows in Working Data File | 1679 |
| Missing Value Handling | Definition of Missing | User-defined missing values are treated as missing. |
|  | Cases Used | Statistics are based on all cases with valid data for all variables in the model. |
| Syntax | | NOMREG ANSWER.A019.01.03 (BASE=3 ORDER=ASCENDING) BY geslacht cbs_opleidingsniveau WITH leeftijd  /CRITERIA CIN(95) DELTA(0) MXITER(100) MXSTEP(5) CHKSEP(20) LCONVERGE(0) PCONVERGE(0.000001)  SINGULAR(0.00000001)  /MODEL  /STEPWISE=PIN(.05) POUT(0.1) MINEFFECT(0) RULE(SINGLE) ENTRYMETHOD(LR) REMOVALMETHOD(LR)  /INTERCEPT=INCLUDE  /PRINT=PARAMETER SUMMARY LRT CPS STEP MFI. |
| Resources | Processor Time | 00:00:00,12 |
|  | Elapsed Time | 00:00:00,13 |

| **Case Processing Summary** | | | |
| --- | --- | --- | --- |
|  | | N | Marginal Percentage |
| 23. Als u zou mogen kiezen, waar zou u dan (als eerst) het geld aan besteden? Zet de volgende onderwerpen op volgorde van belangrijkheid. Nr. 3 | Het verbeteren van de behandeling van darmkanker | 354 | 21,1% |
|  | Mensen aanbieden om zich preventief te laten onderzoeken op darmkanker (dit is het bevolkingsonderzoek darmkanker) | 283 | 16,9% |
|  | Meer onderzoek doen naar de oorzaken van darmkanker | 367 | 21,9% |
|  | Het geven van voorlichting over de klachten en risicofactoren en over wat mensen zelf kunnen doen | 255 | 15,2% |
|  | Het verbeteren van testen/onderzoeken om vast te stellen of iemand darmkanker heeft | 420 | 25,0% |
| geslacht | Man | 903 | 53,8% |
|  | Vrouw | 776 | 46,2% |
| cbs_opleidingsniveau | laag | 544 | 32,4% |
|  | mid | 681 | 40,6% |
|  | hoog | 454 | 27,0% |
| Valid | | 1679 | 100,0% |
| Missing | | 0 |  |
| Total | | 1679 |  |
| Subpopulation | | 344^a^ |  |
| a. The dependent variable has only one value observed in 73 (21,2%) subpopulations. | | | |

| **Model Fitting Information** | | | | |
| --- | --- | --- | --- | --- |
| Model | Model Fitting Criteria | Likelihood Ratio Tests | | |
|  | -2 Log Likelihood | Chi-Square | df | Sig. |
| Intercept Only | 2754,662 |  |  |  |
| Final | 2745,101 | 9,561 | 16 | ,889 |

| **Pseudo R-Square** | |
| --- | --- |
| Cox and Snell | ,006 |
| Nagelkerke | ,006 |
| McFadden | ,002 |

| **Likelihood Ratio Tests** | | | | |
| --- | --- | --- | --- | --- |
| Effect | Model Fitting Criteria | Likelihood Ratio Tests | | |
|  | -2 Log Likelihood of Reduced Model | Chi-Square | df | Sig. |
| Intercept | 2745,101^a^ | ,000 | 0 | . |
| leeftijd | 2746,223 | 1,122 | 4 | ,891 |
| geslacht | 2749,301 | 4,200 | 4 | ,380 |
| cbs_opleidingsniveau | 2748,839 | 3,738 | 8 | ,880 |
| The chi-square statistic is the difference in -2 log-likelihoods between the final model and a reduced model. The reduced model is formed by omitting an effect from the final model. The null hypothesis is that all parameters of that effect are 0. | | | | |
| a. This reduced model is equivalent to the final model because omitting the effect does not increase the degrees of freedom. | | | | |

| **Parameter Estimates** | | | | | | | | | |
| --- | --- | --- | --- | --- | --- | --- | --- | --- | --- |
| 23. Als u zou mogen kiezen, waar zou u dan (als eerst) het geld aan besteden? Zet de volgende onderwerpen op volgorde van belangrijkheid. Nr. 3^a^ | | B | Std. Error | Wald | df | Sig. | Exp(B) | 95% Confidence Interval for Exp(B) | |
|  |  |  |  |  |  |  |  | Lower Bound | Upper Bound |
| Het verbeteren van de behandeling van darmkanker | Intercept | ,004 | ,268 | ,000 | 1 | ,988 |  |  |  |
|  | leeftijd | ,001 | ,005 | ,057 | 1 | ,812 | 1,001 | ,991 | 1,011 |
|  | [geslacht=1] | -,125 | ,153 | ,661 | 1 | ,416 | ,883 | ,653 | 1,192 |
|  | [geslacht=2] | 0^b^ | . | . | 0 | . | . | . | . |
|  | [cbs_opleidingsniveau=1,00] | -,035 | ,204 | ,030 | 1 | ,863 | ,965 | ,647 | 1,440 |
|  | [cbs_opleidingsniveau=2,00] | -,057 | ,189 | ,092 | 1 | ,762 | ,944 | ,652 | 1,368 |
|  | [cbs_opleidingsniveau=3,00] | 0^b^ | . | . | 0 | . | . | . | . |
| Mensen aanbieden om zich preventief te laten onderzoeken op darmkanker (dit is het bevolkingsonderzoek darmkanker) | Intercept | ,042 | ,279 | ,023 | 1 | ,880 |  |  |  |
|  | leeftijd | -,004 | ,005 | ,567 | 1 | ,451 | ,996 | ,986 | 1,006 |
|  | [geslacht=1] | ,018 | ,163 | ,013 | 1 | ,910 | 1,019 | ,740 | 1,403 |
|  | [geslacht=2] | 0^b^ | . | . | 0 | . | . | . | . |
|  | [cbs_opleidingsniveau=1,00] | -,224 | ,216 | 1,070 | 1 | ,301 | ,799 | ,523 | 1,222 |
|  | [cbs_opleidingsniveau=2,00] | -,105 | ,196 | ,285 | 1 | ,593 | ,900 | ,613 | 1,323 |
|  | [cbs_opleidingsniveau=3,00] | 0^b^ | . | . | 0 | . | . | . | . |
| Het geven van voorlichting over de klachten en risicofactoren en over wat mensen zelf kunnen doen | Intercept | -,227 | ,290 | ,613 | 1 | ,434 |  |  |  |
|  | leeftijd | ,001 | ,005 | ,018 | 1 | ,894 | 1,001 | ,990 | 1,012 |
|  | [geslacht=1] | -,061 | ,168 | ,132 | 1 | ,716 | ,941 | ,677 | 1,308 |
|  | [geslacht=2] | 0^b^ | . | . | 0 | . | . | . | . |
|  | [cbs_opleidingsniveau=1,00] | -,154 | ,220 | ,490 | 1 | ,484 | ,858 | ,558 | 1,319 |
|  | [cbs_opleidingsniveau=2,00] | -,228 | ,205 | 1,241 | 1 | ,265 | ,796 | ,533 | 1,189 |
|  | [cbs_opleidingsniveau=3,00] | 0^b^ | . | . | 0 | . | . | . | . |
| Het verbeteren van testen/onderzoeken om vast te stellen of iemand darmkanker heeft | Intercept | ,145 | ,256 | ,318 | 1 | ,573 |  |  |  |
|  | leeftijd | -,001 | ,005 | ,047 | 1 | ,829 | ,999 | ,990 | 1,008 |
|  | [geslacht=1] | ,166 | ,148 | 1,257 | 1 | ,262 | 1,180 | ,883 | 1,578 |
|  | [geslacht=2] | 0^b^ | . | . | 0 | . | . | . | . |
|  | [cbs_opleidingsniveau=1,00] | -,161 | ,196 | ,668 | 1 | ,414 | ,852 | ,579 | 1,252 |
|  | [cbs_opleidingsniveau=2,00] | ,004 | ,180 | ,000 | 1 | ,982 | 1,004 | ,706 | 1,427 |
|  | [cbs_opleidingsniveau=3,00] | 0^b^ | . | . | 0 | . | . | . | . |
| a. The reference category is: Meer onderzoek doen naar de oorzaken van darmkanker. | | | | | | | | | |
| b. This parameter is set to zero because it is redundant. | | | | | | | | | |

NOMREG ANSWER.A019.01.03 (BASE=4 ORDER=ASCENDING) BY geslacht cbs_opleidingsniveau WITH leeftijd

/CRITERIA CIN(95) DELTA(0) MXITER(100) MXSTEP(5) CHKSEP(20) LCONVERGE(0) PCONVERGE(0.000001)

SINGULAR(0.00000001)

/MODEL

/STEPWISE=PIN(.05) POUT(0.1) MINEFFECT(0) RULE(SINGLE) ENTRYMETHOD(LR) REMOVALMETHOD(LR)

/INTERCEPT=INCLUDE

/PRINT=PARAMETER SUMMARY LRT CPS STEP MFI.

**Nominal Regression**

| **Notes** | | |
| --- | --- | --- |
| Output Created | | 22-JAN-2016 13:27:26 |
| Comments | |  |
| Input | Data | \\fs01-home.rivm.nl\home\doumal\Documents\RIVM_VUmc\Publieke opinie - deel A\Analyse vragenlijst 1 deel A\SPSS analyse publieke opinie vragenlijst 1\Resultaten publieke opinie vragenlijst 1_werkbestand 1.sav |
|  | Active Dataset | DataSet1 |
|  | Filter | <none> |
|  | Weight | <none> |
|  | Split File | <none> |
|  | N of Rows in Working Data File | 1679 |
| Missing Value Handling | Definition of Missing | User-defined missing values are treated as missing. |
|  | Cases Used | Statistics are based on all cases with valid data for all variables in the model. |
| Syntax | | NOMREG ANSWER.A019.01.03 (BASE=4 ORDER=ASCENDING) BY geslacht cbs_opleidingsniveau WITH leeftijd  /CRITERIA CIN(95) DELTA(0) MXITER(100) MXSTEP(5) CHKSEP(20) LCONVERGE(0) PCONVERGE(0.000001)  SINGULAR(0.00000001)  /MODEL  /STEPWISE=PIN(.05) POUT(0.1) MINEFFECT(0) RULE(SINGLE) ENTRYMETHOD(LR) REMOVALMETHOD(LR)  /INTERCEPT=INCLUDE  /PRINT=PARAMETER SUMMARY LRT CPS STEP MFI. |
| Resources | Processor Time | 00:00:00,11 |
|  | Elapsed Time | 00:00:00,12 |

| **Case Processing Summary** | | | |
| --- | --- | --- | --- |
|  | | N | Marginal Percentage |
| 23. Als u zou mogen kiezen, waar zou u dan (als eerst) het geld aan besteden? Zet de volgende onderwerpen op volgorde van belangrijkheid. Nr. 3 | Het verbeteren van de behandeling van darmkanker | 354 | 21,1% |
|  | Mensen aanbieden om zich preventief te laten onderzoeken op darmkanker (dit is het bevolkingsonderzoek darmkanker) | 283 | 16,9% |
|  | Meer onderzoek doen naar de oorzaken van darmkanker | 367 | 21,9% |
|  | Het geven van voorlichting over de klachten en risicofactoren en over wat mensen zelf kunnen doen | 255 | 15,2% |
|  | Het verbeteren van testen/onderzoeken om vast te stellen of iemand darmkanker heeft | 420 | 25,0% |
| geslacht | Man | 903 | 53,8% |
|  | Vrouw | 776 | 46,2% |
| cbs_opleidingsniveau | laag | 544 | 32,4% |
|  | mid | 681 | 40,6% |
|  | hoog | 454 | 27,0% |
| Valid | | 1679 | 100,0% |
| Missing | | 0 |  |
| Total | | 1679 |  |
| Subpopulation | | 344^a^ |  |
| a. The dependent variable has only one value observed in 73 (21,2%) subpopulations. | | | |

| **Model Fitting Information** | | | | |
| --- | --- | --- | --- | --- |
| Model | Model Fitting Criteria | Likelihood Ratio Tests | | |
|  | -2 Log Likelihood | Chi-Square | df | Sig. |
| Intercept Only | 2754,662 |  |  |  |
| Final | 2745,101 | 9,561 | 16 | ,889 |

| **Pseudo R-Square** | |
| --- | --- |
| Cox and Snell | ,006 |
| Nagelkerke | ,006 |
| McFadden | ,002 |

| **Likelihood Ratio Tests** | | | | |
| --- | --- | --- | --- | --- |
| Effect | Model Fitting Criteria | Likelihood Ratio Tests | | |
|  | -2 Log Likelihood of Reduced Model | Chi-Square | df | Sig. |
| Intercept | 2745,101^a^ | ,000 | 0 | . |
| leeftijd | 2746,223 | 1,122 | 4 | ,891 |
| geslacht | 2749,301 | 4,200 | 4 | ,380 |
| cbs_opleidingsniveau | 2748,839 | 3,738 | 8 | ,880 |
| The chi-square statistic is the difference in -2 log-likelihoods between the final model and a reduced model. The reduced model is formed by omitting an effect from the final model. The null hypothesis is that all parameters of that effect are 0. | | | | |
| a. This reduced model is equivalent to the final model because omitting the effect does not increase the degrees of freedom. | | | | |

| **Parameter Estimates** | | | | | | | | | |
| --- | --- | --- | --- | --- | --- | --- | --- | --- | --- |
| 23. Als u zou mogen kiezen, waar zou u dan (als eerst) het geld aan besteden? Zet de volgende onderwerpen op volgorde van belangrijkheid. Nr. 3^a^ | | B | Std. Error | Wald | df | Sig. | Exp(B) | 95% Confidence Interval for Exp(B) | |
|  |  |  |  |  |  |  |  | Lower Bound | Upper Bound |
| Het verbeteren van de behandeling van darmkanker | Intercept | ,231 | ,292 | ,627 | 1 | ,428 |  |  |  |
|  | leeftijd | ,000 | ,006 | ,007 | 1 | ,933 | 1,000 | ,990 | 1,011 |
|  | [geslacht=1] | -,064 | ,169 | ,141 | 1 | ,707 | ,938 | ,673 | 1,308 |
|  | [geslacht=2] | 0^b^ | . | . | 0 | . | . | . | . |
|  | [cbs_opleidingsniveau=1,00] | ,118 | ,221 | ,288 | 1 | ,592 | 1,126 | ,730 | 1,736 |
|  | [cbs_opleidingsniveau=2,00] | ,171 | ,206 | ,688 | 1 | ,407 | 1,186 | ,792 | 1,777 |
|  | [cbs_opleidingsniveau=3,00] | 0^b^ | . | . | 0 | . | . | . | . |
| Mensen aanbieden om zich preventief te laten onderzoeken op darmkanker (dit is het bevolkingsonderzoek darmkanker) | Intercept | ,269 | ,302 | ,793 | 1 | ,373 |  |  |  |
|  | leeftijd | -,005 | ,006 | ,661 | 1 | ,416 | ,995 | ,984 | 1,007 |
|  | [geslacht=1] | ,080 | ,178 | ,200 | 1 | ,655 | 1,083 | ,764 | 1,535 |
|  | [geslacht=2] | 0^b^ | . | . | 0 | . | . | . | . |
|  | [cbs_opleidingsniveau=1,00] | -,070 | ,233 | ,091 | 1 | ,763 | ,932 | ,591 | 1,471 |
|  | [cbs_opleidingsniveau=2,00] | ,123 | ,213 | ,336 | 1 | ,562 | 1,131 | ,745 | 1,717 |
|  | [cbs_opleidingsniveau=3,00] | 0^b^ | . | . | 0 | . | . | . | . |
| Meer onderzoek doen naar de oorzaken van darmkanker | Intercept | ,227 | ,290 | ,613 | 1 | ,434 |  |  |  |
|  | leeftijd | -,001 | ,005 | ,018 | 1 | ,894 | ,999 | ,989 | 1,010 |
|  | [geslacht=1] | ,061 | ,168 | ,132 | 1 | ,716 | 1,063 | ,765 | 1,478 |
|  | [geslacht=2] | 0^b^ | . | . | 0 | . | . | . | . |
|  | [cbs_opleidingsniveau=1,00] | ,154 | ,220 | ,490 | 1 | ,484 | 1,166 | ,758 | 1,793 |
|  | [cbs_opleidingsniveau=2,00] | ,228 | ,205 | 1,241 | 1 | ,265 | 1,256 | ,841 | 1,877 |
|  | [cbs_opleidingsniveau=3,00] | 0^b^ | . | . | 0 | . | . | . | . |
| Het verbeteren van testen/onderzoeken om vast te stellen of iemand darmkanker heeft | Intercept | ,372 | ,282 | 1,742 | 1 | ,187 |  |  |  |
|  | leeftijd | -,002 | ,005 | ,109 | 1 | ,741 | ,998 | ,988 | 1,009 |
|  | [geslacht=1] | ,227 | ,164 | 1,909 | 1 | ,167 | 1,255 | ,909 | 1,732 |
|  | [geslacht=2] | 0^b^ | . | . | 0 | . | . | . | . |
|  | [cbs_opleidingsniveau=1,00] | -,007 | ,214 | ,001 | 1 | ,974 | ,993 | ,653 | 1,511 |
|  | [cbs_opleidingsniveau=2,00] | ,232 | ,197 | 1,384 | 1 | ,239 | 1,261 | ,857 | 1,857 |
|  | [cbs_opleidingsniveau=3,00] | 0^b^ | . | . | 0 | . | . | . | . |
| a. The reference category is: Het geven van voorlichting over de klachten en risicofactoren en over wat mensen zelf kunnen doen. | | | | | | | | | |
| b. This parameter is set to zero because it is redundant. | | | | | | | | | |

NOMREG ANSWER.A019.01.04 (BASE=LAST ORDER=ASCENDING) BY geslacht cbs_opleidingsniveau WITH leeftijd

/CRITERIA CIN(95) DELTA(0) MXITER(100) MXSTEP(5) CHKSEP(20) LCONVERGE(0) PCONVERGE(0.000001)

SINGULAR(0.00000001)

/MODEL

/STEPWISE=PIN(.05) POUT(0.1) MINEFFECT(0) RULE(SINGLE) ENTRYMETHOD(LR) REMOVALMETHOD(LR)

/INTERCEPT=INCLUDE

/PRINT=PARAMETER SUMMARY LRT CPS STEP MFI.

**Nominal Regression**

| **Notes** | | |
| --- | --- | --- |
| Output Created | | 22-JAN-2016 13:30:37 |
| Comments | |  |
| Input | Data | \\fs01-home.rivm.nl\home\doumal\Documents\RIVM_VUmc\Publieke opinie - deel A\Analyse vragenlijst 1 deel A\SPSS analyse publieke opinie vragenlijst 1\Resultaten publieke opinie vragenlijst 1_werkbestand 1.sav |
|  | Active Dataset | DataSet1 |
|  | Filter | <none> |
|  | Weight | <none> |
|  | Split File | <none> |
|  | N of Rows in Working Data File | 1679 |
| Missing Value Handling | Definition of Missing | User-defined missing values are treated as missing. |
|  | Cases Used | Statistics are based on all cases with valid data for all variables in the model. |
| Syntax | | NOMREG ANSWER.A019.01.04 (BASE=LAST ORDER=ASCENDING) BY geslacht cbs_opleidingsniveau WITH leeftijd  /CRITERIA CIN(95) DELTA(0) MXITER(100) MXSTEP(5) CHKSEP(20) LCONVERGE(0) PCONVERGE(0.000001)  SINGULAR(0.00000001)  /MODEL  /STEPWISE=PIN(.05) POUT(0.1) MINEFFECT(0) RULE(SINGLE) ENTRYMETHOD(LR) REMOVALMETHOD(LR)  /INTERCEPT=INCLUDE  /PRINT=PARAMETER SUMMARY LRT CPS STEP MFI. |
| Resources | Processor Time | 00:00:00,14 |
|  | Elapsed Time | 00:00:00,14 |

| **Case Processing Summary** | | | |
| --- | --- | --- | --- |
|  | | N | Marginal Percentage |
| 23. Als u zou mogen kiezen, waar zou u dan (als eerst) het geld aan besteden? Zet de volgende onderwerpen op volgorde van belangrijkheid. Nr. 4 | Het verbeteren van de behandeling van darmkanker | 313 | 18,6% |
|  | Mensen aanbieden om zich preventief te laten onderzoeken op darmkanker (dit is het bevolkingsonderzoek darmkanker) | 277 | 16,5% |
|  | Meer onderzoek doen naar de oorzaken van darmkanker | 374 | 22,3% |
|  | Het geven van voorlichting over de klachten en risicofactoren en over wat mensen zelf kunnen doen | 309 | 18,4% |
|  | Het verbeteren van testen/onderzoeken om vast te stellen of iemand darmkanker heeft | 406 | 24,2% |
| geslacht | Man | 903 | 53,8% |
|  | Vrouw | 776 | 46,2% |
| cbs_opleidingsniveau | laag | 544 | 32,4% |
|  | mid | 681 | 40,6% |
|  | hoog | 454 | 27,0% |
| Valid | | 1679 | 100,0% |
| Missing | | 0 |  |
| Total | | 1679 |  |
| Subpopulation | | 344^a^ |  |
| a. The dependent variable has only one value observed in 69 (20,1%) subpopulations. | | | |

| **Model Fitting Information** | | | | |
| --- | --- | --- | --- | --- |
| Model | Model Fitting Criteria | Likelihood Ratio Tests | | |
|  | -2 Log Likelihood | Chi-Square | df | Sig. |
| Intercept Only | 2760,801 |  |  |  |
| Final | 2734,282 | 26,520 | 16 | ,047 |

| **Pseudo R-Square** | |
| --- | --- |
| Cox and Snell | ,016 |
| Nagelkerke | ,016 |
| McFadden | ,005 |

| **Likelihood Ratio Tests** | | | | |
| --- | --- | --- | --- | --- |
| Effect | Model Fitting Criteria | Likelihood Ratio Tests | | |
|  | -2 Log Likelihood of Reduced Model | Chi-Square | df | Sig. |
| Intercept | 2734,282^a^ | ,000 | 0 | . |
| leeftijd | 2737,022 | 2,741 | 4 | ,602 |
| geslacht | 2744,588 | 10,306 | 4 | ,036 |
| cbs_opleidingsniveau | 2745,193 | 10,912 | 8 | ,207 |
| The chi-square statistic is the difference in -2 log-likelihoods between the final model and a reduced model. The reduced model is formed by omitting an effect from the final model. The null hypothesis is that all parameters of that effect are 0. | | | | |
| a. This reduced model is equivalent to the final model because omitting the effect does not increase the degrees of freedom. | | | | |

| **Parameter Estimates** | | | | | | | | | |
| --- | --- | --- | --- | --- | --- | --- | --- | --- | --- |
| 23. Als u zou mogen kiezen, waar zou u dan (als eerst) het geld aan besteden? Zet de volgende onderwerpen op volgorde van belangrijkheid. Nr. 4^a^ | | B | Std. Error | Wald | df | Sig. | Exp(B) | 95% Confidence Interval for Exp(B) | |
|  |  |  |  |  |  |  |  | Lower Bound | Upper Bound |
| Het verbeteren van de behandeling van darmkanker | Intercept | -,803 | ,272 | 8,722 | 1 | ,003 |  |  |  |
|  | leeftijd | ,007 | ,005 | 1,823 | 1 | ,177 | 1,007 | ,997 | 1,017 |
|  | [geslacht=1] | -,090 | ,155 | ,333 | 1 | ,564 | ,914 | ,674 | 1,239 |
|  | [geslacht=2] | 0^b^ | . | . | 0 | . | . | . | . |
|  | [cbs_opleidingsniveau=1,00] | ,252 | ,211 | 1,431 | 1 | ,232 | 1,286 | ,851 | 1,944 |
|  | [cbs_opleidingsniveau=2,00] | ,396 | ,191 | 4,294 | 1 | ,038 | 1,486 | 1,022 | 2,161 |
|  | [cbs_opleidingsniveau=3,00] | 0^b^ | . | . | 0 | . | . | . | . |
| Mensen aanbieden om zich preventief te laten onderzoeken op darmkanker (dit is het bevolkingsonderzoek darmkanker) | Intercept | -,650 | ,276 | 5,570 | 1 | ,018 |  |  |  |
|  | leeftijd | ,002 | ,005 | ,086 | 1 | ,770 | 1,002 | ,991 | 1,012 |
|  | [geslacht=1] | ,291 | ,162 | 3,208 | 1 | ,073 | 1,338 | ,973 | 1,839 |
|  | [geslacht=2] | 0^b^ | . | . | 0 | . | . | . | . |
|  | [cbs_opleidingsniveau=1,00] | ,158 | ,207 | ,581 | 1 | ,446 | 1,171 | ,781 | 1,756 |
|  | [cbs_opleidingsniveau=2,00] | -,068 | ,195 | ,123 | 1 | ,726 | ,934 | ,638 | 1,368 |
|  | [cbs_opleidingsniveau=3,00] | 0^b^ | . | . | 0 | . | . | . | . |
| Meer onderzoek doen naar de oorzaken van darmkanker | Intercept | -,024 | ,248 | ,009 | 1 | ,923 |  |  |  |
|  | leeftijd | ,002 | ,005 | ,107 | 1 | ,743 | 1,002 | ,992 | 1,011 |
|  | [geslacht=1] | -,183 | ,148 | 1,530 | 1 | ,216 | ,833 | ,624 | 1,113 |
|  | [geslacht=2] | 0^b^ | . | . | 0 | . | . | . | . |
|  | [cbs_opleidingsniveau=1,00] | -,083 | ,194 | ,185 | 1 | ,667 | ,920 | ,629 | 1,345 |
|  | [cbs_opleidingsniveau=2,00] | -,043 | ,175 | ,062 | 1 | ,804 | ,958 | ,680 | 1,349 |
|  | [cbs_opleidingsniveau=3,00] | 0^b^ | . | . | 0 | . | . | . | . |
| Het geven van voorlichting over de klachten en risicofactoren en over wat mensen zelf kunnen doen | Intercept | -,830 | ,272 | 9,299 | 1 | ,002 |  |  |  |
|  | leeftijd | ,006 | ,005 | 1,403 | 1 | ,236 | 1,006 | ,996 | 1,016 |
|  | [geslacht=1] | ,144 | ,156 | ,845 | 1 | ,358 | 1,155 | ,850 | 1,569 |
|  | [geslacht=2] | 0^b^ | . | . | 0 | . | . | . | . |
|  | [cbs_opleidingsniveau=1,00] | ,212 | ,207 | 1,043 | 1 | ,307 | 1,236 | ,823 | 1,856 |
|  | [cbs_opleidingsniveau=2,00] | ,266 | ,190 | 1,962 | 1 | ,161 | 1,305 | ,899 | 1,895 |
|  | [cbs_opleidingsniveau=3,00] | 0^b^ | . | . | 0 | . | . | . | . |
| a. The reference category is: Het verbeteren van testen/onderzoeken om vast te stellen of iemand darmkanker heeft. | | | | | | | | | |
| b. This parameter is set to zero because it is redundant. | | | | | | | | | |

NOMREG ANSWER.A019.01.04 (BASE=3 ORDER=ASCENDING) BY geslacht cbs_opleidingsniveau WITH leeftijd

/CRITERIA CIN(95) DELTA(0) MXITER(100) MXSTEP(5) CHKSEP(20) LCONVERGE(0) PCONVERGE(0.000001)

SINGULAR(0.00000001)

/MODEL

/STEPWISE=PIN(.05) POUT(0.1) MINEFFECT(0) RULE(SINGLE) ENTRYMETHOD(LR) REMOVALMETHOD(LR)

/INTERCEPT=INCLUDE

/PRINT=PARAMETER SUMMARY LRT CPS STEP MFI.

**Nominal Regression**

| **Notes** | | |
| --- | --- | --- |
| Output Created | | 22-JAN-2016 13:31:24 |
| Comments | |  |
| Input | Data | \\fs01-home.rivm.nl\home\doumal\Documents\RIVM_VUmc\Publieke opinie - deel A\Analyse vragenlijst 1 deel A\SPSS analyse publieke opinie vragenlijst 1\Resultaten publieke opinie vragenlijst 1_werkbestand 1.sav |
|  | Active Dataset | DataSet1 |
|  | Filter | <none> |
|  | Weight | <none> |
|  | Split File | <none> |
|  | N of Rows in Working Data File | 1679 |
| Missing Value Handling | Definition of Missing | User-defined missing values are treated as missing. |
|  | Cases Used | Statistics are based on all cases with valid data for all variables in the model. |
| Syntax | | NOMREG ANSWER.A019.01.04 (BASE=3 ORDER=ASCENDING) BY geslacht cbs_opleidingsniveau WITH leeftijd  /CRITERIA CIN(95) DELTA(0) MXITER(100) MXSTEP(5) CHKSEP(20) LCONVERGE(0) PCONVERGE(0.000001)  SINGULAR(0.00000001)  /MODEL  /STEPWISE=PIN(.05) POUT(0.1) MINEFFECT(0) RULE(SINGLE) ENTRYMETHOD(LR) REMOVALMETHOD(LR)  /INTERCEPT=INCLUDE  /PRINT=PARAMETER SUMMARY LRT CPS STEP MFI. |
| Resources | Processor Time | 00:00:00,13 |
|  | Elapsed Time | 00:00:00,13 |

| **Case Processing Summary** | | | |
| --- | --- | --- | --- |
|  | | N | Marginal Percentage |
| 23. Als u zou mogen kiezen, waar zou u dan (als eerst) het geld aan besteden? Zet de volgende onderwerpen op volgorde van belangrijkheid. Nr. 4 | Het verbeteren van de behandeling van darmkanker | 313 | 18,6% |
|  | Mensen aanbieden om zich preventief te laten onderzoeken op darmkanker (dit is het bevolkingsonderzoek darmkanker) | 277 | 16,5% |
|  | Meer onderzoek doen naar de oorzaken van darmkanker | 374 | 22,3% |
|  | Het geven van voorlichting over de klachten en risicofactoren en over wat mensen zelf kunnen doen | 309 | 18,4% |
|  | Het verbeteren van testen/onderzoeken om vast te stellen of iemand darmkanker heeft | 406 | 24,2% |
| geslacht | Man | 903 | 53,8% |
|  | Vrouw | 776 | 46,2% |
| cbs_opleidingsniveau | laag | 544 | 32,4% |
|  | mid | 681 | 40,6% |
|  | hoog | 454 | 27,0% |
| Valid | | 1679 | 100,0% |
| Missing | | 0 |  |
| Total | | 1679 |  |
| Subpopulation | | 344^a^ |  |
| a. The dependent variable has only one value observed in 69 (20,1%) subpopulations. | | | |

| **Model Fitting Information** | | | | |
| --- | --- | --- | --- | --- |
| Model | Model Fitting Criteria | Likelihood Ratio Tests | | |
|  | -2 Log Likelihood | Chi-Square | df | Sig. |
| Intercept Only | 2760,801 |  |  |  |
| Final | 2734,282 | 26,520 | 16 | ,047 |

| **Pseudo R-Square** | |
| --- | --- |
| Cox and Snell | ,016 |
| Nagelkerke | ,016 |
| McFadden | ,005 |

| **Likelihood Ratio Tests** | | | | |
| --- | --- | --- | --- | --- |
| Effect | Model Fitting Criteria | Likelihood Ratio Tests | | |
|  | -2 Log Likelihood of Reduced Model | Chi-Square | df | Sig. |
| Intercept | 2734,282^a^ | ,000 | 0 | . |
| leeftijd | 2737,022 | 2,741 | 4 | ,602 |
| geslacht | 2744,588 | 10,306 | 4 | ,036 |
| cbs_opleidingsniveau | 2745,193 | 10,912 | 8 | ,207 |
| The chi-square statistic is the difference in -2 log-likelihoods between the final model and a reduced model. The reduced model is formed by omitting an effect from the final model. The null hypothesis is that all parameters of that effect are 0. | | | | |
| a. This reduced model is equivalent to the final model because omitting the effect does not increase the degrees of freedom. | | | | |

| **Parameter Estimates** | | | | | | | | | |
| --- | --- | --- | --- | --- | --- | --- | --- | --- | --- |
| 23. Als u zou mogen kiezen, waar zou u dan (als eerst) het geld aan besteden? Zet de volgende onderwerpen op volgorde van belangrijkheid. Nr. 4^a^ | | B | Std. Error | Wald | df | Sig. | Exp(B) | 95% Confidence Interval for Exp(B) | |
|  |  |  |  |  |  |  |  | Lower Bound | Upper Bound |
| Het verbeteren van de behandeling van darmkanker | Intercept | -,780 | ,276 | 7,970 | 1 | ,005 |  |  |  |
|  | leeftijd | ,005 | ,005 | 1,033 | 1 | ,310 | 1,005 | ,995 | 1,015 |
|  | [geslacht=1] | ,093 | ,158 | ,346 | 1 | ,556 | 1,097 | ,805 | 1,496 |
|  | [geslacht=2] | 0^b^ | . | . | 0 | . | . | . | . |
|  | [cbs_opleidingsniveau=1,00] | ,335 | ,215 | 2,440 | 1 | ,118 | 1,398 | ,918 | 2,130 |
|  | [cbs_opleidingsniveau=2,00] | ,439 | ,194 | 5,146 | 1 | ,023 | 1,552 | 1,062 | 2,269 |
|  | [cbs_opleidingsniveau=3,00] | 0^b^ | . | . | 0 | . | . | . | . |
| Mensen aanbieden om zich preventief te laten onderzoeken op darmkanker (dit is het bevolkingsonderzoek darmkanker) | Intercept | -,627 | ,280 | 5,012 | 1 | ,025 |  |  |  |
|  | leeftijd | ,000 | ,005 | ,000 | 1 | ,992 | 1,000 | ,990 | 1,010 |
|  | [geslacht=1] | ,473 | ,165 | 8,216 | 1 | ,004 | 1,606 | 1,162 | 2,219 |
|  | [geslacht=2] | 0^b^ | . | . | 0 | . | . | . | . |
|  | [cbs_opleidingsniveau=1,00] | ,241 | ,211 | 1,303 | 1 | ,254 | 1,273 | ,841 | 1,925 |
|  | [cbs_opleidingsniveau=2,00] | -,025 | ,197 | ,016 | 1 | ,899 | ,975 | ,662 | 1,436 |
|  | [cbs_opleidingsniveau=3,00] | 0^b^ | . | . | 0 | . | . | . | . |
| Het geven van voorlichting over de klachten en risicofactoren en over wat mensen zelf kunnen doen | Intercept | -,806 | ,276 | 8,502 | 1 | ,004 |  |  |  |
|  | leeftijd | ,004 | ,005 | ,727 | 1 | ,394 | 1,004 | ,994 | 1,015 |
|  | [geslacht=1] | ,326 | ,159 | 4,198 | 1 | ,040 | 1,386 | 1,014 | 1,894 |
|  | [geslacht=2] | 0^b^ | . | . | 0 | . | . | . | . |
|  | [cbs_opleidingsniveau=1,00] | ,295 | ,212 | 1,945 | 1 | ,163 | 1,343 | ,887 | 2,034 |
|  | [cbs_opleidingsniveau=2,00] | ,310 | ,193 | 2,577 | 1 | ,108 | 1,363 | ,934 | 1,990 |
|  | [cbs_opleidingsniveau=3,00] | 0^b^ | . | . | 0 | . | . | . | . |
| Het verbeteren van testen/onderzoeken om vast te stellen of iemand darmkanker heeft | Intercept | ,024 | ,248 | ,009 | 1 | ,923 |  |  |  |
|  | leeftijd | -,002 | ,005 | ,107 | 1 | ,743 | ,998 | ,989 | 1,008 |
|  | [geslacht=1] | ,183 | ,148 | 1,530 | 1 | ,216 | 1,200 | ,899 | 1,603 |
|  | [geslacht=2] | 0^b^ | . | . | 0 | . | . | . | . |
|  | [cbs_opleidingsniveau=1,00] | ,083 | ,194 | ,185 | 1 | ,667 | 1,087 | ,743 | 1,590 |
|  | [cbs_opleidingsniveau=2,00] | ,043 | ,175 | ,062 | 1 | ,804 | 1,044 | ,742 | 1,471 |
|  | [cbs_opleidingsniveau=3,00] | 0^b^ | . | . | 0 | . | . | . | . |
| a. The reference category is: Meer onderzoek doen naar de oorzaken van darmkanker. | | | | | | | | | |
| b. This parameter is set to zero because it is redundant. | | | | | | | | | |

NOMREG ANSWER.A019.01.04 (BASE=4 ORDER=ASCENDING) BY geslacht cbs_opleidingsniveau WITH leeftijd

/CRITERIA CIN(95) DELTA(0) MXITER(100) MXSTEP(5) CHKSEP(20) LCONVERGE(0) PCONVERGE(0.000001)

SINGULAR(0.00000001)

/MODEL

/STEPWISE=PIN(.05) POUT(0.1) MINEFFECT(0) RULE(SINGLE) ENTRYMETHOD(LR) REMOVALMETHOD(LR)

/INTERCEPT=INCLUDE

/PRINT=PARAMETER SUMMARY LRT CPS STEP MFI.

**Nominal Regression**

| **Notes** | | |
| --- | --- | --- |
| Output Created | | 22-JAN-2016 13:33:15 |
| Comments | |  |
| Input | Data | \\fs01-home.rivm.nl\home\doumal\Documents\RIVM_VUmc\Publieke opinie - deel A\Analyse vragenlijst 1 deel A\SPSS analyse publieke opinie vragenlijst 1\Resultaten publieke opinie vragenlijst 1_werkbestand 1.sav |
|  | Active Dataset | DataSet1 |
|  | Filter | <none> |
|  | Weight | <none> |
|  | Split File | <none> |
|  | N of Rows in Working Data File | 1679 |
| Missing Value Handling | Definition of Missing | User-defined missing values are treated as missing. |
|  | Cases Used | Statistics are based on all cases with valid data for all variables in the model. |
| Syntax | | NOMREG ANSWER.A019.01.04 (BASE=4 ORDER=ASCENDING) BY geslacht cbs_opleidingsniveau WITH leeftijd  /CRITERIA CIN(95) DELTA(0) MXITER(100) MXSTEP(5) CHKSEP(20) LCONVERGE(0) PCONVERGE(0.000001)  SINGULAR(0.00000001)  /MODEL  /STEPWISE=PIN(.05) POUT(0.1) MINEFFECT(0) RULE(SINGLE) ENTRYMETHOD(LR) REMOVALMETHOD(LR)  /INTERCEPT=INCLUDE  /PRINT=PARAMETER SUMMARY LRT CPS STEP MFI. |
| Resources | Processor Time | 00:00:00,11 |
|  | Elapsed Time | 00:00:00,13 |

| **Case Processing Summary** | | | |
| --- | --- | --- | --- |
|  | | N | Marginal Percentage |
| 23. Als u zou mogen kiezen, waar zou u dan (als eerst) het geld aan besteden? Zet de volgende onderwerpen op volgorde van belangrijkheid. Nr. 4 | Het verbeteren van de behandeling van darmkanker | 313 | 18,6% |
|  | Mensen aanbieden om zich preventief te laten onderzoeken op darmkanker (dit is het bevolkingsonderzoek darmkanker) | 277 | 16,5% |
|  | Meer onderzoek doen naar de oorzaken van darmkanker | 374 | 22,3% |
|  | Het geven van voorlichting over de klachten en risicofactoren en over wat mensen zelf kunnen doen | 309 | 18,4% |
|  | Het verbeteren van testen/onderzoeken om vast te stellen of iemand darmkanker heeft | 406 | 24,2% |
| geslacht | Man | 903 | 53,8% |
|  | Vrouw | 776 | 46,2% |
| cbs_opleidingsniveau | laag | 544 | 32,4% |
|  | mid | 681 | 40,6% |
|  | hoog | 454 | 27,0% |
| Valid | | 1679 | 100,0% |
| Missing | | 0 |  |
| Total | | 1679 |  |
| Subpopulation | | 344^a^ |  |
| a. The dependent variable has only one value observed in 69 (20,1%) subpopulations. | | | |

| **Model Fitting Information** | | | | |
| --- | --- | --- | --- | --- |
| Model | Model Fitting Criteria | Likelihood Ratio Tests | | |
|  | -2 Log Likelihood | Chi-Square | df | Sig. |
| Intercept Only | 2760,801 |  |  |  |
| Final | 2734,282 | 26,520 | 16 | ,047 |

| **Pseudo R-Square** | |
| --- | --- |
| Cox and Snell | ,016 |
| Nagelkerke | ,016 |
| McFadden | ,005 |

| **Likelihood Ratio Tests** | | | | |
| --- | --- | --- | --- | --- |
| Effect | Model Fitting Criteria | Likelihood Ratio Tests | | |
|  | -2 Log Likelihood of Reduced Model | Chi-Square | df | Sig. |
| Intercept | 2734,282^a^ | ,000 | 0 | . |
| leeftijd | 2737,022 | 2,741 | 4 | ,602 |
| geslacht | 2744,588 | 10,306 | 4 | ,036 |
| cbs_opleidingsniveau | 2745,193 | 10,912 | 8 | ,207 |
| The chi-square statistic is the difference in -2 log-likelihoods between the final model and a reduced model. The reduced model is formed by omitting an effect from the final model. The null hypothesis is that all parameters of that effect are 0. | | | | |
| a. This reduced model is equivalent to the final model because omitting the effect does not increase the degrees of freedom. | | | | |

| **Parameter Estimates** | | | | | | | | | |
| --- | --- | --- | --- | --- | --- | --- | --- | --- | --- |
| 23. Als u zou mogen kiezen, waar zou u dan (als eerst) het geld aan besteden? Zet de volgende onderwerpen op volgorde van belangrijkheid. Nr. 4^a^ | | B | Std. Error | Wald | df | Sig. | Exp(B) | 95% Confidence Interval for Exp(B) | |
|  |  |  |  |  |  |  |  | Lower Bound | Upper Bound |
| Het verbeteren van de behandeling van darmkanker | Intercept | ,026 | ,297 | ,008 | 1 | ,929 |  |  |  |
|  | leeftijd | ,001 | ,005 | ,025 | 1 | ,875 | 1,001 | ,990 | 1,011 |
|  | [geslacht=1] | -,233 | ,166 | 1,979 | 1 | ,159 | ,792 | ,572 | 1,096 |
|  | [geslacht=2] | 0^b^ | . | . | 0 | . | . | . | . |
|  | [cbs_opleidingsniveau=1,00] | ,040 | ,227 | ,031 | 1 | ,860 | 1,041 | ,667 | 1,624 |
|  | [cbs_opleidingsniveau=2,00] | ,130 | ,208 | ,390 | 1 | ,532 | 1,139 | ,758 | 1,711 |
|  | [cbs_opleidingsniveau=3,00] | 0^b^ | . | . | 0 | . | . | . | . |
| Mensen aanbieden om zich preventief te laten onderzoeken op darmkanker (dit is het bevolkingsonderzoek darmkanker) | Intercept | ,179 | ,301 | ,356 | 1 | ,551 |  |  |  |
|  | leeftijd | -,004 | ,006 | ,653 | 1 | ,419 | ,996 | ,985 | 1,006 |
|  | [geslacht=1] | ,147 | ,173 | ,722 | 1 | ,395 | 1,158 | ,825 | 1,626 |
|  | [geslacht=2] | 0^b^ | . | . | 0 | . | . | . | . |
|  | [cbs_opleidingsniveau=1,00] | -,054 | ,223 | ,059 | 1 | ,808 | ,947 | ,612 | 1,467 |
|  | [cbs_opleidingsniveau=2,00] | -,335 | ,211 | 2,522 | 1 | ,112 | ,716 | ,473 | 1,081 |
|  | [cbs_opleidingsniveau=3,00] | 0^b^ | . | . | 0 | . | . | . | . |
| Meer onderzoek doen naar de oorzaken van darmkanker | Intercept | ,806 | ,276 | 8,502 | 1 | ,004 |  |  |  |
|  | leeftijd | -,004 | ,005 | ,727 | 1 | ,394 | ,996 | ,986 | 1,006 |
|  | [geslacht=1] | -,326 | ,159 | 4,198 | 1 | ,040 | ,722 | ,528 | ,986 |
|  | [geslacht=2] | 0^b^ | . | . | 0 | . | . | . | . |
|  | [cbs_opleidingsniveau=1,00] | -,295 | ,212 | 1,945 | 1 | ,163 | ,744 | ,492 | 1,127 |
|  | [cbs_opleidingsniveau=2,00] | -,310 | ,193 | 2,577 | 1 | ,108 | ,734 | ,503 | 1,071 |
|  | [cbs_opleidingsniveau=3,00] | 0^b^ | . | . | 0 | . | . | . | . |
| Het verbeteren van testen/onderzoeken om vast te stellen of iemand darmkanker heeft | Intercept | ,830 | ,272 | 9,299 | 1 | ,002 |  |  |  |
|  | leeftijd | -,006 | ,005 | 1,403 | 1 | ,236 | ,994 | ,984 | 1,004 |
|  | [geslacht=1] | -,144 | ,156 | ,845 | 1 | ,358 | ,866 | ,637 | 1,177 |
|  | [geslacht=2] | 0^b^ | . | . | 0 | . | . | . | . |
|  | [cbs_opleidingsniveau=1,00] | -,212 | ,207 | 1,043 | 1 | ,307 | ,809 | ,539 | 1,215 |
|  | [cbs_opleidingsniveau=2,00] | -,266 | ,190 | 1,962 | 1 | ,161 | ,766 | ,528 | 1,112 |
|  | [cbs_opleidingsniveau=3,00] | 0^b^ | . | . | 0 | . | . | . | . |
| a. The reference category is: Het geven van voorlichting over de klachten en risicofactoren en over wat mensen zelf kunnen doen. | | | | | | | | | |
| b. This parameter is set to zero because it is redundant. | | | | | | | | | |

NOMREG ANSWER.A019.01.05 (BASE=LAST ORDER=ASCENDING) BY geslacht cbs_opleidingsniveau WITH leeftijd

/CRITERIA CIN(95) DELTA(0) MXITER(100) MXSTEP(5) CHKSEP(20) LCONVERGE(0) PCONVERGE(0.000001)

SINGULAR(0.00000001)

/MODEL

/STEPWISE=PIN(.05) POUT(0.1) MINEFFECT(0) RULE(SINGLE) ENTRYMETHOD(LR) REMOVALMETHOD(LR)

/INTERCEPT=INCLUDE

/PRINT=PARAMETER SUMMARY LRT CPS STEP MFI.

**Nominal Regression**

| **Notes** | | |
| --- | --- | --- |
| Output Created | | 22-JAN-2016 13:35:55 |
| Comments | |  |
| Input | Data | \\fs01-home.rivm.nl\home\doumal\Documents\RIVM_VUmc\Publieke opinie - deel A\Analyse vragenlijst 1 deel A\SPSS analyse publieke opinie vragenlijst 1\Resultaten publieke opinie vragenlijst 1_werkbestand 1.sav |
|  | Active Dataset | DataSet1 |
|  | Filter | <none> |
|  | Weight | <none> |
|  | Split File | <none> |
|  | N of Rows in Working Data File | 1679 |
| Missing Value Handling | Definition of Missing | User-defined missing values are treated as missing. |
|  | Cases Used | Statistics are based on all cases with valid data for all variables in the model. |
| Syntax | | NOMREG ANSWER.A019.01.05 (BASE=LAST ORDER=ASCENDING) BY geslacht cbs_opleidingsniveau WITH leeftijd  /CRITERIA CIN(95) DELTA(0) MXITER(100) MXSTEP(5) CHKSEP(20) LCONVERGE(0) PCONVERGE(0.000001)  SINGULAR(0.00000001)  /MODEL  /STEPWISE=PIN(.05) POUT(0.1) MINEFFECT(0) RULE(SINGLE) ENTRYMETHOD(LR) REMOVALMETHOD(LR)  /INTERCEPT=INCLUDE  /PRINT=PARAMETER SUMMARY LRT CPS STEP MFI. |
| Resources | Processor Time | 00:00:00,11 |
|  | Elapsed Time | 00:00:00,12 |

| **Case Processing Summary** | | | |
| --- | --- | --- | --- |
|  | | N | Marginal Percentage |
| 23. Als u zou mogen kiezen, waar zou u dan (als eerst) het geld aan besteden? Zet de volgende onderwerpen op volgorde van belangrijkheid. Nr. 5 | Het verbeteren van de behandeling van darmkanker | 273 | 16,3% |
|  | Mensen aanbieden om zich preventief te laten onderzoeken op darmkanker (dit is het bevolkingsonderzoek darmkanker) | 315 | 18,8% |
|  | Meer onderzoek doen naar de oorzaken van darmkanker | 259 | 15,4% |
|  | Het geven van voorlichting over de klachten en risicofactoren en over wat mensen zelf kunnen doen | 439 | 26,1% |
|  | Het verbeteren van testen/onderzoeken om vast te stellen of iemand darmkanker heeft | 393 | 23,4% |
| geslacht | Man | 903 | 53,8% |
|  | Vrouw | 776 | 46,2% |
| cbs_opleidingsniveau | laag | 544 | 32,4% |
|  | mid | 681 | 40,6% |
|  | hoog | 454 | 27,0% |
| Valid | | 1679 | 100,0% |
| Missing | | 0 |  |
| Total | | 1679 |  |
| Subpopulation | | 344^a^ |  |
| a. The dependent variable has only one value observed in 72 (20,9%) subpopulations. | | | |

| **Model Fitting Information** | | | | |
| --- | --- | --- | --- | --- |
| Model | Model Fitting Criteria | Likelihood Ratio Tests | | |
|  | -2 Log Likelihood | Chi-Square | df | Sig. |
| Intercept Only | 2739,562 |  |  |  |
| Final | 2707,744 | 31,818 | 16 | ,011 |

| **Pseudo R-Square** | |
| --- | --- |
| Cox and Snell | ,019 |
| Nagelkerke | ,020 |
| McFadden | ,006 |

| **Likelihood Ratio Tests** | | | | |
| --- | --- | --- | --- | --- |
| Effect | Model Fitting Criteria | Likelihood Ratio Tests | | |
|  | -2 Log Likelihood of Reduced Model | Chi-Square | df | Sig. |
| Intercept | 2707,744^a^ | ,000 | 0 | . |
| leeftijd | 2723,157 | 15,413 | 4 | ,004 |
| geslacht | 2711,038 | 3,293 | 4 | ,510 |
| cbs_opleidingsniveau | 2722,733 | 14,988 | 8 | ,059 |
| The chi-square statistic is the difference in -2 log-likelihoods between the final model and a reduced model. The reduced model is formed by omitting an effect from the final model. The null hypothesis is that all parameters of that effect are 0. | | | | |
| a. This reduced model is equivalent to the final model because omitting the effect does not increase the degrees of freedom. | | | | |

| **Parameter Estimates** | | | | | | | | | |
| --- | --- | --- | --- | --- | --- | --- | --- | --- | --- |
| 23. Als u zou mogen kiezen, waar zou u dan (als eerst) het geld aan besteden? Zet de volgende onderwerpen op volgorde van belangrijkheid. Nr. 5^a^ | | B | Std. Error | Wald | df | Sig. | Exp(B) | 95% Confidence Interval for Exp(B) | |
|  |  |  |  |  |  |  |  | Lower Bound | Upper Bound |
| Het verbeteren van de behandeling van darmkanker | Intercept | -,643 | ,288 | 4,997 | 1 | ,025 |  |  |  |
|  | leeftijd | ,007 | ,005 | 1,649 | 1 | ,199 | 1,007 | ,996 | 1,018 |
|  | [geslacht=1] | ,210 | ,164 | 1,633 | 1 | ,201 | 1,234 | ,894 | 1,703 |
|  | [geslacht=2] | 0^b^ | . | . | 0 | . | . | . | . |
|  | [cbs_opleidingsniveau=1,00] | -,071 | ,210 | ,116 | 1 | ,734 | ,931 | ,617 | 1,405 |
|  | [cbs_opleidingsniveau=2,00] | -,447 | ,196 | 5,180 | 1 | ,023 | ,640 | ,435 | ,940 |
|  | [cbs_opleidingsniveau=3,00] | 0^b^ | . | . | 0 | . | . | . | . |
| Mensen aanbieden om zich preventief te laten onderzoeken op darmkanker (dit is het bevolkingsonderzoek darmkanker) | Intercept | ,118 | ,267 | ,197 | 1 | ,657 |  |  |  |
|  | leeftijd | -,008 | ,005 | 2,306 | 1 | ,129 | ,992 | ,982 | 1,002 |
|  | [geslacht=1] | ,119 | ,156 | ,586 | 1 | ,444 | 1,127 | ,830 | 1,530 |
|  | [geslacht=2] | 0^b^ | . | . | 0 | . | . | . | . |
|  | [cbs_opleidingsniveau=1,00] | ,037 | ,210 | ,032 | 1 | ,859 | 1,038 | ,688 | 1,565 |
|  | [cbs_opleidingsniveau=2,00] | -,065 | ,186 | ,122 | 1 | ,727 | ,937 | ,652 | 1,348 |
|  | [cbs_opleidingsniveau=3,00] | 0^b^ | . | . | 0 | . | . | . | . |
| Meer onderzoek doen naar de oorzaken van darmkanker | Intercept | -,230 | ,286 | ,648 | 1 | ,421 |  |  |  |
|  | leeftijd | -,008 | ,005 | 1,967 | 1 | ,161 | ,992 | ,982 | 1,003 |
|  | [geslacht=1] | ,175 | ,165 | 1,121 | 1 | ,290 | 1,191 | ,862 | 1,646 |
|  | [geslacht=2] | 0^b^ | . | . | 0 | . | . | . | . |
|  | [cbs_opleidingsniveau=1,00] | ,323 | ,221 | 2,141 | 1 | ,143 | 1,381 | ,896 | 2,128 |
|  | [cbs_opleidingsniveau=2,00] | -,009 | ,202 | ,002 | 1 | ,965 | ,991 | ,667 | 1,472 |
|  | [cbs_opleidingsniveau=3,00] | 0^b^ | . | . | 0 | . | . | . | . |
| Het geven van voorlichting over de klachten en risicofactoren en over wat mensen zelf kunnen doen | Intercept | ,367 | ,248 | 2,188 | 1 | ,139 |  |  |  |
|  | leeftijd | -,011 | ,005 | 6,016 | 1 | ,014 | ,989 | ,980 | ,998 |
|  | [geslacht=1] | ,243 | ,144 | 2,870 | 1 | ,090 | 1,275 | ,963 | 1,689 |
|  | [geslacht=2] | 0^b^ | . | . | 0 | . | . | . | . |
|  | [cbs_opleidingsniveau=1,00] | ,391 | ,194 | 4,052 | 1 | ,044 | 1,479 | 1,010 | 2,165 |
|  | [cbs_opleidingsniveau=2,00] | ,142 | ,175 | ,661 | 1 | ,416 | 1,153 | ,818 | 1,625 |
|  | [cbs_opleidingsniveau=3,00] | 0^b^ | . | . | 0 | . | . | . | . |
| a. The reference category is: Het verbeteren van testen/onderzoeken om vast te stellen of iemand darmkanker heeft. | | | | | | | | | |
| b. This parameter is set to zero because it is redundant. | | | | | | | | | |

NOMREG ANSWER.A019.01.05 (BASE=3 ORDER=ASCENDING) BY geslacht cbs_opleidingsniveau WITH leeftijd

/CRITERIA CIN(95) DELTA(0) MXITER(100) MXSTEP(5) CHKSEP(20) LCONVERGE(0) PCONVERGE(0.000001)

SINGULAR(0.00000001)

/MODEL

/STEPWISE=PIN(.05) POUT(0.1) MINEFFECT(0) RULE(SINGLE) ENTRYMETHOD(LR) REMOVALMETHOD(LR)

/INTERCEPT=INCLUDE

/PRINT=PARAMETER SUMMARY LRT CPS STEP MFI.

**Nominal Regression**

| **Notes** | | |
| --- | --- | --- |
| Output Created | | 22-JAN-2016 13:36:37 |
| Comments | |  |
| Input | Data | \\fs01-home.rivm.nl\home\doumal\Documents\RIVM_VUmc\Publieke opinie - deel A\Analyse vragenlijst 1 deel A\SPSS analyse publieke opinie vragenlijst 1\Resultaten publieke opinie vragenlijst 1_werkbestand 1.sav |
|  | Active Dataset | DataSet1 |
|  | Filter | <none> |
|  | Weight | <none> |
|  | Split File | <none> |
|  | N of Rows in Working Data File | 1679 |
| Missing Value Handling | Definition of Missing | User-defined missing values are treated as missing. |
|  | Cases Used | Statistics are based on all cases with valid data for all variables in the model. |
| Syntax | | NOMREG ANSWER.A019.01.05 (BASE=3 ORDER=ASCENDING) BY geslacht cbs_opleidingsniveau WITH leeftijd  /CRITERIA CIN(95) DELTA(0) MXITER(100) MXSTEP(5) CHKSEP(20) LCONVERGE(0) PCONVERGE(0.000001)  SINGULAR(0.00000001)  /MODEL  /STEPWISE=PIN(.05) POUT(0.1) MINEFFECT(0) RULE(SINGLE) ENTRYMETHOD(LR) REMOVALMETHOD(LR)  /INTERCEPT=INCLUDE  /PRINT=PARAMETER SUMMARY LRT CPS STEP MFI. |
| Resources | Processor Time | 00:00:00,11 |
|  | Elapsed Time | 00:00:00,12 |

| **Case Processing Summary** | | | |
| --- | --- | --- | --- |
|  | | N | Marginal Percentage |
| 23. Als u zou mogen kiezen, waar zou u dan (als eerst) het geld aan besteden? Zet de volgende onderwerpen op volgorde van belangrijkheid. Nr. 5 | Het verbeteren van de behandeling van darmkanker | 273 | 16,3% |
|  | Mensen aanbieden om zich preventief te laten onderzoeken op darmkanker (dit is het bevolkingsonderzoek darmkanker) | 315 | 18,8% |
|  | Meer onderzoek doen naar de oorzaken van darmkanker | 259 | 15,4% |
|  | Het geven van voorlichting over de klachten en risicofactoren en over wat mensen zelf kunnen doen | 439 | 26,1% |
|  | Het verbeteren van testen/onderzoeken om vast te stellen of iemand darmkanker heeft | 393 | 23,4% |
| geslacht | Man | 903 | 53,8% |
|  | Vrouw | 776 | 46,2% |
| cbs_opleidingsniveau | laag | 544 | 32,4% |
|  | mid | 681 | 40,6% |
|  | hoog | 454 | 27,0% |
| Valid | | 1679 | 100,0% |
| Missing | | 0 |  |
| Total | | 1679 |  |
| Subpopulation | | 344^a^ |  |
| a. The dependent variable has only one value observed in 72 (20,9%) subpopulations. | | | |

| **Model Fitting Information** | | | | |
| --- | --- | --- | --- | --- |
| Model | Model Fitting Criteria | Likelihood Ratio Tests | | |
|  | -2 Log Likelihood | Chi-Square | df | Sig. |
| Intercept Only | 2739,562 |  |  |  |
| Final | 2707,744 | 31,818 | 16 | ,011 |

| **Pseudo R-Square** | |
| --- | --- |
| Cox and Snell | ,019 |
| Nagelkerke | ,020 |
| McFadden | ,006 |

| **Likelihood Ratio Tests** | | | | |
| --- | --- | --- | --- | --- |
| Effect | Model Fitting Criteria | Likelihood Ratio Tests | | |
|  | -2 Log Likelihood of Reduced Model | Chi-Square | df | Sig. |
| Intercept | 2707,744^a^ | ,000 | 0 | . |
| leeftijd | 2723,157 | 15,413 | 4 | ,004 |
| geslacht | 2711,038 | 3,293 | 4 | ,510 |
| cbs_opleidingsniveau | 2722,733 | 14,988 | 8 | ,059 |
| The chi-square statistic is the difference in -2 log-likelihoods between the final model and a reduced model. The reduced model is formed by omitting an effect from the final model. The null hypothesis is that all parameters of that effect are 0. | | | | |
| a. This reduced model is equivalent to the final model because omitting the effect does not increase the degrees of freedom. | | | | |

| **Parameter Estimates** | | | | | | | | | |
| --- | --- | --- | --- | --- | --- | --- | --- | --- | --- |
| 23. Als u zou mogen kiezen, waar zou u dan (als eerst) het geld aan besteden? Zet de volgende onderwerpen op volgorde van belangrijkheid. Nr. 5^a^ | | B | Std. Error | Wald | df | Sig. | Exp(B) | 95% Confidence Interval for Exp(B) | |
|  |  |  |  |  |  |  |  | Lower Bound | Upper Bound |
| Het verbeteren van de behandeling van darmkanker | Intercept | -,413 | ,314 | 1,724 | 1 | ,189 |  |  |  |
|  | leeftijd | ,014 | ,006 | 6,028 | 1 | ,014 | 1,015 | 1,003 | 1,026 |
|  | [geslacht=1] | ,035 | ,180 | ,038 | 1 | ,845 | 1,036 | ,727 | 1,475 |
|  | [geslacht=2] | 0^b^ | . | . | 0 | . | . | . | . |
|  | [cbs_opleidingsniveau=1,00] | -,394 | ,231 | 2,921 | 1 | ,087 | ,674 | ,429 | 1,060 |
|  | [cbs_opleidingsniveau=2,00] | -,438 | ,220 | 3,965 | 1 | ,046 | ,645 | ,419 | ,993 |
|  | [cbs_opleidingsniveau=3,00] | 0^b^ | . | . | 0 | . | . | . | . |
| Mensen aanbieden om zich preventief te laten onderzoeken op darmkanker (dit is het bevolkingsonderzoek darmkanker) | Intercept | ,348 | ,295 | 1,394 | 1 | ,238 |  |  |  |
|  | leeftijd | ,000 | ,006 | ,001 | 1 | ,976 | 1,000 | ,989 | 1,011 |
|  | [geslacht=1] | -,055 | ,173 | ,103 | 1 | ,748 | ,946 | ,674 | 1,327 |
|  | [geslacht=2] | 0^b^ | . | . | 0 | . | . | . | . |
|  | [cbs_opleidingsniveau=1,00] | -,285 | ,230 | 1,545 | 1 | ,214 | ,752 | ,479 | 1,179 |
|  | [cbs_opleidingsniveau=2,00] | -,056 | ,210 | ,071 | 1 | ,790 | ,946 | ,627 | 1,427 |
|  | [cbs_opleidingsniveau=3,00] | 0^b^ | . | . | 0 | . | . | . | . |
| Het geven van voorlichting over de klachten en risicofactoren en over wat mensen zelf kunnen doen | Intercept | ,597 | ,278 | 4,602 | 1 | ,032 |  |  |  |
|  | leeftijd | -,004 | ,005 | ,558 | 1 | ,455 | ,996 | ,986 | 1,006 |
|  | [geslacht=1] | ,068 | ,161 | ,179 | 1 | ,672 | 1,071 | ,780 | 1,469 |
|  | [geslacht=2] | 0^b^ | . | . | 0 | . | . | . | . |
|  | [cbs_opleidingsniveau=1,00] | ,069 | ,216 | ,101 | 1 | ,750 | 1,071 | ,702 | 1,634 |
|  | [cbs_opleidingsniveau=2,00] | ,151 | ,201 | ,568 | 1 | ,451 | 1,163 | ,785 | 1,723 |
|  | [cbs_opleidingsniveau=3,00] | 0^b^ | . | . | 0 | . | . | . | . |
| Het verbeteren van testen/onderzoeken om vast te stellen of iemand darmkanker heeft | Intercept | ,230 | ,286 | ,648 | 1 | ,421 |  |  |  |
|  | leeftijd | ,008 | ,005 | 1,967 | 1 | ,161 | 1,008 | ,997 | 1,018 |
|  | [geslacht=1] | -,175 | ,165 | 1,121 | 1 | ,290 | ,840 | ,607 | 1,160 |
|  | [geslacht=2] | 0^b^ | . | . | 0 | . | . | . | . |
|  | [cbs_opleidingsniveau=1,00] | -,323 | ,221 | 2,141 | 1 | ,143 | ,724 | ,470 | 1,116 |
|  | [cbs_opleidingsniveau=2,00] | ,009 | ,202 | ,002 | 1 | ,965 | 1,009 | ,679 | 1,498 |
|  | [cbs_opleidingsniveau=3,00] | 0^b^ | . | . | 0 | . | . | . | . |
| a. The reference category is: Meer onderzoek doen naar de oorzaken van darmkanker. | | | | | | | | | |
| b. This parameter is set to zero because it is redundant. | | | | | | | | | |

NOMREG ANSWER.A019.01.05 (BASE=4 ORDER=ASCENDING) BY geslacht cbs_opleidingsniveau WITH leeftijd

/CRITERIA CIN(95) DELTA(0) MXITER(100) MXSTEP(5) CHKSEP(20) LCONVERGE(0) PCONVERGE(0.000001)

SINGULAR(0.00000001)

/MODEL

/STEPWISE=PIN(.05) POUT(0.1) MINEFFECT(0) RULE(SINGLE) ENTRYMETHOD(LR) REMOVALMETHOD(LR)

/INTERCEPT=INCLUDE

/PRINT=PARAMETER SUMMARY LRT CPS STEP MFI.

**Nominal Regression**

| **Notes** | | |
| --- | --- | --- |
| Output Created | | 22-JAN-2016 13:37:14 |
| Comments | |  |
| Input | Data | \\fs01-home.rivm.nl\home\doumal\Documents\RIVM_VUmc\Publieke opinie - deel A\Analyse vragenlijst 1 deel A\SPSS analyse publieke opinie vragenlijst 1\Resultaten publieke opinie vragenlijst 1_werkbestand 1.sav |
|  | Active Dataset | DataSet1 |
|  | Filter | <none> |
|  | Weight | <none> |
|  | Split File | <none> |
|  | N of Rows in Working Data File | 1679 |
| Missing Value Handling | Definition of Missing | User-defined missing values are treated as missing. |
|  | Cases Used | Statistics are based on all cases with valid data for all variables in the model. |
| Syntax | | NOMREG ANSWER.A019.01.05 (BASE=4 ORDER=ASCENDING) BY geslacht cbs_opleidingsniveau WITH leeftijd  /CRITERIA CIN(95) DELTA(0) MXITER(100) MXSTEP(5) CHKSEP(20) LCONVERGE(0) PCONVERGE(0.000001)  SINGULAR(0.00000001)  /MODEL  /STEPWISE=PIN(.05) POUT(0.1) MINEFFECT(0) RULE(SINGLE) ENTRYMETHOD(LR) REMOVALMETHOD(LR)  /INTERCEPT=INCLUDE  /PRINT=PARAMETER SUMMARY LRT CPS STEP MFI. |
| Resources | Processor Time | 00:00:00,14 |
|  | Elapsed Time | 00:00:00,14 |

| **Case Processing Summary** | | | |
| --- | --- | --- | --- |
|  | | N | Marginal Percentage |
| 23. Als u zou mogen kiezen, waar zou u dan (als eerst) het geld aan besteden? Zet de volgende onderwerpen op volgorde van belangrijkheid. Nr. 5 | Het verbeteren van de behandeling van darmkanker | 273 | 16,3% |
|  | Mensen aanbieden om zich preventief te laten onderzoeken op darmkanker (dit is het bevolkingsonderzoek darmkanker) | 315 | 18,8% |
|  | Meer onderzoek doen naar de oorzaken van darmkanker | 259 | 15,4% |
|  | Het geven van voorlichting over de klachten en risicofactoren en over wat mensen zelf kunnen doen | 439 | 26,1% |
|  | Het verbeteren van testen/onderzoeken om vast te stellen of iemand darmkanker heeft | 393 | 23,4% |
| geslacht | Man | 903 | 53,8% |
|  | Vrouw | 776 | 46,2% |
| cbs_opleidingsniveau | laag | 544 | 32,4% |
|  | mid | 681 | 40,6% |
|  | hoog | 454 | 27,0% |
| Valid | | 1679 | 100,0% |
| Missing | | 0 |  |
| Total | | 1679 |  |
| Subpopulation | | 344^a^ |  |
| a. The dependent variable has only one value observed in 72 (20,9%) subpopulations. | | | |

| **Model Fitting Information** | | | | |
| --- | --- | --- | --- | --- |
| Model | Model Fitting Criteria | Likelihood Ratio Tests | | |
|  | -2 Log Likelihood | Chi-Square | df | Sig. |
| Intercept Only | 2739,562 |  |  |  |
| Final | 2707,744 | 31,818 | 16 | ,011 |

| **Pseudo R-Square** | |
| --- | --- |
| Cox and Snell | ,019 |
| Nagelkerke | ,020 |
| McFadden | ,006 |

| **Likelihood Ratio Tests** | | | | |
| --- | --- | --- | --- | --- |
| Effect | Model Fitting Criteria | Likelihood Ratio Tests | | |
|  | -2 Log Likelihood of Reduced Model | Chi-Square | df | Sig. |
| Intercept | 2707,744^a^ | ,000 | 0 | . |
| leeftijd | 2723,157 | 15,413 | 4 | ,004 |
| geslacht | 2711,038 | 3,293 | 4 | ,510 |
| cbs_opleidingsniveau | 2722,733 | 14,988 | 8 | ,059 |
| The chi-square statistic is the difference in -2 log-likelihoods between the final model and a reduced model. The reduced model is formed by omitting an effect from the final model. The null hypothesis is that all parameters of that effect are 0. | | | | |
| a. This reduced model is equivalent to the final model because omitting the effect does not increase the degrees of freedom. | | | | |

| **Parameter Estimates** | | | | | | | | | |
| --- | --- | --- | --- | --- | --- | --- | --- | --- | --- |
| 23. Als u zou mogen kiezen, waar zou u dan (als eerst) het geld aan besteden? Zet de volgende onderwerpen op volgorde van belangrijkheid. Nr. 5^a^ | | B | Std. Error | Wald | df | Sig. | Exp(B) | 95% Confidence Interval for Exp(B) | |
|  |  |  |  |  |  |  |  | Lower Bound | Upper Bound |
| Het verbeteren van de behandeling van darmkanker | Intercept | -1,010 | ,281 | 12,931 | 1 | ,000 |  |  |  |
|  | leeftijd | ,018 | ,005 | 12,207 | 1 | ,000 | 1,019 | 1,008 | 1,029 |
|  | [geslacht=1] | -,033 | ,161 | ,042 | 1 | ,838 | ,968 | ,705 | 1,327 |
|  | [geslacht=2] | 0^b^ | . | . | 0 | . | . | . | . |
|  | [cbs_opleidingsniveau=1,00] | -,463 | ,206 | 5,051 | 1 | ,025 | ,630 | ,420 | ,943 |
|  | [cbs_opleidingsniveau=2,00] | -,589 | ,196 | 9,059 | 1 | ,003 | ,555 | ,378 | ,814 |
|  | [cbs_opleidingsniveau=3,00] | 0^b^ | . | . | 0 | . | . | . | . |
| Mensen aanbieden om zich preventief te laten onderzoeken op darmkanker (dit is het bevolkingsonderzoek darmkanker) | Intercept | -,249 | ,259 | ,926 | 1 | ,336 |  |  |  |
|  | leeftijd | ,004 | ,005 | ,575 | 1 | ,448 | 1,004 | ,994 | 1,013 |
|  | [geslacht=1] | -,124 | ,152 | ,661 | 1 | ,416 | ,884 | ,656 | 1,191 |
|  | [geslacht=2] | 0^b^ | . | . | 0 | . | . | . | . |
|  | [cbs_opleidingsniveau=1,00] | -,354 | ,205 | 2,997 | 1 | ,083 | ,702 | ,470 | 1,048 |
|  | [cbs_opleidingsniveau=2,00] | -,207 | ,184 | 1,263 | 1 | ,261 | ,813 | ,567 | 1,167 |
|  | [cbs_opleidingsniveau=3,00] | 0^b^ | . | . | 0 | . | . | . | . |
| Meer onderzoek doen naar de oorzaken van darmkanker | Intercept | -,597 | ,278 | 4,602 | 1 | ,032 |  |  |  |
|  | leeftijd | ,004 | ,005 | ,558 | 1 | ,455 | 1,004 | ,994 | 1,014 |
|  | [geslacht=1] | -,068 | ,161 | ,179 | 1 | ,672 | ,934 | ,681 | 1,281 |
|  | [geslacht=2] | 0^b^ | . | . | 0 | . | . | . | . |
|  | [cbs_opleidingsniveau=1,00] | -,069 | ,216 | ,101 | 1 | ,750 | ,934 | ,612 | 1,425 |
|  | [cbs_opleidingsniveau=2,00] | -,151 | ,201 | ,568 | 1 | ,451 | ,860 | ,580 | 1,274 |
|  | [cbs_opleidingsniveau=3,00] | 0^b^ | . | . | 0 | . | . | . | . |
| Het verbeteren van testen/onderzoeken om vast te stellen of iemand darmkanker heeft | Intercept | -,367 | ,248 | 2,188 | 1 | ,139 |  |  |  |
|  | leeftijd | ,011 | ,005 | 6,016 | 1 | ,014 | 1,012 | 1,002 | 1,021 |
|  | [geslacht=1] | -,243 | ,144 | 2,870 | 1 | ,090 | ,784 | ,592 | 1,039 |
|  | [geslacht=2] | 0^b^ | . | . | 0 | . | . | . | . |
|  | [cbs_opleidingsniveau=1,00] | -,391 | ,194 | 4,052 | 1 | ,044 | ,676 | ,462 | ,990 |
|  | [cbs_opleidingsniveau=2,00] | -,142 | ,175 | ,661 | 1 | ,416 | ,867 | ,615 | 1,222 |
|  | [cbs_opleidingsniveau=3,00] | 0^b^ | . | . | 0 | . | . | . | . |
| a. The reference category is: Het geven van voorlichting over de klachten en risicofactoren en over wat mensen zelf kunnen doen. | | | | | | | | | |
| b. This parameter is set to zero because it is redundant. | | | | | | | | | |
