# Supplementary material for: The Dutch public are positive about the colorectal cancer-screening programme, but is this a well-informed opinion?
Source: BMC Public Health. 2016 Nov 29;16:1208. doi: 10.1186/s12889-016-3870-7 (PMC5129673; doi:10.1186/s12889-016-3870-7)
Supplement: Additional file 3: — Correlations key components_spss output. (DOCX 58 kb) [file 12889_2016_3870_MOESM3_ESM.docx]

| **Correlations** |  |  |  |  |  |  |  |  |  |  |  |  |  |  |  |  |  |  |  |  |
| --- | --- | --- | --- | --- | --- | --- | --- | --- | --- | --- | --- | --- | --- | --- | --- | --- | --- | --- | --- | --- |
|  | | | 3. Wilt u uit dit rijtje aangeven over welke onderwerpen u wél gehoord heeft. Er zijn meerdere antwoorden mogelijk. algemene informatie over darmkanker en het bevolkingsonderzoek darmkanker | 3. Wilt u uit dit rijtje aangeven over welke onderwerpen u wél gehoord heeft. Er zijn meerdere antwoorden mogelijk. informatie over darmkanker en het bevolkingsonderzoek darmkanker in cijfers | 3. Wilt u uit dit rijtje aangeven over welke onderwerpen u wél gehoord heeft. Er zijn meerdere antwoorden mogelijk. wat de voordelen van het bevolkingsonderzoek darmkanker zijn | 3. Wilt u uit dit rijtje aangeven over welke onderwerpen u wél gehoord heeft. Er zijn meerdere antwoorden mogelijk. wat de nadelen en risico's van het bevolkingsonderzoek darmkanker zijn | 3. Wilt u uit dit rijtje aangeven over welke onderwerpen u wél gehoord heeft. Er zijn meerdere antwoorden mogelijk. welke kosten er kunnen zijn voor deelnemers aan het bevolkingsonderzoek darmkanker | 3. Wilt u uit dit rijtje aangeven over welke onderwerpen u wél gehoord heeft. Er zijn meerdere antwoorden mogelijk. dat het bevolkingsonderzoek darmkanker door de overheid wordt aangeboden | 3. Wilt u uit dit rijtje aangeven over welke onderwerpen u wél gehoord heeft. Er zijn meerdere antwoorden mogelijk. dat mensen zelf beslissen of ze wel of niet mee willen doen aan het bevolkingsonderzoek darmkanker | 3. Wilt u uit dit rijtje aangeven over welke onderwerpen u wél gehoord heeft. Er zijn meerdere antwoorden mogelijk. dat mensen een ontlastingstest thuisgestuurd krijgen en er gekeken wordt of er in hun ontlasting bloed zit | 3. Wilt u uit dit rijtje aangeven over welke onderwerpen u wél gehoord heeft. Er zijn meerdere antwoorden mogelijk. informatie over de kwaliteit van de ontlastingstest | 3. Wilt u uit dit rijtje aangeven over welke onderwerpen u wél gehoord heeft. Er zijn meerdere antwoorden mogelijk. dat mensen eventueel een vervolgonderzoek (coloscopie) moeten laten doen en wat er dan gebeurt | 3. Wilt u uit dit rijtje aangeven over welke onderwerpen u wél gehoord heeft. Er zijn meerdere antwoorden mogelijk. wachttijden voor een coloscopie | 3. Wilt u uit dit rijtje aangeven over welke onderwerpen u wél gehoord heeft. Er zijn meerdere antwoorden mogelijk. geen van deze | 25. Vindt u het, alles bij elkaar genomen, goed dat in Nederland het bevolkingsonderzoek darmkanker wordt aangeboden? | steun_gedwkeuze_schaal_hercod_1tot5 | collectieve_attitude | persoonlijke_attitude | schaal_subjectievekennis | kennisvragenjuist |
| 3. Wilt u uit dit rijtje aangeven over welke onderwerpen u wél gehoord heeft. Er zijn meerdere antwoorden mogelijk. algemene informatie over darmkanker en het bevolkingsonderzoek darmkanker | | Pearson Correlation | 1 | ,203^**^ | ,281^**^ | ,197^**^ | ,137^**^ | ,154^**^ | ,200^**^ | ,212^**^ | ,145^**^ | ,254^**^ | ,065^*^ | -,409^**^ | ,108^**^ | ,074^**^ | ,107^**^ | ,115^**^ | ,232^**^ | ,086^**^ |
|  |  | Sig. (2-tailed) |  | ,000 | ,000 | ,000 | ,000 | ,000 | ,000 | ,000 | ,000 | ,000 | ,016 | ,000 | ,000 | ,006 | ,000 | ,000 | ,000 | ,002 |
|  |  | N | 1348 | 1348 | 1348 | 1348 | 1348 | 1348 | 1348 | 1348 | 1348 | 1348 | 1348 | 1348 | 1348 | 1348 | 1348 | 1348 | 1348 | 1348 |
| 3. Wilt u uit dit rijtje aangeven over welke onderwerpen u wél gehoord heeft. Er zijn meerdere antwoorden mogelijk. informatie over darmkanker en het bevolkingsonderzoek darmkanker in cijfers | | Pearson Correlation | ,203^**^ | 1 | ,223^**^ | ,290^**^ | ,280^**^ | ,183^**^ | ,167^**^ | ,185^**^ | ,350^**^ | ,272^**^ | ,227^**^ | -,117^**^ | ,073^**^ | ,015 | ,064^*^ | ,088^**^ | ,260^**^ | ,063^*^ |
|  |  | Sig. (2-tailed) | ,000 |  | ,000 | ,000 | ,000 | ,000 | ,000 | ,000 | ,000 | ,000 | ,000 | ,000 | ,007 | ,578 | ,019 | ,001 | ,000 | ,021 |
|  |  | N | 1348 | 1348 | 1348 | 1348 | 1348 | 1348 | 1348 | 1348 | 1348 | 1348 | 1348 | 1348 | 1348 | 1348 | 1348 | 1348 | 1348 | 1348 |
| 3. Wilt u uit dit rijtje aangeven over welke onderwerpen u wél gehoord heeft. Er zijn meerdere antwoorden mogelijk. wat de voordelen van het bevolkingsonderzoek darmkanker zijn | | Pearson Correlation | ,281^**^ | ,223^**^ | 1 | ,399^**^ | ,218^**^ | ,305^**^ | ,327^**^ | ,350^**^ | ,215^**^ | ,386^**^ | ,132^**^ | -,271^**^ | ,108^**^ | ,036 | ,112^**^ | ,156^**^ | ,352^**^ | ,137^**^ |
|  |  | Sig. (2-tailed) | ,000 | ,000 |  | ,000 | ,000 | ,000 | ,000 | ,000 | ,000 | ,000 | ,000 | ,000 | ,000 | ,185 | ,000 | ,000 | ,000 | ,000 |
|  |  | N | 1348 | 1348 | 1348 | 1348 | 1348 | 1348 | 1348 | 1348 | 1348 | 1348 | 1348 | 1348 | 1348 | 1348 | 1348 | 1348 | 1348 | 1348 |
| 3. Wilt u uit dit rijtje aangeven over welke onderwerpen u wél gehoord heeft. Er zijn meerdere antwoorden mogelijk. wat de nadelen en risico's van het bevolkingsonderzoek darmkanker zijn | | Pearson Correlation | ,197^**^ | ,290^**^ | ,399^**^ | 1 | ,314^**^ | ,225^**^ | ,270^**^ | ,233^**^ | ,320^**^ | ,349^**^ | ,205^**^ | -,129^**^ | -,077^**^ | -,102^**^ | -,111^**^ | -,074^**^ | ,308^**^ | ,100^**^ |
|  |  | Sig. (2-tailed) | ,000 | ,000 | ,000 |  | ,000 | ,000 | ,000 | ,000 | ,000 | ,000 | ,000 | ,000 | ,005 | ,000 | ,000 | ,007 | ,000 | ,000 |
|  |  | N | 1348 | 1348 | 1348 | 1348 | 1348 | 1348 | 1348 | 1348 | 1348 | 1348 | 1348 | 1348 | 1348 | 1348 | 1348 | 1348 | 1348 | 1348 |
| 3. Wilt u uit dit rijtje aangeven over welke onderwerpen u wél gehoord heeft. Er zijn meerdere antwoorden mogelijk. welke kosten er kunnen zijn voor deelnemers aan het bevolkingsonderzoek darmkanker | | Pearson Correlation | ,137^**^ | ,280^**^ | ,218^**^ | ,314^**^ | 1 | ,201^**^ | ,194^**^ | ,191^**^ | ,347^**^ | ,257^**^ | ,290^**^ | -,077^**^ | ,056^*^ | ,003 | ,005 | ,036 | ,208^**^ | ,077^**^ |
|  |  | Sig. (2-tailed) | ,000 | ,000 | ,000 | ,000 |  | ,000 | ,000 | ,000 | ,000 | ,000 | ,000 | ,004 | ,040 | ,921 | ,854 | ,188 | ,000 | ,005 |
|  |  | N | 1348 | 1348 | 1348 | 1348 | 1348 | 1348 | 1348 | 1348 | 1348 | 1348 | 1348 | 1348 | 1348 | 1348 | 1348 | 1348 | 1348 | 1348 |
| 3. Wilt u uit dit rijtje aangeven over welke onderwerpen u wél gehoord heeft. Er zijn meerdere antwoorden mogelijk. dat het bevolkingsonderzoek darmkanker door de overheid wordt aangeboden | | Pearson Correlation | ,154^**^ | ,183^**^ | ,305^**^ | ,225^**^ | ,201^**^ | 1 | ,430^**^ | ,388^**^ | ,257^**^ | ,369^**^ | ,152^**^ | -,254^**^ | ,115^**^ | ,072^**^ | ,122^**^ | ,174^**^ | ,236^**^ | ,129^**^ |
|  |  | Sig. (2-tailed) | ,000 | ,000 | ,000 | ,000 | ,000 |  | ,000 | ,000 | ,000 | ,000 | ,000 | ,000 | ,000 | ,008 | ,000 | ,000 | ,000 | ,000 |
|  |  | N | 1348 | 1348 | 1348 | 1348 | 1348 | 1348 | 1348 | 1348 | 1348 | 1348 | 1348 | 1348 | 1348 | 1348 | 1348 | 1348 | 1348 | 1348 |
| 3. Wilt u uit dit rijtje aangeven over welke onderwerpen u wél gehoord heeft. Er zijn meerdere antwoorden mogelijk. dat mensen zelf beslissen of ze wel of niet mee willen doen aan het bevolkingsonderzoek darmkanker | | Pearson Correlation | ,200^**^ | ,167^**^ | ,327^**^ | ,270^**^ | ,194^**^ | ,430^**^ | 1 | ,476^**^ | ,223^**^ | ,438^**^ | ,129^**^ | -,275^**^ | ,086^**^ | ,042 | ,077^**^ | ,130^**^ | ,273^**^ | ,103^**^ |
|  |  | Sig. (2-tailed) | ,000 | ,000 | ,000 | ,000 | ,000 | ,000 |  | ,000 | ,000 | ,000 | ,000 | ,000 | ,002 | ,119 | ,005 | ,000 | ,000 | ,000 |
|  |  | N | 1348 | 1348 | 1348 | 1348 | 1348 | 1348 | 1348 | 1348 | 1348 | 1348 | 1348 | 1348 | 1348 | 1348 | 1348 | 1348 | 1348 | 1348 |
| 3. Wilt u uit dit rijtje aangeven over welke onderwerpen u wél gehoord heeft. Er zijn meerdere antwoorden mogelijk. dat mensen een ontlastingstest thuisgestuurd krijgen en er gekeken wordt of er in hun ontlasting bloed zit | | Pearson Correlation | ,212^**^ | ,185^**^ | ,350^**^ | ,233^**^ | ,191^**^ | ,388^**^ | ,476^**^ | 1 | ,276^**^ | ,487^**^ | ,142^**^ | -,288^**^ | ,060^*^ | ,040 | ,088^**^ | ,162^**^ | ,283^**^ | ,094^**^ |
|  |  | Sig. (2-tailed) | ,000 | ,000 | ,000 | ,000 | ,000 | ,000 | ,000 |  | ,000 | ,000 | ,000 | ,000 | ,027 | ,146 | ,001 | ,000 | ,000 | ,001 |
|  |  | N | 1348 | 1348 | 1348 | 1348 | 1348 | 1348 | 1348 | 1348 | 1348 | 1348 | 1348 | 1348 | 1348 | 1348 | 1348 | 1348 | 1348 | 1348 |
| 3. Wilt u uit dit rijtje aangeven over welke onderwerpen u wél gehoord heeft. Er zijn meerdere antwoorden mogelijk. informatie over de kwaliteit van de ontlastingstest | | Pearson Correlation | ,145^**^ | ,350^**^ | ,215^**^ | ,320^**^ | ,347^**^ | ,257^**^ | ,223^**^ | ,276^**^ | 1 | ,326^**^ | ,256^**^ | -,097^**^ | ,099^**^ | ,008 | ,074^**^ | ,127^**^ | ,271^**^ | ,096^**^ |
|  |  | Sig. (2-tailed) | ,000 | ,000 | ,000 | ,000 | ,000 | ,000 | ,000 | ,000 |  | ,000 | ,000 | ,000 | ,000 | ,766 | ,007 | ,000 | ,000 | ,000 |
|  |  | N | 1348 | 1348 | 1348 | 1348 | 1348 | 1348 | 1348 | 1348 | 1348 | 1348 | 1348 | 1348 | 1348 | 1348 | 1348 | 1348 | 1348 | 1348 |
| 3. Wilt u uit dit rijtje aangeven over welke onderwerpen u wél gehoord heeft. Er zijn meerdere antwoorden mogelijk. dat mensen eventueel een vervolgonderzoek (coloscopie) moeten laten doen en wat er dan gebeurt | | Pearson Correlation | ,254^**^ | ,272^**^ | ,386^**^ | ,349^**^ | ,257^**^ | ,369^**^ | ,438^**^ | ,487^**^ | ,326^**^ | 1 | ,227^**^ | -,197^**^ | ,126^**^ | ,024 | ,101^**^ | ,158^**^ | ,378^**^ | ,084^**^ |
|  |  | Sig. (2-tailed) | ,000 | ,000 | ,000 | ,000 | ,000 | ,000 | ,000 | ,000 | ,000 |  | ,000 | ,000 | ,000 | ,382 | ,000 | ,000 | ,000 | ,002 |
|  |  | N | 1348 | 1348 | 1348 | 1348 | 1348 | 1348 | 1348 | 1348 | 1348 | 1348 | 1348 | 1348 | 1348 | 1348 | 1348 | 1348 | 1348 | 1348 |
| 3. Wilt u uit dit rijtje aangeven over welke onderwerpen u wél gehoord heeft. Er zijn meerdere antwoorden mogelijk. wachttijden voor een coloscopie | | Pearson Correlation | ,065^*^ | ,227^**^ | ,132^**^ | ,205^**^ | ,290^**^ | ,152^**^ | ,129^**^ | ,142^**^ | ,256^**^ | ,227^**^ | 1 | -,053 | ,055^*^ | -,011 | ,031 | ,077^**^ | ,178^**^ | ,065^*^ |
|  |  | Sig. (2-tailed) | ,016 | ,000 | ,000 | ,000 | ,000 | ,000 | ,000 | ,000 | ,000 | ,000 |  | ,051 | ,044 | ,682 | ,256 | ,005 | ,000 | ,016 |
|  |  | N | 1348 | 1348 | 1348 | 1348 | 1348 | 1348 | 1348 | 1348 | 1348 | 1348 | 1348 | 1348 | 1348 | 1348 | 1348 | 1348 | 1348 | 1348 |
| 3. Wilt u uit dit rijtje aangeven over welke onderwerpen u wél gehoord heeft. Er zijn meerdere antwoorden mogelijk. geen van deze | | Pearson Correlation | -,409^**^ | -,117^**^ | -,271^**^ | -,129^**^ | -,077^**^ | -,254^**^ | -,275^**^ | -,288^**^ | -,097^**^ | -,197^**^ | -,053 | 1 | -,102^**^ | -,020 | -,079^**^ | -,105^**^ | -,185^**^ | -,017 |
|  |  | Sig. (2-tailed) | ,000 | ,000 | ,000 | ,000 | ,004 | ,000 | ,000 | ,000 | ,000 | ,000 | ,051 |  | ,000 | ,468 | ,004 | ,000 | ,000 | ,535 |
|  |  | N | 1348 | 1348 | 1348 | 1348 | 1348 | 1348 | 1348 | 1348 | 1348 | 1348 | 1348 | 1348 | 1348 | 1348 | 1348 | 1348 | 1348 | 1348 |
| 25. Vindt u het, alles bij elkaar genomen, goed dat in Nederland het bevolkingsonderzoek darmkanker wordt aangeboden? | | Pearson Correlation | ,108^**^ | ,073^**^ | ,108^**^ | -,077^**^ | ,056^*^ | ,115^**^ | ,086^**^ | ,060^*^ | ,099^**^ | ,126^**^ | ,055^*^ | -,102^**^ | 1 | ,533^**^ | ,593^**^ | ,571^**^ | ,157^**^ | ,033 |
|  |  | Sig. (2-tailed) | ,000 | ,007 | ,000 | ,005 | ,040 | ,000 | ,002 | ,027 | ,000 | ,000 | ,044 | ,000 |  | ,000 | ,000 | ,000 | ,000 | ,182 |
|  |  | N | 1348 | 1348 | 1348 | 1348 | 1348 | 1348 | 1348 | 1348 | 1348 | 1348 | 1348 | 1348 | 1679 | 1679 | 1679 | 1679 | 1679 | 1679 |
| steun_gedwkeuze_schaal_hercod_1tot5 | | Pearson Correlation | ,074^**^ | ,015 | ,036 | -,102^**^ | ,003 | ,072^**^ | ,042 | ,040 | ,008 | ,024 | -,011 | -,020 | ,533^**^ | 1 | ,445^**^ | ,436^**^ | ,126^**^ | ,082^**^ |
|  |  | Sig. (2-tailed) | ,006 | ,578 | ,185 | ,000 | ,921 | ,008 | ,119 | ,146 | ,766 | ,382 | ,682 | ,468 | ,000 |  | ,000 | ,000 | ,000 | ,001 |
|  |  | N | 1348 | 1348 | 1348 | 1348 | 1348 | 1348 | 1348 | 1348 | 1348 | 1348 | 1348 | 1348 | 1679 | 1679 | 1679 | 1679 | 1679 | 1679 |
| collectieve_attitude | | Pearson Correlation | ,107^**^ | ,064^*^ | ,112^**^ | -,111^**^ | ,005 | ,122^**^ | ,077^**^ | ,088^**^ | ,074^**^ | ,101^**^ | ,031 | -,079^**^ | ,593^**^ | ,445^**^ | 1 | ,778^**^ | ,164^**^ | ,056^*^ |
|  |  | Sig. (2-tailed) | ,000 | ,019 | ,000 | ,000 | ,854 | ,000 | ,005 | ,001 | ,007 | ,000 | ,256 | ,004 | ,000 | ,000 |  | ,000 | ,000 | ,022 |
|  |  | N | 1348 | 1348 | 1348 | 1348 | 1348 | 1348 | 1348 | 1348 | 1348 | 1348 | 1348 | 1348 | 1679 | 1679 | 1679 | 1679 | 1679 | 1679 |
| persoonlijke_attitude | | Pearson Correlation | ,115^**^ | ,088^**^ | ,156^**^ | -,074^**^ | ,036 | ,174^**^ | ,130^**^ | ,162^**^ | ,127^**^ | ,158^**^ | ,077^**^ | -,105^**^ | ,571^**^ | ,436^**^ | ,778^**^ | 1 | ,237^**^ | ,062^*^ |
|  |  | Sig. (2-tailed) | ,000 | ,001 | ,000 | ,007 | ,188 | ,000 | ,000 | ,000 | ,000 | ,000 | ,005 | ,000 | ,000 | ,000 | ,000 |  | ,000 | ,011 |
|  |  | N | 1348 | 1348 | 1348 | 1348 | 1348 | 1348 | 1348 | 1348 | 1348 | 1348 | 1348 | 1348 | 1679 | 1679 | 1679 | 1679 | 1679 | 1679 |
| schaal_subjectievekennis | | Pearson Correlation | ,232^**^ | ,260^**^ | ,352^**^ | ,308^**^ | ,208^**^ | ,236^**^ | ,273^**^ | ,283^**^ | ,271^**^ | ,378^**^ | ,178^**^ | -,185^**^ | ,157^**^ | ,126^**^ | ,164^**^ | ,237^**^ | 1 | ,069^**^ |
|  |  | Sig. (2-tailed) | ,000 | ,000 | ,000 | ,000 | ,000 | ,000 | ,000 | ,000 | ,000 | ,000 | ,000 | ,000 | ,000 | ,000 | ,000 | ,000 |  | ,004 |
|  |  | N | 1348 | 1348 | 1348 | 1348 | 1348 | 1348 | 1348 | 1348 | 1348 | 1348 | 1348 | 1348 | 1679 | 1679 | 1679 | 1679 | 1679 | 1679 |
| kennisvragenjuist | | Pearson Correlation | ,086^**^ | ,063^*^ | ,137^**^ | ,100^**^ | ,077^**^ | ,129^**^ | ,103^**^ | ,094^**^ | ,096^**^ | ,084^**^ | ,065^*^ | -,017 | ,033 | ,082^**^ | ,056^*^ | ,062^*^ | ,069^**^ | 1 |
|  |  | Sig. (2-tailed) | ,002 | ,021 | ,000 | ,000 | ,005 | ,000 | ,000 | ,001 | ,000 | ,002 | ,016 | ,535 | ,182 | ,001 | ,022 | ,011 | ,004 |  |
|  |  | N | 1348 | 1348 | 1348 | 1348 | 1348 | 1348 | 1348 | 1348 | 1348 | 1348 | 1348 | 1348 | 1679 | 1679 | 1679 | 1679 | 1679 | 1679 |
| **. Correlation is significant at the 0.01 level (2-tailed). |  |  |  |  |  |  |  |  |  |  |  |  |  |  |  |  |  |  |  |  |
| *. Correlation is significant at the 0.05 level (2-tailed). |  |  |  |  |  |  |  |  |  |  |  |  |  |  |  |  |  |  |  |  |

| **Correlations** | | | | | | | | | |
| --- | --- | --- | --- | --- | --- | --- | --- | --- | --- |
|  | | geh_bvodk_ja | schaal_subjectievekennis | kennisvragenjuist | persoonlijke_attitude | collectieve_attitude | sociale_norm | 25. Vindt u het, alles bij elkaar genomen, goed dat in Nederland het bevolkingsonderzoek darmkanker wordt aangeboden? | steun_gedwkeuze_schaal_hercod_1tot5 |
| geh_bvodk_ja | Pearson Correlation | 1 | ,453^**^ | ,030 | ,170^**^ | ,079^**^ | ,020 | ,110^**^ | ,099^**^ |
|  | Sig. (2-tailed) |  | ,000 | ,219 | ,000 | ,001 | ,413 | ,000 | ,000 |
|  | N | 1679 | 1679 | 1679 | 1679 | 1679 | 1679 | 1679 | 1679 |
| schaal_subjectievekennis | Pearson Correlation | ,453^**^ | 1 | ,069^**^ | ,237^**^ | ,164^**^ | ,118^**^ | ,157^**^ | ,126^**^ |
|  | Sig. (2-tailed) | ,000 |  | ,004 | ,000 | ,000 | ,000 | ,000 | ,000 |
|  | N | 1679 | 1679 | 1679 | 1679 | 1679 | 1679 | 1679 | 1679 |
| kennisvragenjuist | Pearson Correlation | ,030 | ,069^**^ | 1 | ,062^*^ | ,056^*^ | ,049^*^ | ,033 | ,082^**^ |
|  | Sig. (2-tailed) | ,219 | ,004 |  | ,011 | ,022 | ,044 | ,182 | ,001 |
|  | N | 1679 | 1679 | 1679 | 1679 | 1679 | 1679 | 1679 | 1679 |
| persoonlijke_attitude | Pearson Correlation | ,170^**^ | ,237^**^ | ,062^*^ | 1 | ,778^**^ | ,479^**^ | ,571^**^ | ,436^**^ |
|  | Sig. (2-tailed) | ,000 | ,000 | ,011 |  | ,000 | ,000 | ,000 | ,000 |
|  | N | 1679 | 1679 | 1679 | 1679 | 1679 | 1679 | 1679 | 1679 |
| collectieve_attitude | Pearson Correlation | ,079^**^ | ,164^**^ | ,056^*^ | ,778^**^ | 1 | ,522^**^ | ,593^**^ | ,445^**^ |
|  | Sig. (2-tailed) | ,001 | ,000 | ,022 | ,000 |  | ,000 | ,000 | ,000 |
|  | N | 1679 | 1679 | 1679 | 1679 | 1679 | 1679 | 1679 | 1679 |
| sociale_norm | Pearson Correlation | ,020 | ,118^**^ | ,049^*^ | ,479^**^ | ,522^**^ | 1 | ,508^**^ | ,328^**^ |
|  | Sig. (2-tailed) | ,413 | ,000 | ,044 | ,000 | ,000 |  | ,000 | ,000 |
|  | N | 1679 | 1679 | 1679 | 1679 | 1679 | 1679 | 1679 | 1679 |
| 25. Vindt u het, alles bij elkaar genomen, goed dat in Nederland het bevolkingsonderzoek darmkanker wordt aangeboden? | Pearson Correlation | ,110^**^ | ,157^**^ | ,033 | ,571^**^ | ,593^**^ | ,508^**^ | 1 | ,533^**^ |
|  | Sig. (2-tailed) | ,000 | ,000 | ,182 | ,000 | ,000 | ,000 |  | ,000 |
|  | N | 1679 | 1679 | 1679 | 1679 | 1679 | 1679 | 1679 | 1679 |
| steun_gedwkeuze_schaal_hercod_1tot5 | Pearson Correlation | ,099^**^ | ,126^**^ | ,082^**^ | ,436^**^ | ,445^**^ | ,328^**^ | ,533^**^ | 1 |
|  | Sig. (2-tailed) | ,000 | ,000 | ,001 | ,000 | ,000 | ,000 | ,000 |  |
|  | N | 1679 | 1679 | 1679 | 1679 | 1679 | 1679 | 1679 | 1679 |
| **. Correlation is significant at the 0.01 level (2-tailed). | | | | | | | | | |
| *. Correlation is significant at the 0.05 level (2-tailed). | | | | | | | | | |

| **Correlations** | | | | | | | | | |
| --- | --- | --- | --- | --- | --- | --- | --- | --- | --- |
|  | | 10. Hoeveel weet u volgens u zelf over: De nadelen en risico's van het bevolkingsonderzoek darmkanker. | 10. Hoeveel weet u volgens u zelf over: De voordelen van het bevolkingsonderzoek darmkanker. | 10. Hoeveel weet u volgens u zelf over: De ontlastingstest en het vervolgonderzoek van het bevolkingsonderzoek darmkanker. | 25. Vindt u het, alles bij elkaar genomen, goed dat in Nederland het bevolkingsonderzoek darmkanker wordt aangeboden? | collectieve_attitude | persoonlijke_attitude | steun_gedwkeuze_schaal_hercod_1tot5 | kennisvragenjuist |
| 10. Hoeveel weet u volgens u zelf over: De nadelen en risico's van het bevolkingsonderzoek darmkanker. | Pearson Correlation | 1 | ,597^**^ | ,677^**^ | ,053^*^ | ,043 | ,094^**^ | ,053^*^ | ,036 |
|  | Sig. (2-tailed) |  | ,000 | ,000 | ,029 | ,081 | ,000 | ,030 | ,140 |
|  | N | 1679 | 1679 | 1679 | 1679 | 1679 | 1679 | 1679 | 1679 |
| 10. Hoeveel weet u volgens u zelf over: De voordelen van het bevolkingsonderzoek darmkanker. | Pearson Correlation | ,597^**^ | 1 | ,730^**^ | ,205^**^ | ,224^**^ | ,275^**^ | ,156^**^ | ,088^**^ |
|  | Sig. (2-tailed) | ,000 |  | ,000 | ,000 | ,000 | ,000 | ,000 | ,000 |
|  | N | 1679 | 1679 | 1679 | 1679 | 1679 | 1679 | 1679 | 1679 |
| 10. Hoeveel weet u volgens u zelf over: De ontlastingstest en het vervolgonderzoek van het bevolkingsonderzoek darmkanker. | Pearson Correlation | ,677^**^ | ,730^**^ | 1 | ,157^**^ | ,169^**^ | ,256^**^ | ,125^**^ | ,061^*^ |
|  | Sig. (2-tailed) | ,000 | ,000 |  | ,000 | ,000 | ,000 | ,000 | ,013 |
|  | N | 1679 | 1679 | 1679 | 1679 | 1679 | 1679 | 1679 | 1679 |
| 25. Vindt u het, alles bij elkaar genomen, goed dat in Nederland het bevolkingsonderzoek darmkanker wordt aangeboden? | Pearson Correlation | ,053^*^ | ,205^**^ | ,157^**^ | 1 | ,593^**^ | ,571^**^ | ,533^**^ | ,033 |
|  | Sig. (2-tailed) | ,029 | ,000 | ,000 |  | ,000 | ,000 | ,000 | ,182 |
|  | N | 1679 | 1679 | 1679 | 1679 | 1679 | 1679 | 1679 | 1679 |
| collectieve_attitude | Pearson Correlation | ,043 | ,224^**^ | ,169^**^ | ,593^**^ | 1 | ,778^**^ | ,445^**^ | ,056^*^ |
|  | Sig. (2-tailed) | ,081 | ,000 | ,000 | ,000 |  | ,000 | ,000 | ,022 |
|  | N | 1679 | 1679 | 1679 | 1679 | 1679 | 1679 | 1679 | 1679 |
| persoonlijke_attitude | Pearson Correlation | ,094^**^ | ,275^**^ | ,256^**^ | ,571^**^ | ,778^**^ | 1 | ,436^**^ | ,062^*^ |
|  | Sig. (2-tailed) | ,000 | ,000 | ,000 | ,000 | ,000 |  | ,000 | ,011 |
|  | N | 1679 | 1679 | 1679 | 1679 | 1679 | 1679 | 1679 | 1679 |
| steun_gedwkeuze_schaal_hercod_1tot5 | Pearson Correlation | ,053^*^ | ,156^**^ | ,125^**^ | ,533^**^ | ,445^**^ | ,436^**^ | 1 | ,082^**^ |
|  | Sig. (2-tailed) | ,030 | ,000 | ,000 | ,000 | ,000 | ,000 |  | ,001 |
|  | N | 1679 | 1679 | 1679 | 1679 | 1679 | 1679 | 1679 | 1679 |
| kennisvragenjuist | Pearson Correlation | ,036 | ,088^**^ | ,061^*^ | ,033 | ,056^*^ | ,062^*^ | ,082^**^ | 1 |
|  | Sig. (2-tailed) | ,140 | ,000 | ,013 | ,182 | ,022 | ,011 | ,001 |  |
|  | N | 1679 | 1679 | 1679 | 1679 | 1679 | 1679 | 1679 | 1679 |
| **. Correlation is significant at the 0.01 level (2-tailed). | | | | | | | | | |
| *. Correlation is significant at the 0.05 level (2-tailed). | | | | | | | | | |
